# Free-Ranging Cat Predation on Wildlife in East Asia: A Scoping Review of Ecological Evidence and Knowledge Gaps

**DOI:** 10.3390/ani16203321

**Published:** 2026-10-21

**Authors:** Michelle Zhibing Zhou, Christian S. Wright

**Affiliations:** 1 Cranbrook Schools, Bloomfield Hills, MI 48304, USA; 2 Berkwood Research Institute, Santa Barbara, CA 93101, USA

**Keywords:** *Felis catus*, invasive species, predation, biodiversity, East Asia, scoping review, prey populations, island ecology, trap–neuter–return, wildlife conservation

## Abstract

Free-ranging domestic cats (*Felis catus*) interact with wildlife through predation and other ecological pathways, yet evidence of their associated effects has not been comprehensively synthesized in East Asia. This scoping review mapped empirical evidence on free-ranging cat predation and associated ecological effects in East Asia, characterized its geographic distribution and inferential strength, and identified knowledge gaps. Five databases, including region-specific databases, were searched through June 2026, and reference lists of included full-text sources were hand-searched. Of 9326 records identified, 31 sources met the inclusion criteria: 28 from the pre-specified East Asian region and 3 global or multinational sources with independently extractable East Asia-specific data. Diet- and carcass-based studies documented consumption of diverse wildlife taxa, whereas fewer evaluated prey abundance or other population-level outcomes. Reported predation estimates varied substantially among studies, reflecting differences in cat category, ecological setting, and methodology. Evidence was concentrated in Japan, with the clearest conservation concerns reported from Japanese island ecosystems involving endemic or IUCN-listed threatened species. Human-mediated factors and management practices were recurrent contextual themes; however, direct evidence linking these factors to wildlife outcomes was limited. Overall, the evidence base was geographically and methodologically uneven, underscoring the need for standardization, broader regional coverage, and improved documentation.

## Simple Summary

Domestic cats (*Felis catus*) interact with wildlife through predation and other ecological pathways, yet these interactions and their effects have not been comprehensively synthesized in East Asia. We conducted a scoping review using five databases to characterize the available evidence on free-ranging cat predation across the region. Studies documented predation on diverse wildlife taxa, with notable conservation concerns in island ecosystems supporting endemic or threatened species. Most studies focused on diet, prey occurrence, or predation frequency, while relatively few assessed changes in prey abundance or other population-level outcomes. Human factors, including supplemental feeding and shelter provision, together with public attitudes and local regulatory frameworks, were also identified as factors associated with cat distribution, persistence, and opportunities for interaction with wildlife. Overall, the evidence base was geographically and methodologically uneven, highlighting the need for more standardized study designs, broader regional coverage, and improved reporting of prey taxa, ecological responses, and relevant human-context variables.

## 1. Introduction

Domestic cats (*Felis catus*, hereafter “cats”) were domesticated approximately 10,000 years ago in the Fertile Crescent, wherein their association with human settlements likely facilitated rodent control around grain stores [1]. Today, cats appear on nearly every continent and are among the most widely distributed carnivores globally [2]. Historically valued both as companion animals and pest controllers, cats are now predominantly kept as pets, with an estimated 200 million pet cats worldwide. Their high prevalence, coupled with widespread outdoor access in many settings, has raised ecological, animal-health, and animal-welfare concerns, including predation, pathogen transmission to native felids, competition with native species, and hybridization with wild felids [3,4].

Free-ranging cats are recognized as an important conservation concern in many regions due to their predation across diverse vertebrate and invertebrate taxa. Classified as an invasive species in multiple regions, particularly within island ecosystems, free-ranging cats have been implicated in the decline and extinction of numerous species [5,6,7]. Large-scale modelling studies have estimated substantial annual predation totals in some countries; for example, an estimated 19–197 million birds are killed annually by free-ranging cats in Canada alone [8]. Despite these documented events, the magnitude and ecological consequences of cat predation vary substantially among regions and may depend on ecological context, prey availability, characteristics of cat populations, and human management practices.

Free-ranging cats are often operationally categorized as owned outdoor, stray or community, and feral cats, although the boundaries among these groups vary across studies and jurisdictions [9,10]. Owned outdoor cats are provisioned by humans but retain some degree of outdoor access, with opportunities for wildlife interaction varying according to the extent and timing of access. Stray cats, though not owned, often remain comfortable around humans and may obtain some of their diet directly or indirectly from humans, potentially reducing their reliance on hunting. In contrast, feral cats are generally less socialized to humans and may rely more heavily on hunting, although habitat use and access to human-provided resources vary among populations [10,11]. Distinguishing among cat populations and their relationships with humans is therefore important when interpreting predation estimates and other ecological evidence.

Extensive syntheses of cat–wildlife interactions have been conducted in Australia [11,12], New Zealand [13], the UK [14,15], and North America [8,16,17], whereas the East Asian evidence base has not been comprehensively synthesized. This gap is relevant as East Asia includes numerous island ecosystems supporting endemic and threatened taxa, which may be particularly vulnerable to newly introduced mammalian predators. In addition, regional sociocultural practices, including communal outdoor feeding, trap–neuter–return (TNR) programs, and attitudes toward free-ranging cats, may influence cat distribution, persistence, and opportunities for interaction with wildlife. As such, these factors represent important contextual considerations when interpreting ecological evidence across the region.

Accordingly, the objective of this scoping review was to systematically map the existing empirical evidence on free-ranging cat predation and its reported ecological effects on wildlife in East Asia. Specifically, we aimed to: (1) characterize the distribution of primary evidence by country or region, habitat, cat category, methodology, and wildlife outcome; (2) distinguish evidence of consumption from direct predation, predation pressure, and wildlife population-level responses; (3) summarize human-mediated contextual evidence, including provisioning, TNR programs, public attitudes, and management implementation in relation to cat presence and predation risk; and (4) identify geographic and methodological knowledge gaps to inform future research priorities in the region.

## 2. Materials and Methods

### 2.1. Protocol and Checklist

This scoping review was designed to systematically map the extent, characteristics, and gaps within the available evidence, consistent with established scoping review methodology. This review was conducted in accordance with the Preferred Reporting Items for Systematic Reviews and Meta-Analyses extension for Scoping Reviews (PRISMA-ScR) and established methodological guidance, including the Arksey and O’Malley framework and Joanna Briggs Institute recommendations [18,19,20]. To ensure transparency and a priori planning, the review protocol was prospectively registered on the Open Science Framework (OSF) on 20 September 2025 (https://osf.io/v58ts) and the completed PRISMA-ScR checklist is provided as Supplementary Table S1.

Three deviations from the registered protocol occurred and are reported transparently. First, although the China National Knowledge Infrastructure (CNKI) and KoreaScience databases were specified in the protocol, access constraints prevented their inclusion during the search period; the implications of this omission are addressed in the Limitations section. Second, the protocol did not specify how global or multinational sources containing independently extractable East Asian data should be handled. A post hoc eligibility criterion was therefore introduced and applied consistently during source re-adjudication. Third, classification of included sources by inferential evidence level was added following protocol registration to distinguish consumption and diet evidence from predation-pressure and wildlife population-response evidence.

These modifications were introduced to improve transparency and interpretability of the evidence base without altering the review’s pre-specified geographic scope or primary objective of mapping free-ranging cat–wildlife evidence in East Asia. All protocol deviations are documented in the OSF record.

### 2.2. Review Question

The scope of this review was defined using the “Population”, “Concept”, and “Context” framework (PCC). The pre-specified framework was revised to more clearly distinguish evidence of wildlife consumption and predation from evidence of population-level ecological effects while treating human-mediated factors as a separate contextual evidence stream. This distinction was introduced to avoid interpreting fundamentally different ecological outcomes as equivalent.

- Population (P)—Free-ranging domestic cats residing in East Asia, including owned outdoor, stray/community, feral, mixed, or otherwise unspecified free-ranging populations.
- Concept (C)—Empirical evidence on cat predation and associated wildlife responses, classified as: (1) consumption/diet evidence; (2) direct predation or predation-pressure evidence; and (3) wildlife population-level responses. Human-mediated factors, including provisioning, ownership, public attitudes, TNR, and other management practices, were treated separately as contextual evidence unless a wildlife outcome was directly measured.
- Context (C)—Studies conducted within the pre-specified East Asia region, defined as Japan, South Korea, Mongolia, Mainland China, Hong Kong, Taiwan, and Far Eastern Russia. Studies conducted outside this geographic context were not considered part of the regional evidence base. Global or multinational sources were eligible only when East Asia-specific data could be independently extracted and evaluated.

Drawing on these PCC elements, the guiding research question for this scoping review was:

*What is the extent, distribution, and inferential strength of the available evidence on free-ranging cat predation and associated wildlife responses in East Asia?*

### 2.3. Inclusion and Exclusion Criteria

Studies were eligible if they reported empirical evidence relevant to either (1) free-ranging cat predation or associated wildlife responses or (2) pre-specified human-mediated contextual factors relevant to cat–wildlife interactions within the East Asian region. Studies were included if they were conducted in East Asia (as defined above) and were peer-reviewed publications or government/NGO technical reports with accessible empirical data. Publications written in English or in relevant regional languages (Japanese, Chinese, or Korean) were included. To capture all available evidence, no date restrictions were applied.

A separate contextual evidence stream included primary empirical studies conducted within the same geographic region that examined human-mediated factors relevant to free-ranging cat presence, persistence, movement, or opportunities for wildlife interaction. Eligible contextual outcomes included provisioning and shelter, ownership practices, public attitudes, TNR or other management interventions, and implementation of relevant policies or regulations. These studies were retained to characterize the human context surrounding cat–wildlife interactions but were not interpreted as evidence of wildlife benefit or harm unless a wildlife outcome was directly measured.

Studies were excluded if they: (1) were conducted outside the defined East Asian region without independently extractable East Asia-specific data; (2) focused exclusively on confined or laboratory cats without free-ranging exposure; or (3) consisted of opinion pieces, editorials, policy analyses, narrative reviews, or other publications lacking relevant empirical data. Global or multinational sources were retained only when East Asia-specific data could be independently extracted; otherwise, they were used solely as contextual literature.

### 2.4. Informational Sources

Five scientific databases were searched: PubMed, Web of Science, Scopus, J-Stage, and CiNii Research (Table 1). J-Stage and CiNii were included specifically to capture Japanese-language literature that may not be indexed in international databases. Reference lists of studies retained for full-text review were also hand-searched to identify any additional relevant sources not captured in the database searches. All database searches were originally executed between October 2025 and January 2026; the updated final search was conducted in June 2026 prior to peer review.

Although KoreaScience and the China National Knowledge Infrastructure (CNKI) were specified in the registered protocol, access limitations during the search period prevented their inclusion. As a result, regional-language database coverage was more comprehensive for Japan than for China or South Korea. This constraint is acknowledged as a limitation and is considered explicitly when interpreting the geographic distribution of the retrieved evidence.

### 2.5. Search Strategy

Search strategies were tailored to each database using structured Boolean search strings, combining three concept domains: (1) cat terms (Felis catus, cat*, feral cat*, stray cat*, free-ranging cat*); (2) predation or wildlife outcomes (e.g., predation, hunting, prey, diet, mortality, wildlife impact); and (3) ecological context (e.g., ecology, biodiversity, conservation, invasive). Searches were intentionally broad to maximize sensitivity, and geographic eligibility was primarily determined during title/abstract and full-text screening rather than through restrictive geographic search terms. No date or document-type filters were applied. English-language filters were used for PubMed, Web of Science, and Scopus, while Japanese-language literature was searched through J-Stage and CiNii Research.

For J-Stage and CiNii Research, equivalent search concepts were translated into Japanese using machine translation and reviewed by a team member with Japanese reading proficiency. Key Japanese search terms included イエネコ (野豚化 ネコ) [feral/wild cat] and 捕食 [捕食者] [predation/prey]. The search strategy was not formally peer-reviewed using the PRESS checklist, which is acknowledged as a methodological limitation.

The full PubMed search strategy is presented below and serves as a representative example (Table 2). The complete search strategies for all databases are provided in Supplementary Table S2.

### 2.6. Selection and Evaluation of Studies

Study selection followed a two-stage process. All records from database searches were imported into Rayyan (Intelligent Systematic Review) for deduplication and screening management. Duplicate records were identified and removed automatically and then verified manually.

In Stage 1 (title and abstract screening), to establish inter-rater reliability prior to full screening (Stage 2), both reviewers independently screened a randomly selected 67% subset of records (*n* = 3988). Cohen’s Kappa (κ) was calculated after this pilot exercise; a target of κ ≥ 0.70 was specified in the protocol as indicating substantial agreement [21]. Observed inter-reviewer agreement during calibration was κ = 0.956. Following the calibration exercise, disagreements were discussed, and the screening criteria were refined where necessary. One reviewer (MZ) then completed screening of all remaining records, with the second reviewer (CSW) verifying a random 10% sample of excluded records to check for systematic errors.

In Stage 2 (full-text review), the full text of all records not excluded at Stage 1 were retrieved and independently assessed by both reviewers against the inclusion and exclusion criteria. Disagreements were resolved through discussion and consensus; no arbitration by a third party was required. All exclusion reasons were documented for every full-text record assessed, and these are reported in the PRISMA-ScR flow diagram (Figure 1) and summarized in the Results Sections.

### 2.7. Data Collection Process

A standardized data collection form was jointly developed by both reviewers (MZ and CSW) prior to data extraction, based on the pre-specified data items listed in the registered protocol. Data collection was conducted primarily by one reviewer (MZ) using a structured Google Sheets spreadsheet. The second reviewer (CSW) independently verified collected data for a random 20% sample of included studies, comparing entries variable by variable. Discrepancies were resolved by consensus discussion. The final collected dataset, along with the data collected from template and variable definitions, is deposited openly on OSF as part of the registered protocol materials (https://osf.io/v58ts).

### 2.8. Data Items

The following variables were collected for each included source of evidence (Table 3). Where information was unclear or not reported, this was recorded as “NR” (not reported) rather than making assumptions.

### 2.9. Evidence Classification and Interpretive Framework

Consistent with standard scoping review methodology and the purpose of this review, which is to map the extent and nature of available evidence rather than to appraise its quality, formal critical appraisal of individual sources of evidence was not conducted [18,19,20]. To improve evidence interpretation, each source was classified according to the type of ecological evidence it provided, and the strength of the conclusions supported by the measured outcome. Level 1 included consumption and diet evidence, such as scat or stomach contents, prey remains, and prey returns. These data document prey use or consumption but do not, by themselves, establish predation pressure or population-level effects. Level 2 included direct predation and predation-pressure evidence, such as observed kills, carcass attribution, kill-rate estimates, predation frequency, and modeled mortality. Level 3 included wildlife population-level responses, such as changes in abundance, survival, recruitment, breeding success, population viability, or recovery following cat management.

Human-mediated factors, including provisioning, cat distribution, public attitudes, and management implementation, were classified separately as contextual evidence. These factors were not interpreted as demonstrating wildlife benefit or harm unless a wildlife outcome was directly measured. This framework was intended to distinguish the scope of conclusions supported by the evidence from the overall methodological quality of the study. Evidence level classifications were independently reviewed by both authors, and disagreements were resolved by consensus.

### 2.10. Evidence Synthesis

Results were synthesized descriptively, consistent with the objectives of a scoping review. Given the significant differences in cat category, ecological setting, sampling duration, methodology, and outcome definitions, quantitative findings were not pooled or annualized by review authors. Instead, quantitative findings were retained in the units and timeframes reported by the original studies.

The synthesis was organized into three components: (1) evidence mapping, describing the distribution of sources by geographic region, cat category, habitat, methodology, and evidence level; (2) predation estimates, presenting study-specific quantitative findings; and (3) qualitative narrative synthesis, summarizing ecological interactions and human-mediated contextual factors, including provisioning, shelter, TNR, public attitudes, and management practices.

## 3. Results

### 3.1. Search Results

The study-selection process is summarized in the PRISMA-ScR flow diagram (Figure 1). The database searches identified 9326 articles across five databases: PubMed (*n* = 528), Web of Science (*n* = 1272), Scopus (*n* = 3418), J-Stage (*n* = 3862), and CiNii Research (*n* = 246). After removal of 3408 duplicates using Rayyan (Intelligent Systematic Review), 5918 unique records remained for Stage 1 title and abstract screening. Following Stage 1 screening, 5747 articles were excluded for not meeting the inclusion criteria, leaving 171 articles for full-text retrieval.

Full texts were successfully obtained for 169 articles, while two could not be retrieved. Following the Stage 2 full-text assessment, 138 reports were excluded as they lacked relevant cat data (*n* = 72), lacked predation or prey-related data (*n* = 44), contained insufficient geographic information to confirm eligibility or extractable East Asia-specific data (*n* = 19), consisted of isolated predation case reports (*n* = 2), or contained duplicate or overlapping information (*n* = 1). Reports excluded for lack of predation data primarily addressed cat population dynamics, disease, or behavior without reporting prey-related ecological outcomes. A complete list of excluded studies and reasons for exclusion is provided in Supplementary Table S3.

A total of 31 studies were included in the final evidence synthesis. Of these, 26 were conducted in Japan, 2 in China, and 3 global or multinational studies from which East Asia-specific data could be independently extracted. The full list of included studies is provided in Supplementary Table S4.

### 3.2. Study Characteristics

The characteristics of all 31 included studies are summarized in Table 4. Studies were published between 2002 and 2026, with the majority published after 2010, indicating that much of the evidence identified in this review is relatively recent.

The evidence identified in this review was concentrated in Japan, accounting for 26 of the 31 included studies (84%) compared to the two studies from China (6.5%). No eligible primary studies were identified from South Korea, Mongolia, Hong Kong, Taiwan, or Far Eastern Russia. However, this geographic pattern should be interpreted with caution as regional database coverage was uneven. Japanese-language literature was searched directly through J-Stage and CiNii Research, whereas the planned CNKI and KoreaScience database searches could not be completed due to access limitations. Consequently, the predominance of Japanese studies in the retrieved evidence base may reflect both differences in research activity and variation in database and language accessibility, rather than the true geographic distribution of research across East Asia.

Island environments represented the predominant ecological setting among the Japan-based studies, accounting for 23 of the 26 studies (88.5%), with the remaining studies conducted in urban, rural, or other mainland settings. Feral cats were the most frequently studied cat category (*n* = 20), whereas the China-based studies included owned cats in urban and rural settings.

Dietary and predation evidence was assessed using several methodological approaches. Fecal or carcass analysis was the most frequently used approach (*n* = 14) and primarily provided evidence of prey consumption or diet composition rather than direct observation of predation events. Camera trapping was also commonly employed (*n* = 8), while questionnaire surveys (*n* = 5) were used primarily to characterize cat ownership, human behavior, public attitudes, and other human-mediated factors relevant to cat–wildlife interactions. As some studies employed more than one methodological approach, method categories were not necessarily mutually exclusive.

When classified by ecological evidence level, most studies provided Level 1 or Level 2 evidence, documenting prey consumption, direct predation, or predation pressure, whereas relatively few evaluated Level 3 population-level responses. Of the 31 included studies, 7 studies provided Level 1 evidence, 7 studies provided Level 2 evidence, and 3 studies evaluated Level 3 wildlife population responses. The remaining 14 studies were focused on human-mediated factors, and thus would only briefly touch on predation.

### 3.3. Synthesis of Results

#### 3.3.1. Quantitative Evidence of Cat Predation and Wildlife Population Responses in East Asia

Quantitative measures of cat predation and prey consumption varied substantially across studies in both magnitude and scale, reflecting differences in cat category, habitat, sampling duration, methodology, and the denominator used for reporting. As shown in Table 5, estimates ranged from short-duration observations of individual prey losses to annual study-level or population-level estimates, including survey- and model-derived values. Since these outcomes were reported over different timeframes and at different analytical scales, outcomes were retained in their original units rather than generating a pooled or standardized unit for study comparisons.

Across the included studies, free-ranging cats were documented consuming or killing a diverse range of vertebrate prey, including birds, mammals, reptiles, amphibians, and fish. However, most quantitative studies characterized diet composition, prey occurrence, observed kills, or estimated predation pressure, rather than measuring changes in prey abundance, survival, recruitment, or population trajectory. Accordingly, the available evidence demonstrates taxonomically broad cat–wildlife interactions in East Asia; however, it generally does not establish the extent to which these interactions translate into population-level effects. Of the 31 included studies, only three directly evaluated wildlife population responses associated with cat presence, predation, or cat-control interventions.

Species-level prey reporting was inconsistent across the included studies, limiting characterization of the full taxonomic breadth of wildlife consumed or preyed upon by free-ranging cats within East Asia. Lepczyk et al. [7], a global synthesis of cat diet, identified 141 prey species from studies classified broadly within Asia; however, the published data do not permit independent extraction of records specifically from the East Asian region defined in this review. This estimate therefore provides broader regional context rather than an East Asia-specific measure of prey diversity. To our knowledge, no previous synthesis has comprehensively characterized the diversity of prey species documented in free-ranging cat diets across East Asia.

#### 3.3.2. Qualitative & Contextual Evidence on Cat–Wildlife Interactions and Management in East Asia

Qualitative findings from the included studies indicated that cat–wildlife interactions in East Asia extended beyond direct predation and were shaped by a combination of ecological interactions, human support of free-ranging cat populations, public attitudes, and management practices. To improve interpretability, these findings were organized into discrete thematic categories.

Indirect ecological interactions and threats to native felids (n = 4)

Three studies documented ecological concerns that extended beyond direct predation of wildlife [29,30,31]. In Japan, these included dietary overlap and potential competition between domestic cats and the endemic Iriomote cat (*Prionailurus bengalensis iriomotensis*) for shared prey resources on Iriomote Island. Hybridization between domestic cats and native felids, including the Tsushima leopard cat (*Prionailurus bengalensis euptilura*) and Iriomote cat, has also been documented, along with interspecific transmission of feline immunodeficiency virus (FIV). These interactions represent additional conservation concerns as they may affect native felids through competition, disease transmission, or loss of genetic distinctiveness rather than through predation alone. However, this evidence was concentrated in Japanese island ecosystems and may not be representative of East Asia more broadly.

Human provisioning, shelter, and cat distribution (n = 5)

Human provisioning and access to shelter were among the most frequently reported human-mediated factors associated with the distribution, persistence, and hunting opportunities of free-ranging cats. Five included studies specifically identified supplemental feeding, housing, or other forms of human support as ecologically relevant factors [32,33,34,35,36,37]. The most detailed example was reported from Tokunoshima Island, where Maeda et al. [34] described “human-driven hyper-predation.” In this system, cats whose primary energetic needs were supported through human feeding continued to hunt endangered endemic wildlife within protected forest habitat. This finding illustrates that human provisioning may sustain cat populations without necessarily eliminating hunting behavior.

Additional evidence from Tokunoshima suggested that housing practices could also increase opportunities for outdoor access and wildlife interaction. One study proposed that the island’s hot and humid climate encouraged residents to leave doors open, allowing owned cats greater access to surrounding outdoor environments where hunting could occur [37]. Cat occurrence was also positively associated with proximity to residential areas, where direct human feeding was more concentrated. Similar patterns were reported in other settings. One study found that higher human population density was associated with greater cat density, particularly near cattle barns and residential areas, which the authors attributed to increased opportunities for feeding, shelter, and human–cat interaction [35].

Human provisioning also appeared to be capable of influencing cat movement and local distribution. In one study, feeding by tourists was associated with the movement of community cats into other areas of the town, suggesting that food availability could alter where cats congregated or relocated [33]. The same study suggested that vacant houses in aging communities may provide shelter and breeding sites, thereby supporting local cat populations. Conversely, restricting food availability did not necessarily eliminate opportunities for cat–wildlife interaction. In Amami City, a regulation prohibiting cat feeding was associated with changes in the abundance and spatial distribution of unowned cats, resulting in increased detection of owned cats within forest interiors [32]. These findings suggest that feeding restrictions may alter cat behavior or distribution rather than their ecological effects. Collectively, the evidence indicates that provisioning and shelter can help sustain free-ranging cat populations and influence where cats occur in relation to vulnerable wildlife, although direct effects on wildlife populations were rarely measured.

Sociocultural perceptions, nuisance concerns, and management acceptability (n = 4)

Five studies examined public attitudes toward free-ranging cats, cat-supportive behaviors, and preferences for management approaches [35,36,37,38]. More favorable attitudes toward cats were generally associated with greater willingness to provide food, although feeding did not necessarily translate into greater willingness to adopt or assume responsibility for stray cats [32]. Public concerns were frequently centered on hygiene, nighttime noise, and other nuisance issues rather than wildlife predation alone. Cat owners also tended to perceive predation as a less serious concern and were less supportive of strict indoor-cat requirements. Overall, non-lethal approaches were generally more acceptable than lethal control, indicating that public perceptions may influence both behaviors that sustain free-ranging cat populations and the feasibility of conservation-oriented management.

Management frameworks, TNR, and policy implementation (n = 12)

Management responses focused primarily on population control, ownership accountability, and reducing outdoor access or movement into ecologically sensitive habitats [33,36,37,39,40,41,42,43,44]. Reported measures included trap–neuter–return (TNR), registration and microchipping, restrictions on outdoor access, removal from sensitive habitats, public education, and responsible-ownership initiatives. Although TNR was frequently discussed, none of the included studies directly demonstrated that TNR reduced or increased wildlife predation. Reported outcomes instead generally concerned cat abundance, colony stability, movement, or program implementation.

Several Japanese studies described more coordinated regional approaches. On Kume Island, local residents, outside organizations, and regional authorities participated in TNR, rescue, and adoption initiatives [25]. In northern Okinawa, a broader conservation-oriented plan sought to remove cats from forest habitats containing endemic wildlife, ensure that cats were owned and identifiable, and prevent their movement into sensitive areas [43]. Measures included wildlife monitoring, registration and neutering requirements, public education, enforcement of relevant regulations, and promotion of responsible ownership. These examples demonstrate how animal population management, ownership accountability, and wildlife conservation can be incorporated within coordinated regional strategies.

Across the limited evidence from Japan and China, sociocultural values and governance also appeared to influence management acceptability. In China, TNR was generally viewed as more humane than lethal control, although greater awareness of ecological impacts was associated with greater acceptance of stronger control measures [36]. Japanese studies similarly emphasized non-lethal management, owner responsibility, and TNR [39,42,44]. As the evidence outside Japan was limited, these observations should be regarded as context-specific rather than representative of national differences.

## 4. Discussion

### 4.1. Principal Findings and Strength of the Evidence

This scoping review systematically mapped the available evidence on free-ranging cat predation and associated wildlife responses in East Asia, identifying 31 eligible studies. Several findings emerged. First, the retrieved evidence was geographically uneven and strongly concentrated in Japan, particularly in island ecosystems. Second, free-ranging cats were documented consuming or killing a taxonomically diverse range of wildlife; however, quantitative estimates varied substantially among studies due to differences in cat populations, category, habitats, sampling duration, study designs, and outcome definitions. Third, species-level prey reporting was inconsistent, limiting characterization of the full taxonomic breadth of cat–wildlife interactions across the region. Finally, human provisioning, sheltering, ownership practices, public attitudes, and management approaches emerged as important human-mediated factors influencing cat persistence, distribution, and opportunities for interaction with wildlife.

An important distinction in interpreting these findings is the strength and scope of the available evidence. The collected evidence primarily documented prey consumption, direct predation, or predation pressure (Evidence Level 1 or 2), while relatively few studies evaluated wildlife population responses (Evidence Level 3). These outcomes and levels of evidence support different conclusions. Dietary records establish consumption, while observed kills and mortality estimates characterize predation at the scale assessed. Neither outcome alone establishes changes in wildlife abundance, survival, recruitment, or population trajectory. Conversely, limited measurement of population responses is not evidence of an absence of these effects. Overall, the collected evidence is more extensive for the documentation of cat–wildlife interactions than for evaluating the population-level consequences of these interactions. This distinction provides an important framework for interpreting the geographic patterns, conservation concerns, and management implications discussed below.

Methodological heterogeneity further limits direct comparisons among studies. Fecal or stomach-content analysis can confirm prey consumption but generally cannot establish when, where, or whether an animal was killed rather than scavenged. Carcass attribution, direct observation, camera-based monitoring, and modeled mortality estimates provide different information and are subject to different detection and sampling biases. These approaches therefore should not be treated as interchangeable measures of ecological impact. In contrast, large-scale assessments from North America, Australia, and the United Kingdom have often combined study-specific predation estimates with cat-density or ownership data and correction factors to generate population-scale estimates [8,11,12,14,16,17]. The current East Asian evidence base does not support comparable regional extrapolation. As a result, this review retained quantitative estimates in the units and timeframes reported by the original studies rather than pooling or independently annualizing them.

### 4.2. Geographic Concentration and Conservation Relevance of Island Systems

The geographic concentration of the evidence requires careful interpretation. Japan accounted for 26 of the 31 included studies, and 23 of the 26 Japan-based studies were conducted in island settings. This concentration likely reflects both the conservation importance of Japanese island ecosystems and the unequal regional database search coverage. Japanese-language literature was directly searched through J-Stage and CiNii Research, whereas CNKI and KoreaScience could not be accessed, and the international databases were searched in English only. As such, the predominance of Japanese studies in this review should not be interpreted as evidence that cat-related research is inherently less common elsewhere in East Asia.

Within the retrieved evidence, consumption or predation involving endemic and threatened wildlife was documented across several Japanese island ecosystems, including the Ogasawara Islands, Tokunoshima, Okinawa, and other Japanese island systems [6,7,22,25,44,45,46,47]. While these observations highlight interactions of conservation concern, their demographic significance depends on factors such as species abundance, life-history characteristics, other sources of mortality, and the extent to which cat-associated mortality is additive rather than compensatory. Although records of consumption and mortality identify species potentially at risk, they cannot on their own determine whether cat-associated mortality contributes to population-level change in Japanese island ecosystems.

Studies measuring wildlife responses provide more direct evidence of population-level consequences [48,49]. For example, the recovery of the Japanese Wood Pigeon (*Columba janthina nitens*) on Chichijima was associated with reductions in feral cat abundance following management [48]. Such studies provide stronger evidence of ecological consequences than diet or kill records alone but should still be interpreted within the study’s design and local context.

### 4.3. Indirect Ecological Interactions and Human-Mediated Factors

The ecological relevance of free-ranging cats also extended beyond direct predation. Japanese studies documented interactions with native wild felids, including dietary overlap and potential competition with the endemic Iriomote cat (*Prionailurus bengalensis iriomotensis)* on Iriomote Island, hybridization between domestic cats and both the Iriomote cat and Tsushima leopard cat (*Prionailurus bengalensis euptilura*), and interspecific transmission of feline immunodeficiency virus. Together, these findings suggest that free-ranging domestic cats may affect native carnivores through multiple pathways, including competition, genetic introgression, and disease transmission, further broadening their conservation relevance in the East Asian island ecosystems.

Human activity was associated with many of these ecological interactions. Several studies reported free-ranging cats occurring near residential or agricultural areas where food, shelter, or other human-associated resources were available. On Tokunoshima Island, Maeda et al. documented consumption of endangered endemic wildlife by provisioned free-ranging cats, suggesting that access to human-provided food does not necessarily eliminate hunting behavior [34]. However, given the study did not compare provisioned cats with cats lacking access to supplemental feedings, it does not permit conclusions regarding the effect of provisioning on predation intensity. Rather than indicating a direct causal relationship between provisioning and predation pressure, Maeda et al. and others [35,39] suggest that human-associated resources may allow free-ranging cats to persist near sensitive habitats, thereby maintaining opportunities for wildlife interaction.

Other studies similarly associate cat distribution with residential areas, cattle barns, feeding sites, and vacant structures that could provide food or shelter [23,33,50]. Evidence from Amami City further illustrates the complexity of modifying these human-provided resources, where feeding restrictions were associated with shifts in cat abundance and spatial distribution, including increased detection of owned cats within forest interiors [32]. These findings suggest that restricting food access alone may not uniformly reduce opportunities for cat–wildlife interaction and may instead redistribute cat activity. Accordingly, changes in cat movement and spatial distribution should be considered when evaluating such interventions. Collectively, the evidence indicates that provisioning, sheltering, ownership practices, and proximity to human settlements influence cat persistence and opportunities for wildlife interaction.

### 4.4. Sociocultural Context and Management Implications

Public attitudes, ownership practices, and regional governance influence the acceptability, uptake, and sustained implementation of cat-management approaches. Studies from Japan and China generally reported favorable attitudes toward cats, while concerns about free-ranging populations more often centered on sanitation, nuisance, and nighttime noise than on wildlife predation [36,40,41,45,51]. Cat owners also tended to view predation as less problematic and were less supportive of strict confinement than non-owners. Conversely, some studies suggested that greater awareness of ecological effects could increase concern for wildlife conservation and support for management interventions [47,52,53]. Together, these findings indicate that public communication and engagement may influence both cat-supportive behaviors and the acceptability of management strategies.

Management approaches identified in the review included TNR, registration and microchipping, sterilization, restrictions on outdoor access, removal of cats from sensitive habitats, and public-education initiatives. Although TNR was frequently discussed, none of the included studies directly demonstrated that TNR reduced or increased wildlife predation or affected overall wildlife population outcomes. Reported outcomes instead generally focused on cat abundance, colony stability, movement, or program implementation. Accordingly, the available evidence does not support a general conclusion regarding the ecological effectiveness of TNR for wildlife conservation.

Japanese island systems provided the clearest examples of coordinated management. Programs on Kume Island incorporated TNR, rescue, adoption, community participation, and governmental involvement [43], while the northern Okinawa framework combined cat registration, sterilization, responsible ownership, public education, wildlife monitoring, and efforts to prevent cats from entering sensitive forest habitats [44]. These programs illustrate integrated approaches that link cat management with wildlife conservation; however, their ecological effectiveness remains uncertain as wildlife-centered outcomes were not directly evaluated.

This distinction highlights the importance of separating cat-centered endpoints, such as colony size, sterilization rates, or changes in cat movement, from wildlife-centered endpoints, including predation rates, prey abundance, reproductive success, survival, or population recovery. Changes in cat populations alone do not demonstrate changes in wildlife population unless directly measured.

### 4.5. Research Priorities

Several research priorities emerge from this evidence map (Figure 2). First, broader geographic representation is needed. Outside of Japan, only two studies were identified from China, and none were identified from South Korea, Mongolia, Hong Kong, Taiwan, or Far Eastern Russia. Given the unequal database coverage of the present review, this should be interpreted as a gap in the retrieved evidence base from both international and regional databases, rather than definitive evidence that relevant research does not exist in other regions. Future evidence syntheses would benefit from direct access to CNKI, KoreaScience, and other regional databases, as well as international collaborations that can help overcome language and database-access barriers.

Second, greater methodological consistency would improve comparability across studies. Investigators should clearly report cat category, habitat, season, sampling duration and effort, the denominator used for predation estimates, prey-identification methods, and any uncertainties around quantitative estimates. Short-term or seasonal observations should not be extrapolated to annual kill rates unless supported by the study design or a validated model. Molecular approaches, including DNA metabarcoding of scat, may also improve taxonomic resolution and detection of prey that are difficult to identify morphologically.

Third, species-level prey reporting should include conservation status, endemism, and, where available, information on local population size or vulnerability. More importantly, future studies should move beyond diet composition and kill counts and towards Level 3 outcomes, including abundance, survival, recruitment, reproductive success, and population trajectory. Population-viability analysis and other demographic modeling approaches may be particularly useful for determining whether observed mortality is significant enough to influence long-term population persistence.

Finally, management interventions, including TNR, feeding restrictions, confinement, and removal from sensitive habitats, should assess cat-centered and wildlife-centered outcomes alongside implementation fidelity, community participation, and social acceptability. Where feasible, comparison sites or groups, baseline measurements, and repeated follow-up would help distinguish intervention-associated changes from seasonal variation and concurrent environmental influences. Measuring these dimensions together would clarify whether wildlife outcomes improve, worsen, or remain unchanged and how implementation conditions contribute to the observed results.

### 4.6. Strengths and Limitations

This review has several strengths, including a systematic scoping review design, inclusion of Japanese-language databases, explicit separation of consumption, predation-pressure, and population-response evidence, and integration of human-mediated contextual factors with ecological findings. The evidence-classification framework also reduces the risk of interpreting diet or prey-occurrence data as equivalent to demonstrated population-level effects.

Several limitations should nevertheless be considered. Most importantly, the geographic coverage was uneven. Japanese-language literature was searched directly through J-Stage and CiNii Research, whereas planned searches of CNKI and KoreaScience could not be completed due to access limitations, and international databases were searched in English only. Consequently, literature published exclusively in Chinese, Korean, or only in other regional databases may have been underrepresented. The strong concentration of retrieved studies in Japan therefore likely reflects some combination of research activity and differential database accessibility.

Study designs, cat classifications, sampling durations, and reported outcomes were highly heterogeneous, limiting meaningful comparisons or pooling of quantitative predation estimates. Quantitative findings were therefore retained in the units and timeframes reported by the original studies, and no pooled regional kill-rate estimates were generated. Inconsistent species-level prey identification further limited assessment of the taxonomic breadth and conservation significance of documented predation. Moreover, relatively few studies directly evaluated wildlife population responses, constraining conclusions regarding population-level and demographic consequences.

Consistent with the objectives of a scoping review, no formal risk-of-bias or methodological-quality assessment was conducted. Instead, evidence levels were used to characterize the scope of conclusions supported by the measured outcomes rather than ranking study quality. The search strategy was also not formally peer-reviewed using the PRESS framework, which may have influenced search sensitivity. Collectively, these limitations support interpreting this review as a systematic map of the retrieved evidence rather than as a pooled estimate of effect magnitude or a comprehensive assessment of methodological quality.

## 5. Conclusions

The available evidence documents wildlife consumption and predation by free-ranging cats across a diverse wildlife taxon in East Asia, including endemic and threatened species in Japanese island ecosystems. However, relatively few studies directly evaluated wildlife population responses. As such, the evidence more strongly supports the occurrence of cat–wildlife interactions than their demographic consequences.

Human provisioning, sheltering, ownership practices, public attitudes, and governance provide important context for patterns of cat distribution and management. However, observed associations involving these factors do not necessarily demonstrate causal effects on predation. Similarly, changes in cat abundance or the implementation of management interventions alone do not establish corresponding benefits or harms to wildlife populations.

Future research would benefit from broader geographic coverage, greater methodological and reporting consistency, and more direct assessment of wildlife population responses. Evaluations of management interventions should integrate ecological outcomes with measures of implementation effectiveness and social acceptability to determine the conditions under which different approaches achieve their intended objectives. These advances will be necessary to determine when documented cat predation translates into population-level conservation effects and which management approaches effectively reduce those population-level effects in East Asia.

## Figures and Tables

**Figure 1:**
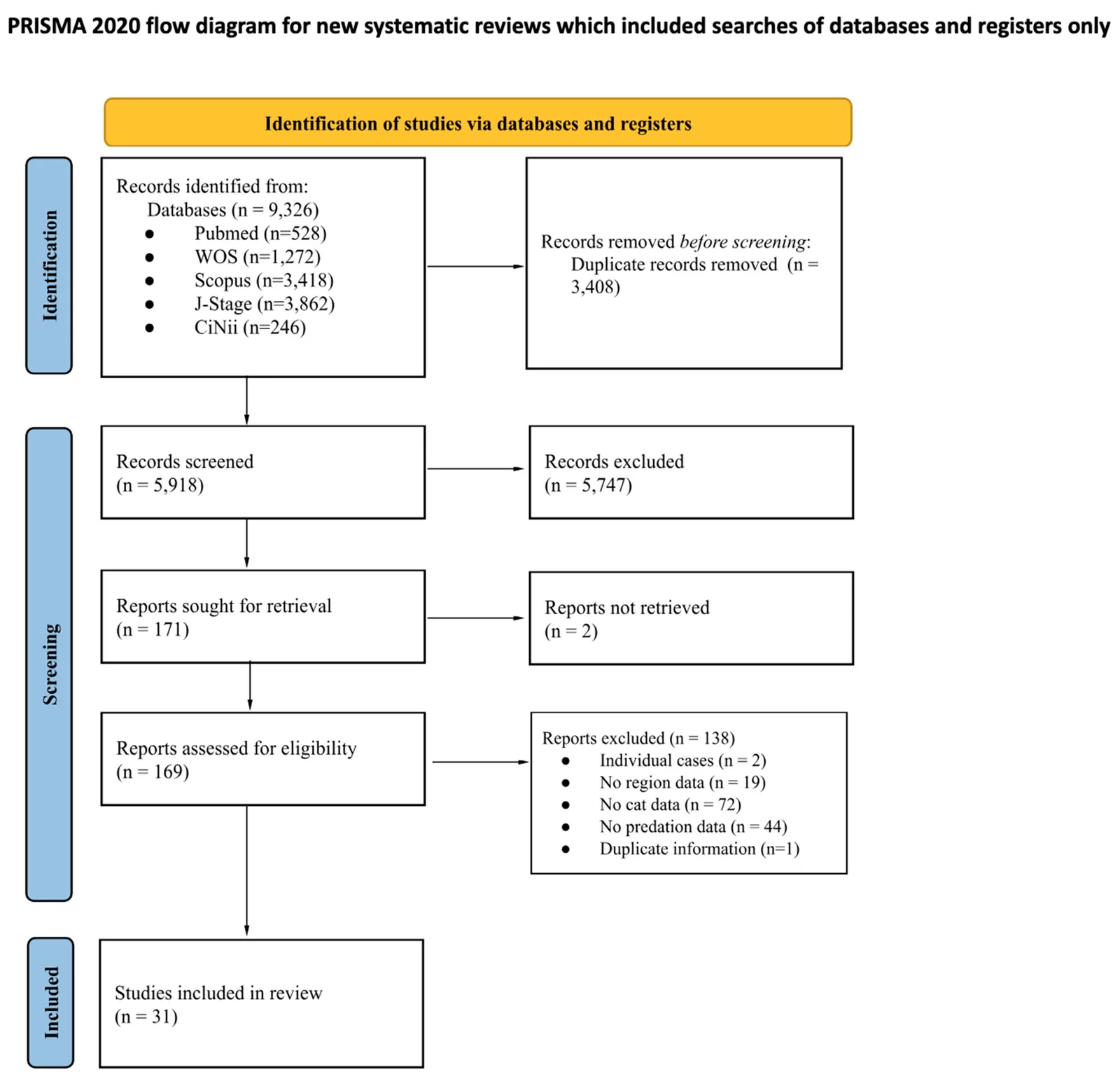
PRISMA Flow Chart.

**Figure 2:**
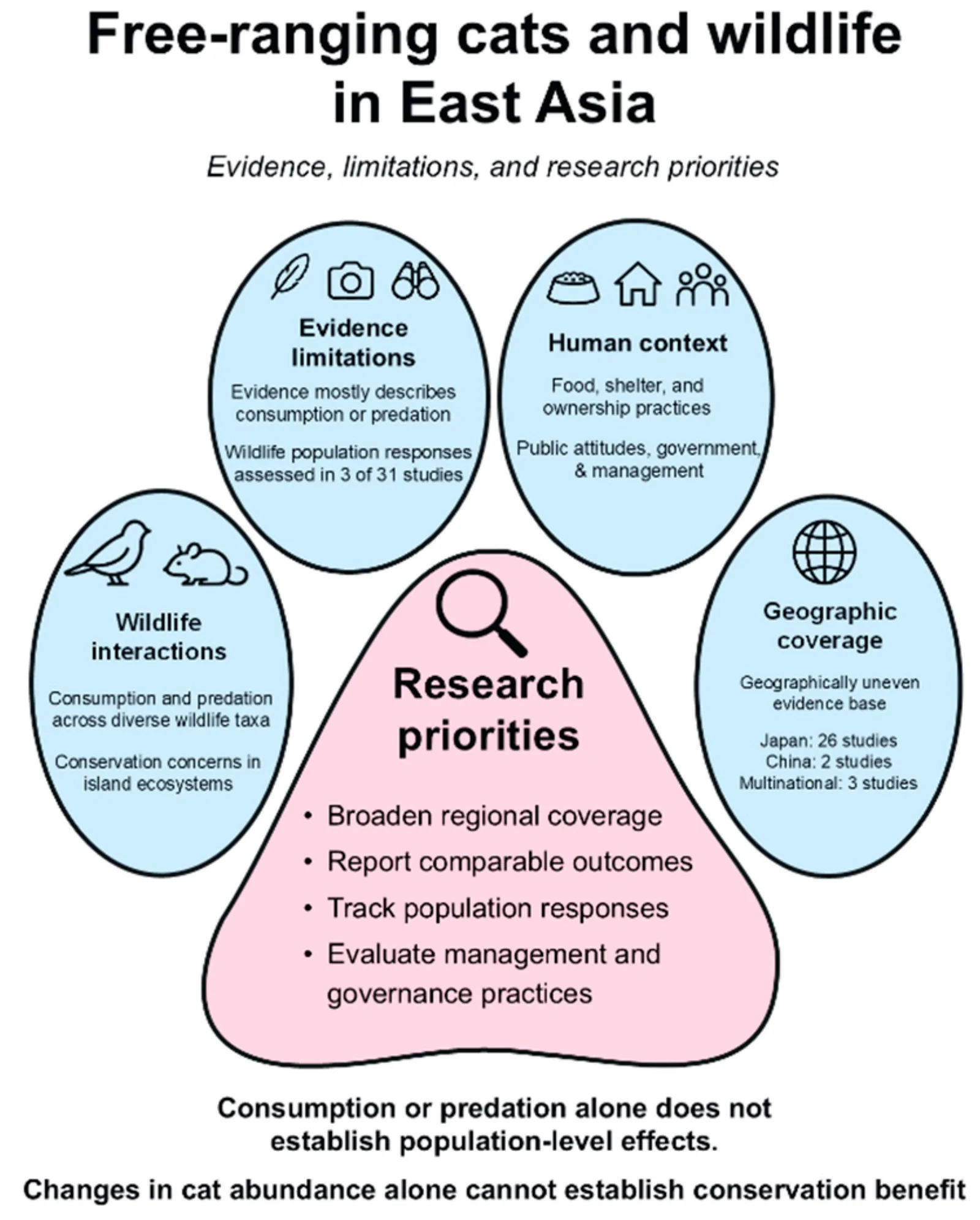
Summary of Evidence.

**Table 1:** Information sources used in the scoping review.

Database	Platform/Interface	Coverage	Last Searched
PubMed	NLM/NCBI	1966–present	June 2026
Web of Science	Clarivate Analytics	1900–present	June 2026
Scopus	Elsevier	1788–present	June 2026
J-Stage	JST (Japan Science and Technology Agency)	1995–present	June 2026
CiNii Research	National Institute of Informatics (Japan)	1990–present	June 2026
Reference list hand-searching	Manual	All included full-texts	June 2026

**Table 2:** PubMed Search Strategy, June 2026.

Search Terms	Filters	Number of Articles
((cat OR “Felis catus” OR “feral cat” OR “stray cat” OR “free-ranging cat”) AND (predation OR predatory OR hunting OR prey OR mortality OR “wildlife impact”) AND (environment OR ecology OR biodiversity OR conservation OR invasive))	English Only	528

**Table 3:** Data items collected from included sources of evidence.

Category	Variable	Definition/Notes
Bibliographic	Author(s), year, source	Publication authors, year, journal, report, or other source
	DOI/identifier	DOI or stable source identifier, where available
Geographic context	Country/region	East Asian study location; global or multinational sources recorded separately when East Asia-specific data were independently extractable
	Ecological setting	Island/mainland and urban, rural, agricultural, or natural setting, as reported
Cat population	Cat category	Owned outdoor, stray/community, feral, mixed, or not specified
	Human association	Ownership, provisioning, shelter, or other documented dependence on human resources
Study design	Method	Scat/stomach analysis, prey return, carcass attribution, direct observation, camera/GPS monitoring, survey, modeling, prey census, management-response study, or mixed method
	Sampling characteristics	Sampling duration/season and number of cats or other relevant denominator, when reported
Evidence classification	Evidence type	Diet/consumption, direct predation, predation-pressure estimate, wildlife population response, or contextual evidence
	Evidence level	Level 1: consumption/diet; Level 2: direct predation/predation pressure; Level 3: wildlife population-level response
Wildlife outcomes	Prey taxon	Species or higher-order taxonomic group identified
	Conservation status	Endemic or threatened status, including IUCN category where reported
	Quantitative outcome	Study-reported prey count, frequency, kill/mortality estimate, biomass, or demographic measure
	Population response	Changes in abundance, survival, recruitment, breeding success, distribution, population viability, or recovery
Quantitative provenance	Value source	Reported directly by the original authors or derived/modelled within the original study; values were retained in their reported units and timeframes
Human context	Provisioning/shelter	Presence and characteristics of human-provided food or shelter
	Management	TNR, removal, confinement, feeding regulation, or other management intervention
	Public/governance context	Ownership practices, public attitudes, legislation, regulation, or other governance factors
Management outcome	Endpoint measured	Cat-centered outcome, wildlife-centered outcome, both, or not reported

**Table 4:** Summary characteristics of included studies by geographic region (n = 31) *.

Characteristic	Japan (n = 26)	China (n = 2)	Global (n = 3)
Island habitat	23	0	Variable
Urban habitat	5	2	Variable
Rural habitat	1	1	Variable
Feral cat focus	20	2	Variable
Fecal/carcass analysis	14	0	Variable
Camera trapping	8	0	Variable
Questionnaire/survey	3	2	Variable

* Methodological categories are not mutually exclusive; individual studies could employ more than one method.

**Table 5:** Reported quantitative estimates of cat predation and prey consumption by study, timeframe, and taxon *.

Publication	Location	Method	Time Frame	Avian	Reptile	Fish	Amphibians	Mammals
Azumi et al. (2021) [22]	Mikura I., JP	Fecal	1 y/cat	340	—	—	—	230
Li et al. (2021) [5]	CN—urban, owned	Survey	1 y/total	65–974 M	52–1275 M	45–1103 M	42–1114 M	319–2928 M
Li et al. (2021) [5]	CN—urban, unowned	Survey	1 y/total	1372–1379 M	727–732 M	826–840 M	638–645 M	1398–1405 M
Li et al. (2021) [5]	CN—rural, owned	Survey	1 y/total	246–2104 M	95–1657 M	57–916 M	104–1677 M	639–4153 M
Li et al. (2021) [5]	CN—rural, unowned	Survey	1 y/total	1006–1061M	602–645 M	683–724 M	351–381 M	1256–1315 M
Nagata et al. (2022) [23]	Mikura I., JP	Carcass	4 d/total	8	—	—	—	—
Oka (2019) [24]	Mikura I., JP	Model	1 y/total	20,000	—	—	—	—
Watari et al. (2025) [25]	Mikura-shima I., JP	CT + fecal	48 d/cat	25.4	—	—	—	57
Tomita et al. [26]	Tobishima I., JP	Carcass	1 d/total	14	—	—	—	—
Kawakami & Fujita (2004) [27]	Hahajima, JP	Carcass	1996, 2001, 2002/total	159	—	—	—	—
Kawakami & Higuchi (2002) [28]	Hahajima, JP	Feather	14 mo/cat	39	—	—	—	—

* Values rounded up to the nearest million. Key: CT = camera traps; d = day(s); I. = island; M = million; mo = months; y = year; CN = China; JP = Japan;/cat = per cat;/total = study total. Em dash (—) denotes non-reported data. Values retain the original study timeframe and basis.

## Data Availability

The data presented in this study are openly available in the Open Science Framework (OSF) repository at [DOI or persistent URL to be added]. The repository contains the final collected dataset, the data collection form used during extraction, and the corresponding variable definitions.

## Supplementary Materials

The following supporting information can be downloaded at: https://www.mdpi.com/article/10.3390/ani16203321/s1; Table S1: Completed PRISMA-ScR checklist; Table S2: Full search strategies for all databases; Table S3: Stage 2 full-text exclusion rationale; Table S4: Scoping Review Articles.

## Abbreviations

OSF	Open Science Framework
UAE	United Arab Emirates
NGO	Non-Governmental Organisation
PRISMA-ScR	Preferred Reporting Items for Systematic reviews and Meta-Analyses extension for Scoping Reviews
UK	United Kingdom
PCC	Population Concept Context
IUCN	International Union for the Protection of Nature
TNR	Trap–Neuter–Release
