# Supplementary material for: Free-Ranging Cat Predation on Wildlife in East Asia: A Scoping Review of Ecological Evidence and Knowledge Gaps
Source: Animals (Basel). 2026 Oct 21;16(20):3321. doi: 10.3390/ani16203321 (PMC13700886; doi:10.3390/ani16203321)
Supplement: Supplementary file 1 [file animals-16-03321-s001.zip › animals-4519614-supplementary.pdf]

Supplementary Table S1: Completed PRISMA-ScR checklist

SECTION	ITEM	PRISMA-ScR CHECKLIST ITEM	REPORTED ON PAGE #
**TITLE**			
Title	1	Identify the report as a scoping review.	1
**ABSTRACT**			
Structured summary	2	Provide a structured summary that includes (as applicable): background, objectives, eligibility criteria, sources of evidence, charting methods, results, and conclusions that relate to the review questions and objectives.	1
**INTRODUCTION**			
Rationale	3	Describe the rationale for the review in the context of what is already known. Explain why the review questions/objectives lend themselves to a scoping review approach.	3
Objectives	4	Provide an explicit statement of the questions and objectives being addressed with reference to their key elements (e.g., population or participants, concepts, and context) or other relevant key elements used to conceptualize the review questions and/or objectives.	4
**METHODS**			
Protocol and registration	5	Indicate whether a review protocol exists; state if and where it can be accessed (e.g., a Web address); and if available, provide registration information, including the registration number.	5
Eligibility criteria	6	Specify characteristics of the sources of evidence used as eligibility criteria (e.g., years considered, language, and publication status), and provide a rationale.	5-6
Information sources*	7	Describe all information sources in the search (e.g., databases with dates of coverage and contact with authors to identify additional sources), as well as the date the most recent search was executed.	6

Search	8	Present the full electronic search strategy for at least 1 database, including any limits used, such that it could be repeated.	6-7
Selection of sources of evidence†	9	State the process for selecting sources of evidence (i.e., screening and eligibility) included in the scoping review.	7-8
Data charting process‡	10	Describe the methods of charting data from the included sources of evidence (e.g., calibrated forms or forms that have been tested by the team before their use, and whether data charting was done independently or in duplicate) and any processes for obtaining and confirming data from investigators.	
Data items	11	List and define all variables for which data were sought and any assumptions and simplifications made.	8
Critical appraisal of individual sources of evidence§	12	If done, provide a rationale for conducting a critical appraisal of included sources of evidence; describe the methods used and how this information was used in any data synthesis (if appropriate).	9
Synthesis of results	13	Describe the methods of handling and summarizing the data that were charted.	10
**RESULTS**			
Selection of sources of evidence	14	Give numbers of sources of evidence screened, assessed for eligibility, and included in the review, with reasons for exclusions at each stage, ideally using a flow diagram.	11-12
Characteristics of sources of evidence	15	For each source of evidence, present characteristics for which data were charted and provide the citations.	19
Critical appraisal within sources of evidence	16	If done, present data on critical appraisal of included sources of evidence (see item 12).	

Results of individual sources of evidence	17	For each included source of evidence, present the relevant data that were charted that relate to the review questions and objectives.	
Synthesis of results	18	Summarize and/or present the charting results as they relate to the review questions and objectives.	20
**DISCUSSION**			
Summary of evidence	19	Summarize the main results (including an overview of concepts, themes, and types of evidence available), link to the review questions and objectives, and consider the relevance to key groups.	24-27
Limitations	20	Discuss the limitations of the scoping review process.	28
Conclusions	21	Provide a general interpretation of the results with respect to the review questions and objectives, as well as potential implications and/or next steps.	28
**FUNDING**			
Funding	22	Describe sources of funding for the included sources of evidence, as well as sources of funding for the scoping review. Describe the role of the funders of the scoping review.	29

* Where sources of evidence (see second footnote) are compiled from, such as bibliographic databases, social media platforms, and Web sites.
† A more inclusive/heterogeneous term used to account for the different types of evidence or data sources (e.g., quantitative and/or qualitative research, expert opinion, and policy documents) that may be eligible in a scoping review as opposed to only studies. This is not to be confused with information sources (see first footnote).
‡ The frameworks by Arksey and O'Malley (6) and Levac and colleagues (7) and the JBI guidance (4, 5) refer to the process of data extraction in a scoping review as data charting.
§ The process of systematically examining research evidence to assess its validity, results, and relevance before using it to inform a decision. This term is used for items 12 and 19 instead of "risk of bias" (which is more applicable to systematic reviews of interventions) to include and acknowledge the various sources of evidence that may be used in a scoping review (e.g., quantitative and/or qualitative research, expert opinion, and policy document).

Supplementary Table S2: Full search strategies for all databases

Database	Search Terms	Filters	Number of Articles
Web of Science	((cat OR "Felis catus") AND (predatory OR predation OR hunting) AND Environment) AND ((cat OR "Felis catus") AND (predatory OR predation OR hunting) AND invasive)	English Only	1272
SCOPUS	((cat OR feline OR "Felis catus" OR felidae) AND (predatory OR predation OR hunting OR prey OR foraging OR predacity OR scavenging) AND environment) AND ((cat OR feline OR "Felis catus" OR felidae) AND (predatory OR predation OR hunting OR prey OR foraging OR predacity OR scavenging) AND invasive)	English only, Articles OR Reviews Only, Included Only with Keywords (Cats, Cat, Felis Catus, Felidae) OR<br>English only, Articles or Reviews Only, Included Only with Keywords (Cats, Cat, Feral Cat, Felid, Felis Catus, Felidae)	3418
J-Stage	(ネコ OR 猫 OR ノネコ OR 野良猫 OR イエネコ OR フェリス・カトゥス) AND (捕食 OR 餌食 OR 食性 OR 被食 OR 生物多様性 OR 種の減少 OR 野生生物への影響 OR 保全) AND (日本 OR 東アジア OR 韓国 OR 中国 OR 台湾 OR 香港)	-	3862
CiNii	(ネコ OR 猫 OR ノネコ OR 野良猫 OR イエネコ OR フェリス・カトゥス OR Felis catus) AND (捕食 OR 餌食 OR 食性 OR 被食 OR 生物多様性 OR 種の減少 OR 野生生物への影響 OR 保全 OR 生態系 OR 在来種) AND (日本 OR 東アジア OR 韓国 OR 中国 OR 台湾 OR 香港 OR アジア)	-	246
Pubmed	((cat OR "Felis catus" OR "feral cat" OR "stray cat" OR "free-ranging cat") AND (predation OR predatory OR hunting OR prey OR mortality OR "wildlife impact") AND (environment OR ecology OR biodiversity OR conservation OR invasive))	English Only	528

Supplementary Table S3: Stage 2 full-text exclusion rationale

Database	ID	Title	Authors	Abstract	Reason
WOS	10.15244/pjoes/169398	Gynecomastia and Risk of Non-Specific Lung Disease: Biochemical and Radiological Relevance	Alghabban, DM; Al-Abbasi, FA; Al-Abbasi, AF; Anwar, F	Lung diseases with increased numbers of cases are reported worldwide with multiple factors including, smoking, lifestyle, and particularly people associated with petrochemical industries. Employees of petrochemical products industries, always at high risk. A single case control 56 years old long-term study from 2014 to final disposal of the case was undertaken, employed in industrial division, exposed to organic fumes, with low albumin, creatinine, elevated serum proteins (91.6 g/L), globulin, CO 2 (39 to 41 mmol/L). His radioallergosorbent test (RAST) positive for dog dander, English plantain, Parientaria officinalis and Dermatophagoides pteronyssinus, cat epithelium and Bermuda grass, Timothy grass, Penicillium notatum and	Cat-related

				Alternaria tenuis, birch and Japanese cedar with high IgE was positive at 547 without eosinophilia. Bilateral gynecomastia, enlarged right paratracheal lymph node (1.2 cm), subcarinal lymph node (1.1 cm), scattered peripheral fine reticular opacities, bilateral traction bronchiectasis with mild patchy ground glass opacity and micronodules. Nonspecific Interstitial lung diseases (NSILD) diagnosed with fall in lung volume (54.6%). 19.3% for upper, 23.9% mid, and 11.4% lower lung zone. Elevated INR (1.2), PT (5.5) and low PTT (30.9) and 7.92 neutrophil, made it classical case of lung transplantation in 2022. Bilateral gynecomastia was over looked that might occurred due exposure of organic fumes of benzene and other chemicals engine oil, smoke of the burning fuel, and hobbies of hunting birds in desert could have cause this disturbance. Hence it	

				might be concluded that in such cases gynecomastia must be the defining factor in diagnosis and treatment before initiating respiratory therapy.	
SCOPUS	15749541	‘I know the tiger by his paw’: A non-invasive footprint identification technique for monitoring individual Amur tigers (Panthera tigris altaica) in snow	Alibhai S.K.; Gu J.; Jewell Z.C.; Morgan J.; Liu D.; Jiang G.	Apex predator populations are in decline around the world. Many exist at low density and are elusive, making the acquisition of reliable data on their numbers and distribution a considerable challenge. The Amur tiger (Panthera tigris altaica) is the largest of the five extant sub-species of tiger. The single most significant, contiguous population, an estimated 550 animals, exists in the Russian Far East, with smaller populations on the far eastern Sino-Russian border. For the last few decades, active efforts on the part of Chinese authorities have encouraged the recolonization of these populations back to their former ranges in Northeast China. Reliable data on Amur tiger numbers and distribution are required to assess	Cat-related

				population recovery at the landscape scale. Footprints, ubiquitous in the snow over range areas, could inform on these baseline data. This paper describes a statistically robust, cost-effective and non-invasive footprint identification technique (FIT) to identify individual tigers from footprints in snow. It is based on a rigorous data collection and data-processing protocol, combined with a cross-validated discriminant analysis method. A Ward's clustering technique provides a visual output of individual classification. The analytical tools are packaged in a user-friendly analytical interface. Between December 2011 and December 2012, we collected a series of 605 footprint images from 44 captive individual Amur tigers for a reference database from which to derive a classification algorithm. The 23 females and 21 males ranged in age from 3 to	

				13 years (female mean age 7.95 +/− 0.18; male mean age 8.08 +/− 0.19). 128 measurements (areas, lengths and angles) were taken from each print and analyzed with the FIT add-in to JMP software. The derived classification algorithm was then applied to 21 footprint trails collected from an unknown number of free-ranging Amur tigers during 2012 and 2015/2016. The algorithm predicted 7 Amur tigers at the site surveyed in 2012, and 4 tigers surveyed at two sites in 2015/16. We demonstrate that the footprint identification technique translates traditional tracking methodologies into a statistically robust and objective analytical tool that can be deployed by both scientists and local communities to monitor the recovery of big cat populations. © 2022	
WOS	10.1163/1568539X-bja10229	Effects of snow leopards (Panthera uncia) on olfactory communication of Pallas's cats (Otocolobus manul) in the Altai Mountains, Mongolia	Allen, ML; Rovero, F; Oberosler, V; Augugliaro, C; Krofel, M	Olfactory communication is important for many solitary carnivores to delineate territories and communicate with potential mates and	Cat-related

				competitors. Pallas's cats (Otocolobus manul) are small felids with little published research on their ecology and behaviour, including if they avoid or change behaviours due to dominant carnivores. We studied their olfactory communication and visitation at scent-marking sites using camera traps in two study areas in Mongolia. We documented four types of olfactory communication behaviours, and olfaction (sniffing) was the most frequent. Pallas's cats used olfactory communication most frequently at sites that were not visited by snow leopards (Panthera uncia) and when they used communal scent-marking sites, they were more likely to use olfactory communication when a longer time had elapsed since the last visit by a snow leopard. This suggests that Pallas's cats may reduce advertising their presence in response to occurrence of snow leopards, possibly to limit predation risk.	

SCOPUS	23766808	Predation of vertebrates by domestic cats in two Brazilian hotspots: incidental records and literature review	Assis, Clodoaldo Lopes (57218896046); Novaes, Camila Moura (56715676700); Dias, Marcelo Augusto Pereira Coelho (58038418400); Guedes, Jhonny José Magalhães (57195603565); Feio, Renato Neves (6506900904); Garbino, Guilherme Siniciato Terra (55241644200)	Domestic cats (Felis catus Linnaeus, 1758) can interact with and cause several negative impacts upon wildlife if unconstrained by their owners. These impacts occur especially because of their innate predatory behavior and, usually the lack of any natural predators, as well as due to their high abundances relative to native carnivores. Although these impacts are well studied in temperate countries, there are but a few records of species used as prey by these carnivores in Brazil. Here, we expand the knowledge of native species preyed upon by domestic cats in Brazil by presenting new records of this predator-prey interaction and reviewing records in the literature. Predation events were recorded through opportunistic encounters between 2016 and 2022. We recorded the predation of 14 native terrestrial vertebrate species (reptiles, amphibians, birds, and mammals) from the	EA

				Cerrado and Atlantic Forest biomes. Our literature review recovered predation events by cats in the two aforementioned biomes plus in the Amazonia. Our results increase to 48 the number of wild species preyed upon by domestic cats in Brazil, which is probably an underestimation, as this number is much higher in other countries of comparable land size and species diversity. We suggest that cat population control measures should be carried out, especially in protected areas. © 2022 The Author(s). Published by Informa UK Limited, trading as Taylor & Francis Group.	
WOS	10.1186/s12983-020-00376-8	Bigger doesn't mean bolder: behavioral variation of four wild rodent species to novelty and predation risk following a fast-slow continuum	Best, IN; Shaner, PJL; Lo, HY; Pei, KJC; Kuo, CC	Background: Understanding how wild species respond to novel situations with associated risk can provide valuable insights for inter-specific behavioral variation and associations with pace-of-life (POL). Rodents, a globally distributed and diverse taxonomic group, have been the subjects of countless studies	Cat-related

				emulating risky situations. Controlled laboratory experiments with a focus on wild-caught species provide the opportunity to test fine-scale behavioral responses to contexts of risk with ecological implications. For example, assessing the importance of predator cues eliciting antipredator responses, as well as whether wild rodents embody behavioral plasticity and repertoires, illustrated by habituation and variation in behavioral traits, respectively. Results: In this comparative study, we examined multiple behavioral responses of four rodent species in eastern Taiwan (three native species Mus caroli, Apodemus agrarius, Rattus losea, and one invasive, Rattus exulans) exposed to an unfamiliar microenvironment and novel cue from an allopatric predator, the leopard cat (Prionailurus bengalensis). All wild-caught animals were subjected to two	

				consecutive nights of experimental trials in a laboratory setting. Behavioral responses to a novel situation during the first trial differed between species; smaller species investing more time in non-defensive behaviors compared to the larger species. More specifically, the smaller species M. caroli and A. agrarius allocated more time to exploration and foraging, whereas the larger rat species R. exulans and R. losea spent more time motionless or concealing. During the second trial, the addition of leopard cat cues did not elicit antipredator behaviors, but rather, rodents were found to exhibit increased non-defensive behaviors, specifically foraging efforts. Conclusions: Our results suggest that these four species do largely follow a behavioral fast-slow continuum with the two smaller mice species demonstrating increased boldness in a novel context compared to the larger rat species. Also,	

				the wild populations of rodents in eastern Taiwan may be naive to leopard cats. Finally, the rodents in our study demonstrated habituation to the microenvironment, indicating they possess adaptive capacity.	
WOS	10.1007/s00265-022-03277-4	Of mice and cats: interspecific variation in prey responses to direct and indirect predator cues	Best, IN; Shaner, PJL; Pei, KJC; Kuo, CC	Prey behavioral responses to predation risk cues may vary between species; moreover, the strength of these behaviors may differ depending on risk cue. In northwestern Taiwan, we used the giving up density (GUD) framework supported with camera trap observations to test how two wild murid rodents that differ by up to fivefold in body size (striped field mouse, Apodemus agrarius, and lesser rice-field rat, Rattus losea) altered their foraging behavior depending on microhabitat characteristics (indirect predator cues) and exposure to predator odors (direct predator cues) of three felids: the native leopard cat (Prionailurus	Predation -related

				bengalensis), the introduced domestic cat (Felis catus), and the exotic bobcat (Lynx rufus). GUD was not affected by predator odors but rather by microhabitat type; rodents removed more seeds under the cover of vegetation compared to exposed food stations, which may reflect a proactive approach to avoiding high-risk areas in a heterogeneous environment. The smaller mouse, A. agrarius, spent more time foraging in experimental food patches compared to the larger rat, R. losea, irrespective of predator odor. Conversely, R. losea spent more time investigating stations and exhibiting vigilance compared to A. agrarius. Species-level differences are consistent with behavioral phenomenon that smaller, faster species confer more boldness compared to larger, slower species, which reinforces the connection between behavior and pace of life, and further elucidates	

				how the behavior of different prey species may not be interchangeable in contexts of risk. Significance statement In the wild, animals eat while trying not to be eaten. Therefore, preys often change their behavior in response to risk cues, but the intricacies of these behavioral shifts can be complex and vary between species. With the use of camera trap monitoring and experimental food patches, we were able to examine fine-scale species-specific behaviors and test for dissimilarities. Two species of wild rodents did not change their foraging behavior to the addition of predator odors, but we did observe an interspecific behavioral variation. The smaller, faster rodent species spent more time foraging, while the larger, slower species spent more time vigilant with more thorough investigation. These interspecific behavioral	

				differences likely indicate the smaller species demonstrated more boldness, whereas the larger rodent was more cautious, which is consistent with the association between pace of life (POL) and behavior.	
SCOPUS	17494877	Spatial density estimate of the snow leopard, Panthera uncia, in the Central Tibetan Plateau, China	Bian, Xiaoxing (57215088435); Liang, Xuchang (57215085709); Weckworth, Byron (8295228500); Jyal, Dorje (57904701900); Hull, Vanessa (24472727900); Yang, Le (57188729804)	Knowledge of large carnivore population abundance is essential for wildlife management and conservation, but these data are often difficult to obtain in inherently low-density species. In particular, the snow leopard, Panthera uncia, an enigmatic cat occupying remote mountains in Central Asia, has received insufficient assessments of its population abundance because of logistical and methodological challenges. Here, we aimed to develop a robust density estimation of snow leopards based on 81 days of camera trapping within a contiguous and previously unsurveyed 1950 km2 area of habitat on the Tibetan Plateau	Cat-related

				(Mayue Township, Shenzha County, Tibet Autonomous Region, China). By applying spatially explicit capture–recapture models, we produced an estimate of 1.40 (95%CI: 1.06–1.84) individuals per 100 km2. Results also suggested sex-specific variation in the range of movement around activity centers, with male (N = 10, σ = 4.02) movement considerably greater than female (N = 8, σ = 1.84) movement. The findings can serve as a reliable baseline reference for assessing the population trends of this endangered felid species with future estimates. This study will provide context to contribute toward a better understanding of ecological factors shaping the distribution and abundance of snow leopards and the related conservation measures needed to sustain their long-term survival on the Tibetan Plateau. © 2022 International Society of Zoological Sciences, Institute of Zoology/Chinese	

				Academy of Sciences and John Wiley & Sons Australia, Ltd.	
SCOPUS	903558	Exploring the ecologic basis for extreme susceptibility of Pallas' cats (Otocolobus manul) to fatal toxoplasmosis	Brown M.; Lappin M.R.; Brown J.L.; Munkhtsog B.; Swanson W.F.	Recent efforts by North American zoos to establish a genetically viable captive population of Pallas' cats (Otocolobus manul) have been compromised by high newborn mortality (~60%), primarily because of toxoplasmosis. The basis for this extreme susceptibility to toxoplasmosis is unknown. In the present study, the general health status of wild Pallas' cats in Mongolia was evaluated, including assessment of basal hematologic parameters and fecal corticoid metabolite concentrations. The prevalence of exposure to Toxoplasma gondii in Mongolian Pallas' cats, local domestic cats, and prey species also was determined based on serology and/or polymerase chain reaction analysis. Biologic samples (blood, feces, and/or brain tissue) were obtained from 15	Cat-related

				wild Pallas' cats, 15 domestic cats, and 45 prey animals (rodents and pikas) captured in Mongolia during the summers of 2000 and 2001. Comparative data were obtained from nine captive Pallas' cats maintained in North American zoos. Based on physical examinations, complete blood counts, and blood chemistry analyses, only minor differences were observed in the general health status of wild and captive Pallas' cats. Fecal cortisol metabolite concentrations did not differ (P>0.05) between populations, indicating that Pallas' cats in captivity and in the wild have similar basal adrenocortical activity. A pronounced difference (P<0.01) in seroprevalence to T. gondii was observed between populations. Whereas all captive Pallas' cats exhibited elevated immunoglobulin titers (IgG>2,048) to T. gondii, only two of 15 (13%) wild Pallas' cats were seropositive, with	

				both cats having lower IgG titers (<1,024). Furthermore, no evidence of exposure to this parasite was found in any of the Mongolian domestic cats or prey species. These findings suggest that wild Palla's cats have minimal opportunity for exposure, to T. gondii in their natural habitat and, typically, do not become infected captive with this parasite, until being brough into captivity. Accordingly, maintenance of a viable captive population may require implementing effective strategies to prevent exposure seropositve females and their highly susceptible offspring. © Wildlife Disease Association 2005.	
WOS	10.1038/s41598-023-39417-7	A dietary perspective of cat-human interactions in two medieval harbors in Iran and Oman revealed through stable isotope analysis	Brozou, A; Fuller, BT; De Cupere, B; Marrast, A; Monchot, H; Peters, J; van de Vijver, K; Lambert, O; Mannino, MA; Ottoni, C; Van Neer, W	Cats are hypercarnivorous, opportunistic animals that have adjusted to anthropogenic environments since the Neolithic period. Through humans, either by direct feeding and/or scavenging on food scraps, the diet of cats	Predation-related

				has been enriched with animals that they cannot kill themselves (e.g., large mammals, fish). Here, we conducted carbon and nitrogen stable isotope ratio analysis to reconstruct the diet of medieval cats and investigate cat-human interactions in two medieval harbor sites (Qalhat, Oman and Siraf, Iran). The analysis included 28 cat individuals and 100 associated marine and terrestrial faunal samples pertaining to > 30 taxa. The isotopic results indicate a high marine protein-based diet for the cats from Qalhat and a mixed marine-terrestrial (C-4) diet for the cats from Siraf. Cats at these sites most likely scavenged on both human food scraps and refuse related to fishing activities, with differences in the two sites most likely associated with the availability of marine resources and/or the living conditions of the cats. By shedding light on the dietary habits of	

				cats from two medieval harbors in the Arabian Gulf and Gulf of Oman, this study illustrates the potential of stable isotope analysis in reconstructing human-cat interactions in the past.	
SCOPUS	20762615	Nipah Virus Disease: Epidemiological, Clinical, Diagnostic and Legislative Aspects of This Unpredictable Emerging Zoonosis	Bruno, Luigi (58045229100); Nappo, Maria Anna (58045322700); Ferrari, Luca (56186227500); Di Lecce, Rosanna (6503861552); Guarnieri, Chiara (58045322800); Cantoni, Anna Maria (7005144904); Corradi, Attilio (7006254970)	Nipah virus (NiV) infection is a viral disease caused by a Henipavirus, belonging to the Paramyxoviridae family, responsible for a zoonosis. The course of the disease can be very serious and lead to death. NiV natural hosts are fruit bats (also known as megabats) belonging to the Pteropodidae family, especially those of the Pteropus genus. Natural infection in domestic animals has been described in farming pigs, horses, domestic and feral dogs and cats. Natural NiV transmission is possible intra-species (pig-to-pig, human-to-human) and inter-species (flying bat-to-human, pig-to-human, horse-to-human). The infection can be spread by humans or animals in different ways. It is peculiar how the viral transmission	Cat-related

				modes among different hosts also change depending on the geographical area for different reasons, including different breeding methods, eating habits and the recently identified genetic traits/molecular features of main virus proteins related to virulence. Outbreaks have been described in Malaysia, Singapore, Bangladesh, India and the Philippines with, in some cases, severe respiratory and neurological disease and high mortality in both humans and pigs. Diagnosis can be made using different methods including serological, molecular, virological and immunohistochemical methods. The cornerstones for control of the disease are biosecurity (via the correct management of reservoir and intermediate/amplifying hosts) and potential vaccines which are still under development. However, the evaluation of the potential influence	

				of climate and anthropogenic changes on the NiV reservoir bats and their habitat as well as on disease spread and inter-specific infections is of great importance. Bats, as natural reservoirs of the virus, are responsible for the viral spread and, therefore, for the outbreaks of the disease in humans and animals. Due to the worldwide distribution of bats, potential new reports and spillovers are not to be dismissed in the future. © 2022 by the authors.	
SCOPUS	0096140X	Aggressiveness in a subtropical shorebird's nest defense is adjusted to the predator species and shared by conspecifics	Brynychová, Kateřina (57207959836); Sládeček, Martin (56152818000); Pešková, Lucie (57247583500); Chajma, Petr (56685771800); Firlová, Veronika (57247583400); Elhassan, Esmat (57204626244); Vozabulová, Eva (57207854482); Janatová, Veronika (57248010600); Almuhery, Aisha (57247795100);	Aggression is an important component of an animal's defense when protecting offspring from predators. Ground nesting birds use a variety of defense strategies. However, their choice according to situation context is poorly known, especially in nonpasserines and in the subtropics and tropics. The ability to distinguish between differently dangerous predator species and the opportunity to share defense with conspecifics are potentially important	EA

			Šálek, Miroslav (7004158099)	but little-studied aspects of nest defense strategy. We experimentally studied the nest defense of Red-Wattled Lapwing in an individually marked population in a desert area near Dubai, UAE. We used three stuffed models representing 1) a predator dangerous both to adults and to nests (a cat), 2) a nest predator (a raven), and 3) a harmless reference model (a moorhen). We confirmed that the lapwings distinguished between predator species (being most aggressive toward the cat, and least aggressive toward the moorhen) and adjusted their defense strategy accordingly. In addition, conspecific visitors play a variety of roles in parents' defense strategy. They can strengthen the parental reaction, or they can assist in distracting a predator. The visitors included not only nesting neighbors but also nonbreeding floaters. Both parents participated in nest defense to a similar extent, regardless of incubation stage and	

				ambient temperature. This study provides new insight into the complexity of the defensive patterns in ground-nesting birds inhabiting a hot environment. Comparative experimental research on a range of environments, with various bird species and predator models, can help us to understand the drivers of these defensive behavioral patterns. © 2022 Wiley Periodicals LLC.	
SCOPUS	19326203	Spatial Co-occurrence and activity patterns of mesocarnivores in the temperate forests of Southwest China	Bu, Hongliang (56921501000); Wang, Fang (56441822500); McShea, William J. (7004690593); Lu, Zhi (19035336300); Wang, Dajun (7407070322); Li, Sheng (56438375200)	Understanding the interactions between species and their coexistence mechanisms will help explain biodiversity maintenance and enable managers to make sound conservation decisions. Mesocarnivores are abundant and diverse mid-sized carnivores and can have profound impacts on the function, structure and dynamics of ecosystem after the extirpation of apex predators in many ecosystems. The moist temperate forests of Southwest China harbor a	Predation-related

				diverse community of mesocarnivores in the absence of apex predators. Sympatric species tend to partition limited resources along time, diet and space to facilitate coexistence. We determined the spatial and temporal patterns for five species of mesocarnivores. We used detection histories from a large camera-trap dataset collected from 2004-2015 with an extensive effort of 23,313 camera-days from 495 camera locations. The five mesocarnivore species included masked palm civet Paguma larvata, leopard cat Prionailurus bengalensis, HOG badger Arctonyx collaris, yellow-throated marten Martes flavigula, and Siberian weasel Mustela sibirica. Only the masked palm civet and HOG badger tended to avoid each other; while for other pairs of species, they occurred independently of each other, or no clear pattern observed. With regard to seasonal activity, yellow-throated marten was most	

				active in winter, opposite the pattern observed for masked palm civet, leopard cat and HOG badger. For diel activity, masked palm civet, leopard cat and HOG badger were primarily nocturnal and crepuscular; yellow-throated marten was diurnal, and Siberian weasel had no clear pattern for most of the year (March to November), but was nocturnal in the winter (December to February). The seasonal shift of the Siberian weasel may be due to the high diet overlap among species in winter. Our results provided new facts and insights into this unique community of mesocarnivores of southwest China, and will facilitate future studies on the mechanism determining coexistence of animal species within complex system.	
SCOPUS	9603115	Factors affecting the occurrence and activity of clouded leopards, common leopards and leopard cats in the Himalayas	Can, Özgün Emre (17433346600); Yadav, Bhupendra Prasad (56985464200); Johnson, Paul J.	Clouded leopards are one of the least known of larger felids and were believed to be extinct in Nepal until 1987. They are particularly	Cat-related

			(55688010600); Ross, Joanna (55604400300); D’Cruze, Neil (12039897300); Macdonald, David W. (7401463172)	interesting because their Asian range spans a diversity of habitats in the fastest disappearing forests in the world and encompasses a guild which differs in composition from place to place. As a part of a wider camera-trapping study of this guild, involving 2948 camera traps at 45 sites in nine countries, and paralleling a similar study of the Sunda clouded leopard including a further 1544 camera traps spanning 22 sites distributed across two countries, we deployed 84 pairs of camera traps for 107 days in 2014 and 2015 at Langtang National Park, Nepal between 1823 and 3824 m a.s.l. within a grid encompassing c. 120 km2. We documented the presence of clouded leopards for the first time at an altitude as high as 3498 m a.s.l. Naïve occupancy for clouded leopard was 8.6% (correcting for detection, 10.1%). Clouded leopards were least active in the middle of the day, and largely crepuscular	

				and nocturnal, as were the common leopards and leopard cats. The peak of clouded leopard activity overlapped with that of musk deer. Prey species for both clouded leopard and common leopard were available across the elevation range studied although the availability of some prey species declined as elevation increased, whereas Himalayan serow, Himalayan goral, and musk deer showed no association with elevation. Before this study, there was no hard evidence that clouded leopards occurred above 2300 m a.s.l., having documented them at almost 4000 m a.s.l. in the Himalayas, we emphasise the importance of this extreme portion of the species' range where climate is likely to change more rapidly and with greater consequences, than the global average. The discovery of clouded leopards in Langtang National Park considerably extends	

				their known range, and raises the possibility that they occur from the Terai in southern Nepal up to the Nepal-Tibet (China) border in the north. Insofar as this study has extended the known extreme boundary of the clouded leopard's geographic range to encompass Langtang National Park in the Nepali Himalayas. © 2019, The Author(s).	
SCOPUS	17494877	Effects of maternal stress induced by predator odors during gestation on behavioral and physiological responses of offspring in Brandt's vole (Lasiopodomys brandtii)	Chen, G.U. (57204846310); Wang, Wenjia (57204847267); Ding, Xiaoqian (57201894298); Yang, Shengmei (15030287200); Wang, Aiqin (55552438700); Yin, Baofa (7101984178); Wei, Wanhong (7403321296)	Maternal effects play a crucial role in regulating populations of small mammals and anti-predator behaviors of offspring. The present study investigated the effects of maternal stress induced by exposure to predator odors during gestation on the behavioral and physiological responses of offspring in Brandt's vole (Lasiopodomys brandtii). Effects included changes in growth, behaviors, hormone levels in serum, and fosB/ΔfosB mRNA and FosB/ΔFosB protein expression in the hypothalamus. Our results showed that when	Predation -related

				pregnant voles were exposed to cat and rabbit urine odors for 18 days (1 h/day), the weight of the female offspring decreased; however, the thymus and ovary indices increased, compared with the control group. Male offspring of voles exposed to cat odor spent more time exploring and foraging, and showed a decrease in corticosterone (CORT) compared with voles in the control group. Both fosB/ΔfosB mRNA and FosB/ΔFosB protein expressions were downregulated in the hypothalamus of male offspring in the cat odor-exposed group compared to that in offspring in the control group. However, the mRNA levels of fosB/ΔfosB in the hypothalamus of female offspring did not decrease in response to cat urine odor. Our findings suggested that maternal stress induced by predator odors during gestation restrained the growth of female offspring and increased the duration of exploring	

				and foraging behaviors of male offspring by regulating the HPA axis, which, in turn, reduced the sensitivity of offspring to unfamiliar environments and increased their probability of becoming prey. © 2018 International Society of Zoological Sciences, Institute of Zoology/Chinese Academy of Sciences and John Wiley & Sons Australia, Ltd	
J-Stage+CiNii		Alien Species Issue in Japan	Chobei IMAI	-	Predation-related
SCOPUS	20762615	Association of Leopard Cat Occurrence with Environmental Factors in Chungnam Province, South Korea	Chung, Ok-Sik (20435620900); Lee, Jong Koo (54179448300)	This study was conducted to investigate the association of leopard cat (Prionailurus bengalensis) occurrences and environmental factors in Chungnam Province, South Korea, using two different analytical approaches for binomial responses: boosted regression trees and logistic regression. The extensive field survey data collected through the Chungnam Biotope Project were used to model	Cat-related

				construction and analysis. Five major influential factors identified by the boosted regression tree analysis were elevation, distance to road, distance to water channel/body, slope and population density. Logistic regression analysis indicated that distance to forest, population density, distance to water, and diameter class of the forest were the significant explanatory variables. The results showed that the leopard cats prefer the areas with higher accessibility of food resources (e.g., abundance and catchability) and avoid the areas adjacent to human-populated areas. The results also implied that boosted regression and logistic regression models could be used in a complementary manner for evaluating wildlife distribution and management. © 2022 by the authors.	
SCOPUS	1476513	Prevalence, genotypes and risk factors for Toxoplasma gondii contamination in marine bivalve shellfish in	Cong, Wei (55241852800); Li, Man-Yao (57216740502); Zou, Yang	We conducted a large-scale epidemiological investigation to detect the prevalence of Toxoplasma gondii in	Cat-related

		offshore waters in eastern China	(57190964215); Ma, Jun-Yang (57220197479); Wang, Bo (57190025333); Jiang, Zhao-Yang (52463806300); Elsheikha, Hany M. (6602154469)	four marine bivalve shellfish species collected from six representative coastal regions of Weihai, eastern China. Between January 2018 and December 2018, 14,535 marine bivalve shellfish pooled into 2907 samples were randomly collected and examined for T. gondii DNA by a nested PCR assay targeting B1 gene. The results showed that 2.8% (82) of the 2907 pooled samples were tested positive for T. gondii DNA. Two T. gondii genotype (ToxoDB Genotype #9 and ToxoDB Genotype #1) were identified PCR-restriction fragment length polymorphism analysis. Factors that were found significantly associated with the presence of T. gondii DNA in marine bivalve shellfish included the source of samples (being wild) (odds ratio [OR], 3.34; 95% confidence interval [CI], 2.00–5.84; $p < 0.01$), surface runoff near the sampling site (OR, 2.64; 95% CI, 1.47–4.72; $p < 0.01$), and	

				presence of cats near the sampling site (OR, 1.77; 95% CI, 1.02–3.07; p = 0.04). Moreover, the prevalence of T. gondii DNA in marine bivalve shellfish correlated with temperature (Pearson's correlation: R = 0.75, p = 0.0049) and precipitation (R = 0.87, p = 0.00021). These findings provide new insights into the presence of T. gondii DNA in marine bivalve shellfish and highlight the impact of human activity on marine pollution by such an important terrestrial pathogen pollutant. © 2021 The Authors	
WOS	10.1016/j.scitotenv.2018.04.293	Behavioral changes in Japanese quails exposed to predicted environmentally relevant abamectin concentrations	de Faria, DBG; Montalvao, MF; Chagas, TQ; Araújo, APC; de Souza, JM; Mendes, BO; Rodrigues, ASL; Malafaia, G	Abamectin (ABA) toxicity in fish, amphibian and mammals was already proven, but its effect on birds is almost unknown. Thus, the aim of our study is to assess the impact of exposure to water with ABA for 40 days at predicted environmentally relevant concentrations on the behavior of female Japanese quails (Coturnix coturnix japonica). The three following	Cat-related

				experimental groups (n= 10 each) were set: control, quails exposed to drinking water, without ABA, EC1x and EC1000x (0.31 mg a. i./L and 310.0 a. i./L, respectively; via commercial formulation Kraft (R) 36EC). The open field test showed anxiolytic response in birds exposed to ABA. These birds did not show locomotor changes or aggressive behavior in the aggressiveness test. Quails exposed to the pesticide did not react to the introduction of an object in the experimental box during the object recognition test, and it suggested perception deficit due to ABA. Moreover, these birds did not recognize the cat (Felix catus) and the vocalization of a hawk (Rupornis magnirostris) as potential predatory threats. These responses also suggest antipredatory behavior deficit due to the pesticide. Thus, our study is pioneer in showing that water with ABA, at tested concentrations,	

				influences the behavior of C. coturnix japonica, as well as in highlighting the potential impacts of this pesticide on this group of birds. (C) 2018 Published by Elsevier B.V.	
SCOPUS	19339747	Cats, foxes and fire: quantitative review reveals that invasive predator activity is most likely to increase shortly after fire	Doherty T.S.; Watchorn D.J.; Miritis V.; Pestell A.J.L.; Geary W.L.	Background: Predators and fire shape ecosystems across the globe and these two forces can interact to impact prey populations. This issue is particularly pertinent in Australia where there is considerable scientific and public interest in the post-fire impacts of two invasive predators—the feral cat and red fox. It remains unclear, though, whether increased cat and fox activity in response to fire is a general phenomenon, or whether the responses are highly context-specific and not generalisable. Results: We reviewed and analysed existing literature and found that a range of positive (e.g., increased activity in burnt areas), negative (decreased activity), and neutral responses have been recorded across	EA

				different studies and locations. Mixed effects modelling revealed that positive responses to fire were more likely when areas were burnt more recently (shorter time since fire). The mean likelihood of increased activity by cats decreased from 41% at 0 months post-fire to 10% at 100 months post-fire, whereas the mean probability for foxes decreased from 53 to 10%. This suggests that there may be a critical time period immediately post-fire when prey are most vulnerable to elevated impacts of predators, and within which management interventions are likely to be most impactful. Conclusions: Many of our findings can be identified as potential cases of either mechanistic or apparent context dependency (variation in recorded patterns due to observational and ecological factors). This provides a pathway for the design of future studies that will enhance	

				our understanding of predator responses to fire, both in Australia and globally. Conservation policy and management will benefit from additional research spanning a greater range of ecosystems and fire events, along with a more comprehensive and nuanced interpretation of existing evidence. © 2023, The Author(s).	
SCOPUS	3044017	All about toxoplasmosis in cats: the last decade	Dubey, J.P. (7201791666); Cerqueira-Cézar, C.K. (56770027000); Murata, F.H.A. (57090154500); Kwok, O.C.H. (7004955825); Yang, Y.R. (56658991300); Su, C. (11439067600)	Toxoplasma gondii infections are common in humans and animals worldwide. Toxoplasmosis continues to be of public health concern. Cats (domestic and wild felids) are the most important host in the epidemiology of toxoplasmosis because they are the only species that can excrete the environmentally resistant oocysts in feces. Cats can excrete millions of oocysts and a single cat can spread infection to many hosts. The present paper summarizes information on prevalence, persistence of infection, clinical signs, and diagnosis of T. gondii infections in	Predation-related

				domestic and wild cats for the past decade. Special emphasis is paid to genetic diversity of T. gondii isolates from cats. Review of literature indicates that a unique genotype (ToxoDB genotype #9 or Chinese 1) is widely prevalent in cats in China and it has been epidemiologically linked to outbreaks of clinical toxoplasmosis in pigs and deaths in humans in China; this genotype has rarely been detected in other countries. This review will be of interest to biologists, parasitologists, veterinarians, and public health workers. © 2020	
SCOPUS	3044017	Toxoplasma gondii infections in dogs: 2009-2020	Dubey, Jitender P. (7201791666); Murata, Fernando H.A. (57090154500); Cerqueira-Cézar, Camila K. (56770027000); Kwok, Oliver C.H. (7004955825); Yang, Yurong (56658991300); Su, Chunlei (11439067600)	Toxoplasma gondii infections are common in humans and animals worldwide. The present review summarizes worldwide information on the prevalence of clinical and subclinical infections, epidemiology, diagnosis, and genetic diversity of T. gondii in dogs (Canis familiaris) from 2009−2020. Seroprevalence estimates of T. gondii worldwide	Cat-related

				were tabulated. Reports of high seroprevalence in canine population and high congenital transmission of T. gondii in dogs in Brazil are reviewed. Most reports from China were published in Chinese, and these reports are now summarized here. Dogs have an additional importance in some countries such as China, Vietnam, and Nigeria; whereas in many cities dog meat is sold commercially for human consumption and given to felids, and transmission of T. gondii could occur if meat is not cooked properly. Dogs can ingest T. gondii-infected cat feces and these oocysts remain viable after passage through the digestive tract of the dog; T. gondii DNA was found in feces of dogs from New York City parks in USA. Most clinical canine cases of toxoplasmosis were in immunosuppressed dogs, and ulcerative dermatitis was one of the main presentations. Genetic diversity based on PCR-	

				RFLP markers using DNA derived from 133 viable T. gondii isolates from dogs from several countries is discussed. T. gondii strains from Asia and Americas were more genetically diverse than those from Africa. This review will be of interest to biologists, parasitologists, veterinarians, and public health workers. © 2020	
SCOPUS	3781135	Coronavirus infection of spotted hyenas in the Serengeti ecosystem	East, Marion L. (7005670430); Moestl, Karin (6603843340); Benetka, Viviane (8092501000); Pitra, Christian (7004259101); Höner, Oliver P. (6602409508); Wachter, Bettina (6701721360); Hofer, Heribert (7101860243)	Sera from 38 free-ranging spotted hyenas (Crocuta crocuta) in the Serengeti ecosystem, Tanzania, were screened for exposure to coronavirus of antigenic group 1. An immunofluorescence assay indicated high levels of exposure to coronavirus among Serengeti hyenas: 95% when considering sera with titer levels of ≥1:10 and 74% when considering sera with titer levels of ≥1:40. Cubs had generally lower mean titer levels than adults. Exposure among Serengeti hyenas to coronavirus was also confirmed by a serum neutralisation assay and	Cat-related

				an ELISA. Application of RT-PCR to 27 fecal samples revealed viral RNA in three samples (11%). All three positive fecal samples were from the 15 juvenile animals (<24 months of age) sampled, and none from the 12 adults sampled. No viral RNA was detected in tissue samples (lymph node, intestine, lung) from 11 individuals. Sequencing of two amplified products from the S protein gene of a positive sample revealed the presence of coronavirus specific RNA with a sequence homology to canine coronavirus of 76 and 78% and to feline coronavirus type II of 80 and 84%, respectively. Estimation of the phylogenetic relationship among coronavirus isolates indicated considerable divergence of the hyena variant from those in European, American and Japanese domestic cats and dogs. From long-term observations of several hundred known individuals, the only	

				clinical sign in hyenas consistent with those described for coronavirus infections in dogs and cats was diarrhea. There was no evidence that coronavirus infection in hyenas caused clinical signs similar to feline infectious peritonitis in domestic cats or was a direct cause of mortality in hyenas. To our knowledge, this is the first report of coronavirus infection in Hyaenidae. © 2004 Elsevier B.V. All rights reserved.	
SCOPUS	15707555	Assessment of potential impact of domestic cats on small mammals in a protected insular area	Ferreira, Giovanne A. (55701902000); Nakano-Oliveira, Eduardo (35299887200); Andriolo, Artur (14028137200); Genaro, Gelson (6603643819)	The presence of domestic cats in natural areas is considered one of the main reasons for species loss, especially on islands and among native small non-volant mammals, birds and reptiles. However, in this study, we detected values opposite to those expected for species richness, abundance, and diversity among different areas when considering the presence or absence and density of these felines. We also observed that in the evaluated environments, prey availability did not	EA

				influence consumption of small mammal species. Our results reinforce the theory of domestic cats' behaviour as being opportunistic and generalist predators. In addition, we only recorded the presence of exotic species in the faeces of cats; that is, we did not capture exotic species in the traps. The close relationship between these exotic species and anthropogenic environments indicates that felines are likely hunting in areas close to the homes where they live. Assessing the impact of predation by domestic cats on native fauna is important for the development and implementation of strategic resource management and to minimize long-term impacts. © 2019 by Koninklijke Brill NV, Leiden, The Netherlands.	
SCOPUS	26319268	Chromosome-level, nanopore-only genome and allele-specific DNA methylation of Pallas's cat, Otocolobus manul	Flack, Nicole (57835214900); Drown, Melissa (57219525228); Walls, Carrie (58070759800);	Pallas's cat, or the manul cat (Otocolobus manul), is a small felid native to the grasslands and steppes of central Asia. Population strongholds in	Cat-related

			Pratte, Jay (58071217000); Mclain, Adam (55346194500); Faulk, Christopher (13613910000)	Mongolia and China face growing challenges from climate change, habitat fragmentation, poaching, and other sources. These threats, combined with O. manul's zoo collection popularity and value in evolutionary biology, necessitate improvement of species genomic resources. We used standalone nanopore sequencing to assemble a 2.5 Gb, 61-contig nuclear assembly and 17097 bp mitogenome for O. manul. The primary nuclear assembly had 56× sequencing coverage, a contig N50 of 118 Mb, and a 94.7% BUSCO completeness score for Carnivora-specific genes. High genome collinearity within Felidae permitted alignment-based scaffolding onto the fishing cat (Prionailurus viverrinus) reference genome. Manul contigs spanned all 19 felid chromosomes with an inferred total gap length of less than 400 kilobases. Modified basecalling and variant phasing produced an	

				alternate pseudohaplotype assembly and allele-specific DNA methylation calls; 61 differentially methylated regions were identified between haplotypes. Nearest features included classical imprinted genes, non-coding RNAs, and putative novel imprinted loci. The assembled mitogenome successfully resolved existing discordance between Felinae nuclear and mtDNA phylogenies. All assembly drafts were generated from 158 Gb of sequence using seven minION flow cells. © 2023 The Author(s). Published by Oxford University Press on behalf of NAR Genomics and Bioinformatics.	
WOS	10.1016/j.biocon.2026.111851	A global synthesis of domestic cats' consumption of invertebrates	Fantle-Lepczyk, Jean E.; Lepczyk, Christopher A.; Dunham, Kylee D.; Rubinoff, Daniel; Bonnaud, Elsa; Woinarski, John C. Z.	Free-ranging domestic cats (Felis catus) are globally distributed invasive carnivores. While recognition of their impacts has focused on consumption of vertebrates, increasing evidence suggests that they also consume large numbers of invertebrate species. Given the	EA

				ongoing concern over invertebrate population declines across the planet, we compiled and analyzed a global database of invertebrate species reported in studies of cat diet. Despite making up >90% of all terrestrial animal species, invertebrates constituted only 7% of the >2000 species we identified as eaten by cats. However, when invertebrates were recorded in cat dietary studies, few were identified to species-level. Four of the 148 invertebrate species we reported to be eaten by cats are considered threatened by the IUCN, but cat predation is not recognized as a threat in their IUCN accounts. IUCN accounts do report cat predation as a threat for 48 invertebrate species of conservation concern, however none of these appear in our database. Insects (especially beetles) constituted ~80% of the 148 invertebrate species reported in cat dietary studies, with crustacean,	

				arachnid, centipede, snail and slug, and millipede species occurring less frequently. Our results add to the growing consensus that cats consume a wide variety of invertebrate species and that they depredate more invertebrate species than is currently recognized. We recommend more cat dietary studies using eDNA (complemented by a more comprehensive eDNA library of invertebrate species), more autecological studies of threatened invertebrate species, and studies of responses of invertebrates to eradication or exclusion of feral cats.	
SCOPUS	8888892	Challenges and opportunities in human dimensions behind cat–wildlife conflict	Fu, Changjian (57248409500); Wang, Fang (56441822500); Zhao, Yumeng (57441836700); Zhu, Qin (57207761378); Luo, Yunchao (57204977863); Li, Yuhang (58293974500); Zhang, Ziye (57748035200);	Because global anthropogenic activities cause vast biodiversity loss, human dimensions research is essential to forming management plans applicable to biodiversity conservation outside wilderness areas. Engaging public participation is crucial in this context to achieve social and environmental benefits. However,	Predation-related

			Yan, Xueting (58937689800); Sun, Taozhu (58679085700); Liu, Yang (57214948771); Li, Zhongqiu (14041900600)	knowledge gaps remain in understanding how a balance between conservation and public demands can be reached and how complicated sociocultural contexts in the Anthropocene can be incorporated in conservation planning. We examined China's nationwide conflict between free-ranging cats (owned cats that are allowed to go outdoors or homeless cats living outdoors) and wildlife to examine how a consensus between compassion and biodiversity conservation can help in decision-making. We surveyed a random sample of people in China online. Over 9000 questionnaires were completed (44.2% response). In aggregate, respondents reported approximately 29 million free-ranging owned cats and that over 5 million domestic cats per year become feral in mainland China. Respondents who were cat owners, female, and religious were more likely to deny the negative impacts of cats on wildlife and ongoing	

				management strategies and more supportive of stray cat shelters, adoption, and community-based fund raising than nonowners, male, and nonreligious respondents ($p < 0.05$). Free-ranging cat ownership and abandonment occurred less with owners with more knowledge of biodiversity and invasive species than with respondents with less knowledge of these subjects ($p < 0.05$). We recommend that cat enthusiasts and wildlife conservationists participate in community-based initiatives, such as campaigns to keep cats indoors. Our study provides a substantially useful framework for other regions where free-ranging cats are undergoing rapid expansion. © 2024 The Authors. Conservation Biology published by Wiley Periodicals LLC on behalf of Society for Conservation Biology.	
WOS	10.1002/jwmg.22150	Spatio-temporal occurrence and sensitivity to livestock	Greco, I; Oberosler, V;	Biased research and conservation efforts	Cat-related

		husbandry of Pallas's cat in the Mongolian Altai	Monti, IE; Augugliaro, C; Barashkova, A; Rovero, F	result in some faunal groups (e.g., small felids) being understudied, and hence these groups are often declining without adequate knowledge to manage for threat reduction. The Pallas's cat (Otocolobus manul) occurs across central and western Asia with declining populations and the largest population is likely in Mongolia. A potential threat to this felid is livestock encroachment across its range, including within protected areas, yet we lack a clear understanding of the impact of livestock husbandry on this cat. We used motion-sensitive camera data from 216 sites in 4 study areas in western Mongolia to study the occurrence probability of Pallas's cat in relation to habitat characteristics and occurrence of livestock, and conducted a local assessment within a strictly protected area where we obtained the highest number of detections. We estimated a relatively low	

				occupancy (0.33 +/- 0.10), which is associated with sites with natural vegetation, steeper slopes, and greater prey abundance. Occupancy also increased with increasing livestock occurrence, particularly large herds of sheep and goats. Such co-occurrence was partially adjusted by diel activity segregation, presumably to limit direct encounters. Our results suggest that the preferred habitat by Pallas's cat in the study region coincides with areas encroached by livestock. The Pallas's cat's habitat is specialized and its dependence on areas that are increasingly used for grazing may eventually threaten the cat with habitat degradation, prey depletion, predation by dogs, and poisoning from pest control. Relevant conservation actions should regulate livestock encroachment within protected areas and improve grazing regimes. The Pallas's cat is an indicator species of mountainous and steppe	

				ecosystems in central Asia; hence, further research towards the preservation of its populations would also benefit other key species across its range.	
SCOPUS	251461	A rangewide distribution model for the Pallas's cat (Otocolobus manul): Identifying potential new survey regions for an understudied small cat	Greenspan, Evan (57201998817); Giordano, Anthony J. (59033331900)	Knowledge about the current distribution of threatened and/or understudied species is a fundamental component of conservation biology. Mapping species distributions based on recent known occurrences is particularly important for those that are rare or declining. Too often, cryptic species go undetected throughout parts of their range, whereas others just receive less research attention. We used contemporary presence data for the Pallas's cat (Otocolobus manul), a small cryptic felid, to characterize potential rangewide and regional habitat for the species and identify those abiotic and biotic variables most influencing its distribution. Several regions lacking contemporary occurrence	Cat-related

				records contain potential habitat for Pallas's cats, including the Koh-i-Baba Mountains of Afghanistan, Qinghai-Tibetan Plateau, steppes of Inner Mongolia, Kunlun Mountains of China, and Tian Shan and Pamir Mountains of Kyrgyzstan, Tajikistan, and China. Some of these areas have not been included in prior rangewide distribution assessments. The distribution of pikas (Ochotona spp.), small mammals that likely represent a critical prey species everywhere they are sympatric, was the most important factor affecting the Pallas's cat's distribution. This suggests Pallas's cats may be prey specialists, and that pika presence and habitat are critical considerations for future Pallas's cat surveys and in the development of regional conservation actions. © 2021 Walter de Gruyter GmbH, Berlin/Boston.	
SCOPUS	20762615	A Survey of Public Opinion on Community Cats' General Health and Relationship	Gu, Xuan (57054601700); Zhang, Zilin	The management and coexistence of community cats in urban	Predation -related

		Quality with Residents in Urban China	(57224579446); Peng, Guo (57224576237); Ni, Anru (24502959400); Wang, Bo (57266081100); Xiong, Xiufan (58879764800); Liu, Yujie (58879923000); Wang, Li (58879449300)	areas is a growing concern amid global urbanization. Through a survey-based investigation, we examine the residents' perceptions of the general health of community cats and human-cat relationships in urban China. The data from 5382 participants revealed that approximately 70% of participants perceived community cats as being in good health, and 60% reported harmonious or non-conflict coexistence between residents and these cats. Around 45% of the participants rescued or helped community cats, 38% expressed their intention to adopt, and 18% complained about the issues of community cats to management staff. Linear, logistic, and multilevel-logistic regressions were employed to examine the associations between the types of cities and communities or the participants' socio-demographics and the perceived well-being of	

				community cats or human-cat relationships. The results show that the cats in fourth-tier cities (e.g., county-level cities) had poorer living conditions than in first-tier cities (e.g., Beijing), while the cats in urban village communities (e.g., villages in the city) were less likely to exhibit good health than in ordinary commercial housing communities. The results also show that socio-demographic variables, such as educational attainment, marital status, and income level, predicted participants' relationships with community cats. This study is the first of its kind. It provides valuable insights for stakeholders to develop effective policies and interventions on cat management, emphasizing the need for tailored strategies in diverse urban settings and populations. © 2024 by the authors.	
WOS	10.1093/jmammal/gyac044	Dietary diversity and niche partitioning of carnivores across the Qinghai-Tibetan	Hacker, CE; Cong, W; Xue, YD; Li, J; Zhang, Y; Wu, LJ; Ji, YR; Dai, YC;	Understanding predator guild resource use is vital for carnivore species preservation and	Cat-related

		Plateau of China using DNA metabarcoding	Li, Y; Jin, LX; Li, DQ; Zhang, JY; Janecka, JE; Zhang, YG	ecosystem function in high-altitude landscapes. We describe the dietary composition, similarity, and niche overlap for seven carnivore species across three regions of the Qinghai-Tibetan Plateau (Qilian Shan, East Burhanbuda Mountain, Yushu) using DNA metabarcoding of 760 scat samples. Analyses found 33 unique prey items representative of the classes Mammalia, Aves, and Actinopterygii. Blue sheep (Pseudois nayaur) and pika (Ochotona sp.) were most frequently detected. Livestock in carnivore diets ranged from 4% to 7% depending on site, but accounted for 21.7% of Tibetan wolf (Canis lupus) diet. Eurasian lynx (Lynx lynx), Tibetan fox (Vulpes ferrilata), Pallas's cat (Otocolobus manul), and snow leopard (Panthera uncia) had highly uneven diets. Intraguild predation of mesocarnivores by apex carnivores was found. Analysis of dietary niche overlap using Pianka's	

				index showed that overlap was significantly greater than expected among all carnivores examined and between apex and mesocarnivores, though significant correlations between carnivore body mass and prey body mass suggested broad separation in consumed prey. Snow leopard and Tibetan wolf diets were consistently the most similar of any two given species pairs. This study provides evidence of high dietary overlap. Protection for species consumed by a wide variety of carnivores will benefit predator guilds in the area and assist in maintaining the relatively low frequency of livestock predation. This work provides insight into the dynamics of carnivore guilds living in an area that has worldwide impacts, and methods herein could be applied on global scales for conservation efforts.	
SCOPUS	19326203	Community attitudes and practices of urban residents regarding predation by pet	Hall, Catherine M. (56510899500); Adams, Nigel A. (57213716653);	International differences in practices and attitudes regarding pet cats' interactions with wildlife	Predation -related

		cats on wildlife: An International comparison	Bradley, J. Stuart (57202528413); Bryant, Kate A. (7005904844); Davis, Alisa A. (57188828846); Dickman, Christopher R. (7102521104); Fujita, Tsumugi (57188830756); Kobayashi, Shinichi (57188828549); Lepczyk, Christopher A. (6602210762); McBride, E. Anne (55626545700); Pollock, Kenneth H. (7006354430); Styles, Irene M. (6603265855); Van Heezik, Yolanda (7003411007); Wang, Ferian (57188833837); Calver, Michael C. (56450505500)	were assessed by surveying citizens from at least two cities in Australia, New Zealand, the UK, the USA, China and Japan. Predictions tested were: (i) cat owners would agree less than non-cat owners that cats might threaten wildlife, (ii) cat owners value wildlife less than non-cat owners, (iii) cat owners are less accepting of cat legislation/restrictions than non-owners, and (iv) respondents from regions with high endemic biodiversity (Australia, New Zealand, China and the USA state of Hawaii) would be most concerned about pet cats threatening wildlife. Everywhere non-owners were more likely than owners to agree that pet cats killing wildlife were a problem in cities, towns and rural areas. Agreement amongst nonowners was highest in Australia (95%) and New Zealand (78%) and lowest in the UK (38%). Irrespective of ownership, over 85% of respondents from all countries except China	

				(65%) valued wildlife in cities, towns and rural areas. Non-owners advocated cat legislation more strongly than owners except in Japan. Australian non-owners were the most supportive (88%), followed by Chinese non-owners (80%) and Japanese owners (79.5%). The UK was least supportive (non-owners 43%, owners 25%). Many Australian (62%), New Zealand (51%) and Chinese owners (42%) agreed that pet cats killing wildlife in cities, towns and rural areas was a problem, while Hawaiian owners were similar to the mainland USA (20%). Thus high endemic biodiversity might contribute to attitudes in some, but not all, countries. Husbandry practices varied internationally, with predation highest where fewer cats were confined. Although the risk of wildlife population declines caused by pet cats justifies precautionary action, campaigns based on	

				wildlife protection are unlikely to succeed outside Australia or New Zealand. Restrictions on roaming protect wildlife and benefit cat welfare, so welfare is a better rationale. © 2016 Hall et al. This is an open access article distributed under the terms of the Creative Commons Attribution License, which permits unrestricted use, distribution, and reproduction in any medium, provided the original author and source are credited.	
SCOPUS	9632719	Approaches to conserving vulnerable wildlife in China: Does the colour of cat matter - If it catches mice?	Harris, Richard B. (7405695609)	China's environmental problems are well known, but recently its record in the area of wildlife conservation, particularly with regard to endangered species, has come under scrutiny. Environmental values colour how we in the West view both China's past experience with wildlife and what strategies it should adopt to foster better conservation. Chinese have long taken a utilitarian view of wildlife, valuing species primarily as resources for	Predation -related

				man's use and only secondarily for other reasons. However, China has not developed institutions capable of sustaining the desired use of wildlife in the face of ever-growing demands. I suggest that Western criticisms of Chinese utilitarian attitudes are inappropriate, ineffective, and possibly counter-productive: deep-seated cultural mores change slowly. Instead, Westerners concerned with the fate of China's wildlife should assist the development of systems that act to channel demand for wildlife's material benefits toward investment in conservation. Such systems will likely require devolution of considerable control to local levels. strengthening incentives to favour long-over short-term benefits, and - notwithstanding common Western attitudes - substantial consumptive use of wildlife.	
WOS	10.1002/ecs2.4338	Marbled cats in Southeast Asia: Are diurnal and semi-	Hendry, A; Amir, Z; Decoeur, H; Mendes, CP;	Southeast Asia supports the greatest diversity of felids globally, but this	Cat-related

		arboreal felids at greater risk from human disturbances?	Moore, JH; Sovie, A; Luskin, MS	diversity is threatened by the severe forest loss and degradation occurring in the region. The response of felids to disturbances appears to differ depending on their ecology. For example, the largely terrestrial and nocturnal leopard cat (Prionailurus bengalensis) thrives near forest edges and in oil palm plantations where it hunts rodents (Muridae) at night, thereby avoiding human activity peaks. Conversely, we hypothesized that the sympatric and similar-sized marbled cat (Pardofelis marmorata) would respond negatively to edges and relatively open oil palm plantations as they are more arboreal than leopard cats, rely on tree connectivity for hunting, and are diurnal so have less potential to temporally avoid humans. We used camera trapping from Southeast Asia to test habitat associations at multiple spatial scales using zero-inflated Poisson generalized linear mixed models and hierarchical	

				occupancy modeling. We found that marbled cats were positively associated with large intact forests and, in contrast to leopard cats, negatively associated with oil palm plantations. Furthermore, we found preliminary evidence suggesting marbled cats may adapt their diel activity to become more crepuscular in degraded forests, likely shifting their activity to avoid humans. These findings suggest that the marbled cat's International Union for Conservation of Nature (IUCN) Red List conservation status should potentially be upgraded from Near Threatened to Vulnerable, matching other forest-dependent felids in the region. We posit our findings may be generalizable such that semi-arboreal and diurnal felids could face greater threats from habitat degradation than their terrestrial and nocturnal relatives.	
SCOPUS	1474919X	The catastrophic impact of invasive mammalian predators on birds of the UK	Hilton G.M.; Cuthbert R.J.	The UK has sovereignty over 16 Overseas Territories, which hold	EA

		Overseas Territories: A review and synthesis		some of the world's great seabird colonies and collectively support more endemic and globally threatened bird species than the whole of mainland Europe. Invasive alien mammalian predators have spread throughout most of the Territories, primarily since European expansion in the 16th century. Here we review and synthesize the scale of their impacts, historical and current, actions to reduce and reverse these impacts, and priorities for conservation. Mammalian predators have caused a catastrophic wave of extinctions and reductions in seabird colony size that mark the UKOTs as a major centre of global extinction. Mammal-induced declines of threatened endemics and seabird colonies continue, with four Critically Endangered endemics on Gough Island (Tristan da Cunha), St Helena and Montserrat directly threatened by invasive	

				alien House Mice Mus musculus, Feral Cats Felis catus and rats Rattus spp. Action to reduce these threats and restore islands has been modest in comparison with other developed countries, although some notable successes have occurred and a large number of ambitious eradication and conservation plans are in preparation. Priority islands for conservation action against mammalian predators include Gough (which according to one published prioritization scheme is the highest-ranked island in the world for mammal eradication), St Helena and Montserrat, but also on Tristan da Cunha, Pitcairn and the Falkland Islands. Technical, financial and political will is required to push forward and fund the eradication of invasive mammalian predators on these islands, which would significantly reduce extinction risk for a number of globally threatened species. ©	

				2010 The Authors. Journal compilation © 2010 British Ornithologists' Union.	
SCOPUS	3949370	Adaptations to prey base in the hypercarnivorous leopard cat Prionailurus bengalensis	Hisano, Masumi (56667248800); Newman, Chris (35550720700)	Investigating biogeographical variations in diet composition can help understand the adaptability and generalism of species. Although the dietary adaptability of omnivorous mesocarnivores is well established, far less work has explored how more specialist hypercarnivores optimise their diets. By reviewing 11 studies of the leopard cat (Prionailurus bengalensis), we quantitatively examined how dietary composition varies over the wide range of biomes they occupy in Asia. Specifically, we contrasted the diet of the Iriomote Island sub-species (south-western Japan), where native rodents are absent, with that of the mainland. Leopard cat diet typically comprised mammals, birds, amphibians, reptiles, and	Cat-related

				invertebrates. In Iriomote Island, however, the low relative frequency of occurrence of small mammals (only introduced rats) was compensated by higher frequencies of reptiles and amphibians compared to the mainland. Consequently, trophic diversity and dietary niche breadth were higher for leopard cats in Iriomote Island than for the mainland. This shows that even hypercarnivorous species can use trophic plasticity to adapt to local prey availability. Given that rodent numbers often fluctuate substantially over time, the availability of alternative prey, such as herptiles, may be vital for the conservation of the leopard cat, and especially the critically endangered Iriomote cat. More generally, the trophic versatility of hypercarnivores must be considered when assessing their vulnerability to environmental change. © 2020, © 2020 Dipartimento di Biologia,	

				Università di Firenze, Italia.	
SCOPUS	10353712	Pre-eradication assessment of feral cat density and population size across Kangaroo Island, South Australia	Hohnen, Rosemary (55857687300); Berris, Karleah (57205720876); Hodgens, Pat (57207261063); Mulvaney, Josh (57188864618); Florence, Brenton (57219775158); Murphy, Brett P. (15056810000); Legge, Sarah M. (6701640976); Dickman, Chris R. (7102521104); Woinarski, John C.Z. (57203255851)	Context: Feral cats (Felis catus) are a significant threat to wildlife in Australia and globally. In Australia, densities of feral cats vary across the continent and also between the mainland and offshore islands. Densities on small islands may be at least an order of magnitude higher than those in adjacent mainland areas. To provide cat-free havens for biodiversity, cat-control and eradication programs are increasingly occurring on Australian offshore islands. However, planning such eradications is difficult, particularly on large islands where cat densities could vary considerably. Aims: In the present study, we examined how feral cat densities vary among three habitats on Kangaroo Island, a large Australian offshore island for which feral cat eradication is planned. Methods: Densities were compared among the	EA

				following three broad habitat types: forest, forest-farmland boundaries and farmland. To detect cats, three remote-camera arrays were deployed in each habitat type, and density around each array was calculated using a spatially explicit capture-recapture framework. Key results: The average feral cat density on Kangaroo Island (0.37 cats km-2) was slightly higher than that on the Australian mainland. Densities varied from 0.06 to 3.27 cats km-2 and were inconsistent within broad habitat types. Densities were highest on farms that had a high availability of macropod and sheep carcasses. The relationship between cat density and the proportion of cleared land in the surrounding area was weak. The total feral cat population of Kangaroo Island was estimated at 1629 ± 661 (mean ± s.e.) individuals. Conclusions: Cat densities on Kangaroo Island are highly variable	

				and may be locally affected by factors such as prey and carrion availability. Implications: For cat eradication to be successful, resources must be sufficient to control at least the average cat density (0.37 cats km-2), with additional effort around areas of high carcass availability (where cats are likely to be at a higher density) potentially also being required. © 2020 CSIRO.	
SCOPUS	10.1080/10888705.2024.2424579	The Impact of Human-Animal Interaction on Animal Welfare and Owners' Job Performance: A Study on Cats, Dogs and Birds	Çelebi F.; Karabulut C.	Human-Animal Interaction (HAI) has been significant since ancient times, initially with animals, especially dogs, assisting in hunting and food gathering. Over time, this relationship evolved into domestication, companionship, and therapeutic roles in society. In urban areas, pet ownership is widespread, driven by companionship, therapeutic benefits, and social status. This study focuses on urban pet owners due to structured work environments, allowing a clear	EA

				examination of the relationship between HAI, animal welfare, and work performance. The study explores HAI's dual impact on animal welfare and the work performance of dog, cat, and bird owners. Key factors include psychological, social, and physical closeness in HAI; animal hygiene, physical activity, and loneliness for welfare; and work performance for owners. Data were collected from 404 pet-owning employees in Istanbul, with statistical analyses confirming tool validity. Results reveal a significant positive correlation (r = 0.568) between HAI and animal welfare, and a positive but smaller correlation (r = 0.215) between HAI and work performance. Findings support HAI's role in enhancing well-being, suggesting pet-friendly policies in workplaces to maximize benefits. © 2024 Informa UK Limited, trading as Taylor & Francis Group.	

WOS	10.1111/1440-1703.70085	Temporal Compensation Among Different Non-Native Prey Sustains the Food Supply of an Intraguild Predator Threatening a Native Species	Tokuyoshi, Mikuni; Nakashita, Rumiko; Nose, Tsugumi; Hayama, Hisayo; Oka, Nariko; Watari, Yuya; Miyashita, Tadashi	Intraguild predation (IGP) is common in nature, and understanding the temporal changes in IGP structure is important for ecosystem management and biodiversity conservation. However, partial IGP, where the apex predator consumes not only shared prey and the mesopredator (IG prey) but also other prey (alternative prey), has rarely been addressed in terms of its temporal structural dynamics. We used stable isotope analysis to investigate seasonal changes in the structure of partial IGP involving feral cats, two non-native rat species (Rattus norvegicus and Rattus rattus), and seabirds on Mikura-shima Island, Japan. We also estimated the temporal dynamics of these two rat species by conducting a trapping survey. The results showed that cats depended on seabirds (shared prey) as food when seabirds were present, but when seabirds were absent,	Unable to access data

				shifted their diet to Norway rats and subsequently showed increased reliance on black rats. In addition, Norway rats appeared to depend more on seabirds as food than black rats. Furthermore, the catch per unit effort of Norway rats declined sharply from the first to the second half of the period when seabirds were absent. These results suggested that Norway rats are IG prey, whereas black rats tend to be an alternative prey with less dependence on shared prey. The seasonal decline in Norway rats (IG prey) may have shifted this system from IGP to partial IGP, increasing the dependence on alternative prey. This study highlights the importance of understanding the dynamics of partial IGP involving multiple non-native predators for their management.	
WOS	10.1016/j.biocon.2026.111829	Introduced species are a global threat to lizard biodiversit	Stevens, Nicole; Fitzgerald, Lee	Invasive species are a major cause of vertebrate extinctions worldwide, yet there is limited	EA

				research on their impacts to global patterns of extinction and endangerment in reptiles. We assessed the relative impact of invasive species to extinction risk in native lizards, identified major invasive genera and threat types (e.g., mammalian predator), and examined geographic and ecological correlates of invasive-driven extinction risk using IUCN Red List data. We found that invasive species are the fourth most recorded threat to lizard species yet included the largest number of extinct lizard species of any threat type (N = 15). An additional 27 species are possibly extinct, indicating that this impact may be substantially greater. At least 128 invasive genera impact native lizards including plants, mammals, reptiles, birds, amphibians, and invertebrates through predation, competition, habitat disruption and/or hybridization. Invasive mammalian predators,	

				especially cats and rats, were the most common threats and associated with most extinctions. Islands were significantly more likely to contain threatened or extinct lizard species, with pronounced hotspots in the Caribbean and Oceania. Deforestation and mammalian habitat disruptors were correlated with significantly higher threat categories for invasive-impacted lizards, while anthropogenically modified landscapes and threats from reptile species were associated with lower extinction risk. Our results place research on invasive-native interactions on lizards into a global context to better understand large-scale patterns. Invasive species currently function as the largest driver of lizard extinction, so management should focus on targeted prevention and control in at-risk hotspots to mitigate this significant threat to global biodiversity.	

WOS	10.1007/s10336-015-1250-1	Endemic Amami Jay, invasive Small Indian Mongoose, and other alien organisms: a new century investigation of island aliens towards improved ecosystem management	Ishida, K; Murata, K; Nishiumi, I; Takahashi, Y; Takashi, M	The Amami Islands in southern Japan host many endemic species, including Amami Jay (Garrulus lidthi). Following the introduction of Small Indian Mongoose (Herpestes auropunctatus) in 1979, populations of Amami Rabbit (Pentalagus furnessi), Amami Woodcock (Scolopax mira), Amami Ishikawa's Frog (Odorrana splendida), and other endemic species declined in the 1990s, as the mongoose expanded its population. A mongoose control project since 2000 successfully reduced the mongoose population to a very low density by 2014, resulting in the initial recovery of severely affected animal populations. Amami Jay was removed from Japan's endangered species list in 2008. However the population and range of Amami Jay are still small (ca. 700 pairs, sometimes with other family member(s), in 700 km 2), and other	Cat-related

				invasive species, such as cats and black rats, continue to damage the native ecosystem. Additionally there is a constant threat of new invasions to the Amami Islands. Our research shows that Amami Jay breeding success depends on the dominant oak (Castanopsis sieboldii) acorn production as much as predation. Therefore conservation plans should consider both short-and long-term effects and potential interaction by both invasive species and other natural factors. Understanding the Amami Jay population dynamics and sustainability from the multiple view points of, such as, population ecology, disease ecology, genetics, and also with ex situ conservation efforts as they relate to invasive species demands, will sophisticate our ecosystem research and management. We discuss how this endeavor of ours might develop as a model activity for maintaining biodiversity	

				under threat from multiple invasive species.	
SCOPUS	63207	Ecology and conservation of two endangered subspecies of the leopard cat (Prionailurus bengalensis) on Japanese islands	Izawa, Masako (7101781352); Doi, Teruo (7402078944); Nakanishi, Nozomi (7202974472); Teranishi, Ayumi (26636109200)	The conservation of rare species on islands is a special challenge, especially when the islands are faced with increasing human impact. Two endangered wild felids in Japan provide a contrast that is useful for examining conservation strategies. The Iriomote cat (Prionailurus bengalensis iriomotensis) is endemic to Iriomotejima Island in Okinawa, and the Tsushima leopard cat (Prionailurus bengalensis euptilurus) occurs in the Tsushima islands in Japan, although a related subspecies found in the Korean Peninsula. Population size was approximately 100 individuals for each subspecies. While the population of Iriomote cat appears relatively stable, the population and abundance of the Tsushima leopard cat has decreased in recent decades. Because of their small population sizes and restricted habitats both are listed as	Cat-related

				endangered species/subspecies in Japan's Red List, and the Iriomote cat is listed as an endangered subspecies in the IUCN Red List. Although both are similar-sized felids living on small islands, their ecological characteristics such as food habit, habitat selection, and density differ. These differences seem to be caused by the climate, the biological environment (the species composition of fauna in each island, and the presence of competitors), and the artificial background. The threats facing these two felids are also similar. Habitat destruction, traffic accidents, and negative influences by introduced species are the principal threats to be addressed for their conservation. At the same time, there are differences in the degree of impact by each threat, in each species' conservation programs in practice, and in the stages of progress and operation of programs. These differences are partly	

				because they have different ecological features and partly because the social background of each island, such as human population, main industry, and historical relationship between human and wildlife, are quite different. We will compare the ecology and state of these two wild felids and discuss the different conservational situations in relation to felid ecology and human society. © 2009 Elsevier Ltd. All rights reserved.	
WOS	10.1016/j.biocon.2017.05.018	Land sharing and land sparing reveal social and ecological synergy in big cat conservation	Jiang, GS; Wang, GM; Holyoak, M; Yu, Q; Jia, XB; Guan, Y; Bao, H; Hua, Y; Zhang, MH; Ma, JZ	Global biodiversity conservation has recently focused on the roles of land management strategies of land sharing vs. land sparing. However, few studies have evaluated the roles of social and ecological interactions in modifying the effectiveness of land management for top predator conservation. Using a 65-year dataset from northeastern China, we evaluated the roles of government social policies in resolving human-wildlife conflicts and improving human	Cat-related

				livelihood. From 1998 to 2015, both big cat populations and their habitats have increased. Concurrently, regional human population density decreased by 59.6%, forest volume logged was reduced by 62.6%. Consequently, increases of key prey species were observed during the same periods. Although populations remained small, the annual finite rate of increase was 1.04 for the Amur tiger population and 1.08 for Amur leopards from 1999 to 2015. Habitat areas occupied by big cats increased significantly. Overexploitation of forest resources and big cat declines under previous unsustainable land use are progressively being reversed under land sparing. Large economic investment and intense human-relocation projects coupled with efforts to reduce poaching and illegal hunting and trapping demonstrate a complex social and ecological	

				synergy in big cat conservation in China.	
WOS	10.1007/978-4-431-53859-2_2	Impacts of Invasive Alien Species on Native Ecosystems on the Bonin Islands	Kachi, N	This part introduces quantitative studies on the impacts of invasive alien animals and plants on native ecosystems on the Bonin Islands. Predation by alien predatory flatworms such as Platydemus manokwari has caused the rapid decline or extinction of many endemic land snails. In addition, predation by black rats can cause micro-evolutionary changes in shell morphology and habitat use of endemic land snails. Feral cats prey upon land birds as well as seabirds characteristic to the Bonin Islands. An introduced lizard, the green anole (Anolis carolinensis), has caused serious damage to many insect populations, resulting in changes in the pollination systems of many native plants. Grazing and trampling by feral goats has caused destruction of vegetation, leading to accelerated soil erosion on some islands. An alien tree,	Ind.

				Bischofia javanica, has replaced native trees due to its greater survival and seedling growth than those of native tree species. Invasion and dominance of an exotic shrub, Leucaena leucocephala, have prevented the establishment of some native woody species. Scientific understanding of the impacts of invasive species and their interactions with native species is essential for conservation and restoration of the native Bonin Island ecosystems.	
J-Stage+CiNii		Islands, birds, mammals and humans	Kawakami and Kotaka	-	Predation-related
J-Stage+CiNii		Feral cat predation on seabirds on Hahajima, the Bonin Islands, Southern Japan	Kazuto KAWAKAMI and Masaki FUJITA	The Bonin Islands are oceanic islands that have never been connected with the Japanese mainland, as such they remained free of terrestrial mammalian carnivores until human settlement began in 1830. Shortly thereafter, however, the domestic cat Felis catus was introduced to the islands by settlers and became feral (Obana 1877). The	Ind.

				number of feral cats has increased to the point where they are now considered to have an adverse impact on the native wild birds (Kawakami 2002; Kawakami & Higuchi 2002). On Hahajima, one of the islands of the Bonin group, there are now considered to be more than 100 feral or half-feral cats ranging throughout the island (Kawakami & Higuchi 2002). Kawakami (2000) has already reported that many native birds, especially seabirds, are preyed upon by feral cats, and has speculated that seabirds are frequently targeted because they can not move quickly once they have landed.<br>In the Minamizaki area of Hahajima, many dead seabirds were found to show signs of being killed by feral cats. Since there is no prior information on such bird mortality from the islands, we report here on species composition, and on which parts of birds most frequently remain after predation. Since	

				predated individuals often decompose and some parts are lost, it is not easy to assess the number of individuals involved. We propose, therefore, a convenient method for estimating the minimal number.	
J-Stage+CiNii		The Declining Distribution of the Okinawa Rail Gallirallus okinawae	Kiyoaki Ozaki, Takao Baba, Shigemoto Komeda, Michio Kinjyo, Yutaka Toguchi and Tetsujiro Harato	The distribution of the Okinawa Rail Gallirallus okinawae was studied, using the voice play back method, at 296 sites in 1996-1999 and at 564 sites in 2000-2001, in the northern part of Okinawa Island. The results were plotted on a 1.25km×0.925km map grid. The presence of Okinawa Rails was confirmed at 49 out of 95 grid intersections (51.6%) during 1996-1999 and at 116 out of 255 grid intersections (45.5%) in 2000-2001. These results were compared with similar research undertaken by the Environmental Agency in 1985-1986. This comparison indicates that the border of the species' range has shifted north by about 10km over the past 15 years, resulting in a 25%	Predation-related

				decrease in the range of this species. Between 2 October 2000 and 30 March 2001 Okinawa Prefectural Government carried out controls of the introduced mongoose Herpestes javanicus in northern Okinawa Island using approximately 800 traps located at a total of 2,470 sites. A total of 303 mongoose were caught, mainly in the southern area from which Okinawa Rails have disappeared in recent years. The result of rail population mapping and of mongoose trapping strongly imply that the mongoose has caused the range contraction of the Okinawa Rail. We believe that urgent conservation measures are required in order to prevent this species from becoming extinct.	
SCOPUS	14712148	A voyage to Terra Australis: Human-mediated dispersal of cats	Koch, K. (56268544100); Algar, D. (6505797006); Searle, J.B. (7102976955); Pfenninger, M. (6603948584); Schwenk, K. (7004572911)	Background: Cats have been transported as human commensals worldwide giving rise to many feral populations. In Australia, feral cats have caused decline and extinction of native mammals, but their time of introduction and origin	Predation-related

				is unclear. Here, we investigate hypotheses of cat arrival pre- or post-European settlement, and the potential for admixture between cats of different invasion events. We analyse the genetic structure and diversity of feral cats from six locations on mainland Australia, seven Australian islands and samples from Southeast Asia and Europe using microsatellite and mitochondrial DNA data. Results: Our results based on phylogeographic model selection are consistent with a European origin of cats in Australia. We find genetic distinctiveness of Australian mainland samples compared with Dirk Hartog Island, Flinders Island, Tasman Island and Cocos (Keeling) Island samples, and genetic similarities between some of the island populations. Historical records suggest that introduction of cats to these islands occurred at the time of European exploration and/or in connection with	

				the pearling, whaling and sealing trades early in the 19th century. Ongoing influx of domestic cats into the feral cat population is apparently causing the Australian mainland populations to be genetically differentiated from those island populations, which likely are remnants of the historically introduced cat genotypes. Conclusion: A mainly European origin of feral cats in Australia, with possible secondary introductions from Asia following the initial establishment of cats in Australia is reasonable. The islands surrounding Australia may represent founding populations and are of particular interest. The results of the study provide an important timeframe for the impact of feral cats on native species in Australia. © 2015 Koch et al.	
J-Stage+CiNii		Literature Survey on Predators of Snakes in Japan	Koji Tanaka and Akira Mori	-	Predation-related
SCOPUS	14697998	Predation by domestic cats in rural areas of central Poland:	Krauze-Gryz, D. (8656384300); Gryz, J.	Studies of cat trophic behaviour can be based on collections of the prey	EA

		An assessment based on two methods	(8656384200); Goszczyński, J. (6603799406)	brought home or the prey eaten by cats (i.e. analyses of scat/gut contents). Both methods involve biases with respect to palatability, prey size and assessment of hunting rates. Furthermore, these methods are often used on different groups of cats (i.e. house-based vs. feral), thus results are difficult to compare. In the present study, cats from the same area (rural areas in central Poland) were studied by both methods: prey brought home and prey eaten (scat and gut analyses). Both methods identified mammals as the most frequent prey (followed by birds). However, differences occurred in the percentages of the four main vertebrate groups brought home versus eaten by cats: reptiles tended to be brought home, whereas amphibians tended to be eaten. No such difference was found for birds and mammals. Second, the relative proportions of presumably more palatable and presumably	

				less palatable prey differed. The relative proportions of mice and voles (the latter eaten more frequently) and the relative proportions of soricomorphs and rodents (the latter eaten more frequently) were different. Finally, small prey items (i.e. invertebrates) were recorded incompletely for the brought-home method. Overall, the prey-brought-home method underrepresented small prey and underestimated the predation rate for cats, whereas the prey-eaten method was less likely to record unpalatable prey. We thus recommend to combine these two methods to obtain fuller and truer assessment of cat predation. © 2012 The Zoological Society of London.	
SCOPUS	3051838	Carnivores (Mammalia: Carnivora) in South China: A status review with notes on the commercial trade	Lau, Michael Wai-Neng (16238996000); Fellowes, John R. (18834965700); Chan, Bosco Pui Lok (36851816800)	Evidence from the literature, interviews, market surveys, general rapid field surveys and camera trapping was reviewed to infer the regional status of 33 recorded carnivores in South China (Guangxi,	Cat-related

				Guangdong, Hainan, Hong Kong and Macau). The carnivore fauna in South China is among the most depleted for any continental area in the world. The tiger Panthera tigris, leopard Panthera pardus, grey wolf Canis lupus and binturong Arctictis binturong are probably extirpated. The dhole Cuon alpinus, Asiatic golden cat Catopuma temmincki and clouded leopard Neofelis nebulosa are at great risk of regional extirpation, as are the more ecologically adaptable red fox Vulpes vulpes, raccoon dog Nyctereutes procyonoides, Asian badger Meles leucurus, Eurasian otter Lutra lutra, Oriental small-clawed otter Aonyx cinereus and large Indian civet Viverra zibetha. Though more widely reported, the Asiatic black bear Ursus thibetanus, yellow-throated marten Martes flavigula, hog badger Arctonyx collaris and Siberian weasel Mustela sibirica are at regional risk. The status of the Burmese ferret-badger	

				Melogale personata, stripe-backed weasel Mustela strigidorsa, smooth-coated otter Lutrogale perspicillata, large-spotted civet Viverra megaspila and Owston's palm civet Chrotogale owstoni is uncertain due to inadequate information. Five carnivore species - the Chinese ferret-badger Melogale moschata, masked palm civet Paguma larvata, yellow-bellied weasel Mustela kathiah, small Indian civet Viverricula indica and leopard cat Prionailurus bengalensis - are relatively secure (though not common) in the region, along with the Asian palm civet Paradoxurus hermaphroditus and the Javan mongoose Herpestes javanicus in some areas, while the spotted linsang Prionodon pardicolor and crab-eating mongoose Herpestes urva may have been under-reported. The main threats have been habitat destruction and unsustainable exploitation. Only in	

				Hong Kong, where enforcement of protection legislation has been stronger, are the surviving carnivore species easily encountered. Improved enforcement and monitoring are essential to retaining the remainder of South China's carnivores. Support could be boosted by conservation education and better research. © 2010 The Authors. Journal compilation © 2010 Mammal Society.	
SCOPUS	63207	Understanding how free-ranging cats interact with humans: A case study in China with management implications	Li Y.; Wan Y.; Zhang Y.; Gong Z.; Li Z.	The growing population of outdoor free-ranging cats poses increasing threats to biodiversity. While those threats are now well recognized, how human-cat interactions contribute to shape population dynamics have been overlooked. In this study, we explore major variables associated with the distribution of free-ranging cat density in 30 universities in Nanjing, Jiangsu Province, China. We specifically focus on possible even greater care devoted by women to the free-ranging cats. We	Predation-related

				found that, as expected, the density of feeding stations is positively associated to the density of free-ranging cats. More interestingly, the density of male students versus female students seemed to be non-randomly associated with the distribution of cats among universities. An online questionnaire confirmed that women were more concerned about the living conditions of free-ranging cats than men in China. Finally, a socialization test focusing on 27 free-ranging cats conducted by female and male observers suggests that cats may have the ability to adopt a friendlier behavior with female students. Our result suggests that human-cat relationships can be understood using multiple angles, including population dynamics, behavioral ecology and conservation psychology. Such a better understanding of human-cat interactions is necessary to develop	

				relevant population management in urban context. © 2020 Elsevier Ltd	
WOS	10.1016/j.gecco.2021.e01899	What factors relate with the activity synchronization intensity among big cats and their ungulate prey in Northeast China?	Liu, SY; Qi, JZ; Gu, JY; Long, ZX; Ma, JZ; Jiang, GS	The activity synchronization rhythm is an important aspect of measuring interspecific interactions between predators and prey, and crucial for large carnivores conservation. However, little is known about what factors relate their activity synchronization dynamics. With the climate change and human society development, the spatial-temporal distribution and activity synchronized rhythm of large carnivores and their prey would inevitably be affected, which would further affect the adaptability of large carnivores and bring new challenges to their conservation. In this study, we hypothesized the ambient temperature and human activity would influence the activity synchronization intensity among Amur tiger, Amur leopard and ungulates (roe deer, sika	Cat-related

				deer and wild boar) through cross-correlation function and temporal niche overlap model based on the camera traps data. The result show that the activity rhythms of the five target species showed species and seasonal differences, the temporal niche overlaps between ungulates were normally higher than that between the cats and prey, the weakest overlap was observed between the two big cat species, and time-lags of similar rhythmic activity can reduce the temporal overlap pressures of species. Furthermore, we found that ambient temperature relate interspecific synchrony of roe deer-sika deer and roe deer-wild boar pairs at the daily scale. And at hourly scale, temperature and human activities exerted significantly positive or negative stresses on the interspecific activity synchronization of all target species, observed in all three seasons. Our study provides new insights on our	

				understanding of the mechanisms by which sympatric predators and ungulate prey coexist and interact in view of temporal niche overlap, as well as how wildlife managers can manage the mammal assembly effectively. As the Amur tiger and leopard populations in China are being recovered quickly, the rapidly increasing predation pressure and limited environmental resources could have profound implications for the interaction and coexistence among these two large carnivores and their ungulate prey. Managers should strengthen the monitoring, and understand its causes and consequences, so as to develop effective protective measures.	
WOS	10.1016/j.quascirev.2023.108420	Diversification of faunal exploitation strategy and human-climate interaction in Southern China and Southeast Asia during the last deglaciation	Lu, YX; Gao, F; Wang, YR; Ma, MM; Zhou, AF; Liu, RL; Kikuchi, H; Ren, LL; Shao, QF; Chen, L; Xu, YX; Li, HR; Dong, GH	Southern China and Southeast Asia were favourable habitats for foragers during the Last Glacial Maximum (similar to 25-18 ka BP) and the Last Deglaciation (similar to 18-11.7 ka BP), despite various climate fluctuation.	Cat-related

				However, the underlying subsistence strategies in these areas remains unclear, due to the lack of systematic chronological and archaeological data. The current study reports new results of radiocarbon dating, U-series dating, and zooarchaeological analysis from the Naminan cave site, located at the boundary between Southern China and Southeast Asia. It reveals that deer was consumed as the primary source of meat (91.7 %), supplemented by turtles, wild oxen, monkeys, wild boars, and mustelids during similar to 17.35-16.49 ka BP. The subsequent period of similar to 15.55-15.35 ka BP saw a radical declining proportion of deer, with various new species being consumed, such as shrews, porcupines, fish, crab, canidae, and wild cat. Human occupation of the Naminan cave site might have lasted until the Early Holocene. Comparing archaeological and	

				paleoclimate evidence from Southern China and Southeast Asia, it is reasonable to argue that a progressively drier and colder climate during similar to 17.35-15.35 ka BP appeared to trigger a remarkable decline in human activity but greater range of diets in locations such as the Naminan cave and Yuchanyan sites, facilitating further expansion of hunting-gathering groups during the late phase of the Last Deglaciation with warm and humid climate. This study contributes fresh data and perspective to a greater understanding of the evolution of human-climate interactions in low-latitude regions during Late Paleolithic period.	
SCOPUS	207519	Problems of epidemiology and prophylaxis of alveococcosis (multilocular echinococcosis): A general review-with particular reference to the U.S.S.R.	Lukashenko, N.P. (6603349130)	Lukashenko N. P., 1971. Problems of epidemiology and prophylaxis of alveococcosis (multilocular echinococcosis): a general review-with particular reference to the U.S.S.R. International Journal for Parasitology,	Cat-related

				1: 125-134. Alveococcus multilocularis is an extremely dangerous, often fatal, parasite of man. The main endemic areas are southern G.F.R., Austria, Switzerland, northern U.S.A., Canada and Japan. The circulation of Alveococcus depends on complex biocenotic relationships between certain carnivores and numerous microtine rodents. Their roles vary widely according to terrain, reproduction, season, epizootics, animals of prey and interspecific rivalry. The infection rate of definitive hosts each year depends on the prevailing numbers of intermediate hosts in the corresponding biotope, and vice versa. The significance of the fox, polar fox, dog fox, wolf and spotted cat as definitive hosts is considered. Twenty-nine species of rodents have been recorded as intermediate hosts but the roles of insectivores appear insignificant, while those of birds and	

				wild ungulates have yet to be studied. Domestic ungulates probably do not take part in the life-cycle of A. multilocularis. Domestic cats and dogs may be involved accidentally. The role of synanthropic rodents has not yet been fully elucidated but house mice show a high degree of infectivity. Human infection is influenced by ecological factors, living conditions, occupation and level of hygiene practised: dangerous sources of infection are team dogs, unboiled drinking water from melted ice, the skins of fur animals and possibly insects. Secondary sources are other contaminated waters, dust, wild berries and possibly vegetables. Data on the incidence of alveococcosis according to sex is contradictory; the differences between the infection rates of men and women in different regions almost certainly depend on their several modes of life and occupations. The majority of diseased	

				persons are between 19 and 40 years old; infection probably takes place during childhood and is fatal before old age. Prophylactic measures differ markedly from those for (unilocular) echinococcosis, and must be directed towards eliminating the possibility of infecting definitive hosts and towards increased hygiene education. © 1971.	
SCOPUS	9747893	Camera trap survey of mammals in cleopatra's needle critical habitat in Puerto Princesa City, Palawan, Philippines	Marler P.N.; Calago S.; Ragon M.; Castro L.S.G.	A camera trap survey was conducted in the recently protected Cleopatra's Needle Critical Habitat (CNCH) in Puerto Princesa City, Palawan, Philippines from February to May 2015 at 39 camera trap sites. A bait of common pig's blood was used at 36 sites, while the three remaining sites were surveyed without a bait and monitored a stream with a latrine site or mud bath with tracks. Seven native species were detected and three of these species were endemic to the island province. Species	Cat-related

				included: Common Palm Civet Paradoxurus philippinensis, Palawan Porcupine Hystrix pumila, Collared Mongoose Urva semitorquata, Palawan Stink Badger Mydaus marchei, Palawan Leopard Cat Prionailurus bengalensis heaneyi, Asian Small-clawed Otter Aonyx cinereus, and Malay Civet Viverra tangalunga. Analysis of the activity patterns of the three most commonly captured species revealed predominantly nocturnal activity for the Common Palm Civet, Palawan Porcupine, and Palawan Stink Badger. The Philippine Palm Civet showed occasional diurnal activity. The seven photo-captured species appeared most common, or were at the least recorded, below 750m. Five species (the Philippine Palm Civet, Palawan Porcupine, Collared Mongoose, Palawan Stink Badger, and Palawan Leopard Cat) were also recorded above 1000m. The CNCH supports two	

				threatened species, the Palawan Porcupine and the Asian Small-clawed Otter, which are listed as Vulnerable by the IUCN, and the Collared Mongoose is listed as Near Threatened. The Palawan Leopard Cat is considered Vulnerable within the Philippines, although it has yet to be assessed by the IUCN. This documentation highlights the biodiversity significance within the newly protected critical habitat and the need to support ongoing conservation efforts within the critical habitat. © Marler et al. 2019.	
J-Stage+CiNii		Status of Black-tailed Gull Larus crassirostris breeding along the coast of northern Niigata	Masaki SHIRAI and Takahiro ARA	-	Predation-related
SCOPUS	15729710	A review on the impacts of feral cats (Felis silvestris catus) in the Canary Islands: Implications for the conservation of its endangered fauna	Medina, Félix Manuel (7101954537); Nogales, Manuel (7003555503)	Feral cats have been directly responsible for the extinction of numerous species on islands worldwide, including endemic species of mammals, birds and reptiles. The diet of feral cats in the main habitats of the Canary Islands, as	EA

				generally occurred on oceanic islands, is mainly composed of introduced mammals, and native species of birds, reptiles and insects. The impact of feral cat upon the endangered species was assessed by evaluating their relative abundance in the cats' diet and by considering their current conservation status. A total of 68 different preys were identified at species level in all studies carried out in the Canary Islands (5 mammals, 16 birds, 15 reptiles and 32 invertebrates). From all the species preyed by feral cats in the Canary Islands, only four of them are considered threatened by the IUCN Red List of Threatened Species: one endemic bird Saxicola dacotiae and three endemic giant lizards, Gallotia simonyi, Gallotia intermedia, and Gallotia gomerana. Although some efforts on management control have been carried out, it is necessary to enforce these conservation activities on those areas of Tenerife, La Gomera	

				and El Hierro where giant lizards are still present. Furthermore some local areas where endangered bird species are highly predated should be protected. Nevertheless, it is important to take into account the presence of other introduced species such as rats, mice or rabbits in order to avoid problems derived from the hyperpredation process and mesopredator release effect. © 2008 Springer Science+Business Media B.V.	
WOS	10.1016/S0168-0102(01)00240-1	Organization of the neurons of origin of the descending pathways from the ferret superior colliculus	Meredith, MA; Miller, LK; Ramoa, AS; Clemo, HRH; Behan, M	The superior colliculus (SC), through its descending projections to the brainstem and spinal cord, is involved in initiating sensory-driven orienting behaviors. Ferrets are carnivores that hunt both above and below ground using visual (and auditory) cues in the daylight but non-visual cues in darkness and in subterranean environments. The present investigation sought to determine whether the ferret SC shows organizational	Cat-related

				features similar to those found in other visually dominant animals (e.g. cats), or whether characteristics of colliculi from non-visually dominant animals (e.g. rodents) prevail. Injection of retrograde tracer into the identified targets of the colliculus (cervical spinal cord, the contralateral pontomedullary reticular formation, or the ipsilateral pontine reticular formation) labeled tectospinal, crossed tectoreticular, and ipsilateral tectoreticular neurons, respectively, within the adult ferret SC. Labeled tectospinal and crossed tectoreticular neurons were far outnumbered by neurons with ipsilateral reticular projections. Like those of their visually dominant relatives, ferret tectospinal neurons were well represented throughout the anterior-posterior extent of the SC and crossed tectoreticular neurons tended to be distributed more broadly across the intermediate gray layer than those of	

				rodents. Thus, even though ferrets perform well as subterranean predators where non-visual cues initiate orienting behaviors, these anatomical characteristics indicate that their colliculi are organized similar to that of their visually dominant, carnivorous relatives. (C) 2001 Elsevier Science Ireland Ltd and the Japan Neuroscience Society. All rights reserved.	
SCOPUS	15451542	Density and habitat use of the leopard cat (Prionailurus bengalensis) in three commercial forest reserves in Sabah, Malaysian Borneo	Mohamed, Azlan (56666657200); Sollmann, Rahel (25961045700); Bernard, Henry (7103326502); Ambu, Laurentius N. (8201660100); Lagan, Peter (16304265100); Mannan, Sam (16304987300); Hofer, Heribert (7101860243); Wilting, Andreas (15133557500)	The small (2- to 7-kg) leopard cat (Prionailurus bengalensis) is the most common cat species in Asia. Although it occurs in a wide range of habitats and seems to adapt well to anthropogenic habitat changes, surprisingly little is known about this species in the wild. All studies have focused on protected areas, although a large proportion of Southeast Asian forests are timber concessions. During this study, we used large camera-trapping data sets (783 records of 124 individuals) from 3 commercially used	Cat-related

				forests to investigate consequences of different logging regimes on density and habitat associations of the leopard cat. We applied spatial capture-recapture models accounting for the location of camera-traps (on or off road) to obtain estimates of leopard cat density. Density was higher in the 2 more disturbed forest reserves ($\bar{X}$ = 12.4 individuals/100 km2 ± 1.6 SE and 16.5 ± 2 individuals/100 km2) than in the sustainably managed forest (9.6 ± 1.7 individuals/100 km2). Encounter rates with off-road traps were only 3.6-9.1% of those for on-road traps. Occupancy models, which accounted for spatial autocorrelation between sampling sites by using a conditional autoregressive model, revealed that canopy closure and ratio of climax to pioneer trees had a significantly negative impact on leopard cat occurrence. Our results confirm that the leopard cat is doing well in modified	

				landscapes and even seems to benefit from the opening of forests. With such flexibility the leopard cat is an exception among tropical rain-forest carnivores. © 2013 American Society of Mammalogists.	
SCOPUS	8888892	Illegal trade in wild cats and its link to Chinese-led development in Central and South America	Morcatty, Thais Q. (55939284000); Bausch Macedo, Jonathan C. (57217390853); Nekaris, K. Anne-Isola (6603467721); Ni, Qingyong (46661207200); Durigan, Carlos C. (57192182565); Svensson, Magdalena S. (35225488000); Nijman, Vincent (6603777959)	Seizures of hundreds of jaguar heads and canines in Central and South America from 2014 to 2018 resulted in worldwide media coverage suggesting that wildlife traffickers are trading jaguar body parts as substitutes for tiger parts to satisfy the demand for traditional Asian medicine. We compiled a data set of >1000 seized wild cats (jaguar [Panthera onca], puma [Puma concolor], and ocelot [Leopardus pardalis]) from 19 Central and South American countries and China. We ran generalized additive mixed models to detect trends in wild-cat seizures from 2012 to 2018 and assess the effects of socioeconomic factors of source countries and between	Cat-related

				those countries and China on the number of wild cats seized. Jaguar seizures increased over time, and most of the seized jaguar pieces were canines (1991 of 2117). Around 34% (32 of 93) of the jaguar-part seizure reports were linked with China, and these seizures contained 14-fold more individuals than those intended for domestic markets. Source countries with relatively high levels of corruption and Chinese private investment and low income per capita had 10–50 times more jaguar seizures than the remaining sampled countries. The number of Chinese residents in Central and South America was not significantly related to the number of jaguars seized. No socioeconomic factors influenced the seizures of puma and ocelots. Legal market chains may provide structure for the illegal chain; thus, the influx of illegal jaguar products is potentially a side effect of the	

				economic partnership between Central and South American countries and China. Poverty and high levels of corruption in the source countries may motivate local people to engage in illegal activities and contribute to the growth of this trade. Supply-side interventions to curb this threat to Neotropical wild cats may include improved training for officials and promotion of governance and the value of protecting these animals to local people. © 2020 The Authors. Conservation Biology published by Wiley Periodicals LLC on behalf of Society for Conservation Biology	
WOS	10.1038/s41598-021-98899-5	Efficacy and side effects of bio-fabricated sardine fish scale silver nanoparticles against malarial vector Anopheles stephensi	Murugan, K; Subramaniam, J; Rajaganesh, R; Panneerselvam, C; Amuthavalli, P; Vasanthakumaran, M; Jayashanthini, S; Dinesh, D; Anitha, J; Wang, L; Hwang, JS; Dahms, HU; Mudigonda, S; Aziz, A	Mosquitoes are a great menace for humankind since they transmit pathogenic organisms causing Malaria, Dengue, Chikungunya, Elephantiasis and Japanese encephalitis. There is an urgent need to discover new and novel biological tools to mitigate mosquito-borne diseases. To develop	Cat-related

				bioinsecticides through newly developed nanotechnology is another option in the present research scenario. In this study we synthesize and characterize sardine fish scales with silver nitrate by adopting various instrumental techniques such as UV- and FTIR-spectroscopy, energy-dispersive X-ray (EDAX), X-ray diffraction analyses (XRD) and scanning electron microscopy (SEM). Toxicity bioassays were conducted with young developmental stages of mosquito vectors. Significant mortality appeared after different life stages of mosquito vectors (young larval and pupal instars were exposed to the nanomaterials). LC50 values were 13.261 ppm for young first instar larvae and 32.182 ppm for pupae. Feeding and predatory potential of G. affinis, before and after exposure to nanoparticles against mosquito larval (I & II) instars of the	

				mosquitoes showed promising results in laboratory experiments. Feeding potential of mosquito fish without nanoparticle treatment was 79.7% and 70.55% for the first and second instar larval populations respectively. At the nanoparticle-exposed situation the predatory efficiency of mosquitofish was 94.15% and 84.3%, respectively. Antioxidant enzymes like (SOD), (CAT), and (LPO) were estimated in the gill region of sardine fish in control and experimental waters. A significant reduction of egg hatchability was evident after nanoparticle application. It became evident from this study that the nano-fabricated materials provide suitable tools to control the malaria vector Anopheles stephensi in the aquatic phase of its life cycle. This finding suggests an effective novel approach to mosquito control.	
WOS	10.1071/WR14225	Nest location influences hatching success in the Socotra Cormorant (Phalacrocorax nigrogularis)	Muzaffar, SB; Gubiani, R; Benjamin, S	Context. Nest location is an important determinant of reproductive success in many seabirds. Better	EA

		on Siniya Island, United Arab Emirates		understanding of breeding ecology of threatened seabirds could help in their conservation. Aims. We evaluated nest location and reproductive success in the globally Vulnerable, regionally endemic, Socotra cormorants on Siniya Island, United Arab Emirates, in relation to multiple habitat factors to assess the relative influence of each. Methods. We measured hatching success, as an indication of reproductive success, on 14 study plots during the 2011-12 breeding season. We measured distances from each study plot to the nearest tree, road, predation area, sandy beach and lagoon. We used multiple logistic regression to determine the extent to which these explanatory factors influenced hatching success. We used an information theoretic approach to select the best model. Key results. Hatching success was significantly related to tree cover, distance from	

				beach and distance from heavily depredated areas (AIC(c) weight = 0.613). Nests were significantly deeper (13.14 +/- 0.35 cm) in unshaded areas compared with areas shaded by trees (10.8 +/- 0.31 cm) (ANOVA: F-2,F-12 = 24.93, P < 0.001). Conclusions. Thus, we conclude that shade provided by planted non-native trees is an important factor affecting nest location and hatching success. Deeper nests could aid incubation by lowering nest temperatures during peak incubation periods (August) when temperatures may exceed 48 degrees C. Heavy predation pressure is affecting hatching success. Wind action could be preventing nesting near the sandy beach. Implications. Planted non-native trees are advantageous to breeding Socotra cormorants, although planting trees is not advocated. High predation pressure from feral cats and red foxes is reducing hatching	

				success of Socotra cormorants on the largest known colony in the United Arab Emirates. Feral cats should be eradicated whereas the red foxes could be managed as a precautionary measure to ensure the continued survival of this threatened seabird.	
SCOPUS	2890771	Do faecal odours enable domestic cats (Felis catus) to distinguish familiarity of the donors?	Nakabayashi, Miyabi (54879645600); Yamaoka, Ryohei (7003972326); Nakashima, Yoshihiro (23991047400)	We studied the ability of domestic cats to distinguish familiarity based on faecal odours. This was evaluated by comparing the sniffing duration of cats' own, familiar, and unfamiliar faeces. We found that (1) sniffing durations differed between unfamiliar faeces and the other types of faeces, (2) sniffing durations of faeces of the same unfamiliar individuals decreased over time, and (3) sniffing durations toward unfamiliar faeces increased after change of donors. These results indicate that domestic cats can distinguish the faecal odours based on familiarity. This ability could be adaptive for domestic cats to maintain	Cat-related

				their social relationships. © 2011 Japan Ethological Society and Springer.	
SCOPUS	8888892	A review of feral cat eradication on islands	Nogales M.; Martín A.; Tershy B.R.; Donlan C.J.; Veitch D.; Puerta N.; Wood B.; Alonso J.	Feral cats are directly responsible for a large percentage of global extinctions, particularly on islands. We reviewed feral cat eradication programs with the intent of providing information for future island conservation actions. Most insular cat introductions date from the nineteenth and twentieth centuries, whereas successful eradication programs have been carried out in the last 30 years, most in the last decade. Globally, feral cats have been removed from at least 48 islands: 16 in Baja California (Mexico), 10 in New Zealand, 5 in Australia, 4 in the Pacific Ocean, 4 in Seychelles, 3 in the sub-Antarctic, 3 in Macaronesia (Atlantic Ocean), 2 in Mauritius, and 1 in the Caribbean. The majority of these islands (75%; n = 36) are small (≤5 km2). The largest successful eradication campaign took place on Marion	EA

				Island (290 km2), but cats have been successfully removed from only 10 islands (21%) of ≥10 km2. On Cousine Island (Seychelles) cat density reached 243 cats/km2, but on most islands densities did not exceed 79.2 cats/km2 (n = 22; 81%). The most common methods in successful eradication programs were trapping and hunting (often with dogs; 91% from a total of 43 islands). Frequently, these methods were used together. Other methods included poisoning (1080; monofluoracetate in fish baits; n = 13; 31%), secondary poisoning from poisoned rats (n = 4; 10%), and introduction of viral disease (feline panleucopaenia; n = 2; 5%). Impacts from cat predation and, more recently, the benefits of cat eradications have been increasingly documented. These impacts and benefits, combined with the continued success of eradication campaigns on	

				larger islands, show the value and role of feral cat eradications in biodiversity conservation. However, new and more efficient techniques used in combination with current techniques will likely be needed for success on larger islands.	
SCOPUS	9320113	Multiplex PCR system for identifying the carnivore origins of faeces for an epidemiological study on Echinococcus multilocularis in Hokkaido, Japan	Nonaka, Nariaki (7004943348); Sano, Takafumi (23482731500); Inoue, Takashi (59077145600); Teresa Armua, Maria (34873739900); Fukui, Daisuke (56333514300); Katakura, Ken (7005902529); Oku, Yuzaburo (7102490769)	A multiplex PCR system was developed to identify the carnivore origins of faeces collected in Hokkaido, Japan, for epidemiological studies on Echinococcus multilocularis. Primers were designed against the D-loop region of mitochondrial DNA. Two separate primer mixtures (mix 1, specific forward primers to fox, raccoon dog and dog, and a universal reverse primer [prH]; and mix 2, specific forward primers to cat, raccoon and weasels and prH) were used so that the PCR products (160 bp, fox and cat; 240 bp, raccoon dog and raccoon; and 330 bp, dog and weasel) were distinguished by size. The multiplex PCR exhibited no cross-	Cat-related

				reactivity between carnivore species and did not amplify DNA from rodent prey. When 270 field-collected faeces were examined, 250 showed single PCR products belonging to specific target sizes, suggesting successful carnivore identification for 92.6% of samples. Taeniid eggs were detected in 11.1% of samples and coproantigen in 30.4%; whereas the prevalences of taeniid eggs and coproantigen were 12.9% and 34.0% in fox faeces, and 0% and 26.3% in cat faeces, respectively. These results suggest that the prevalence in different target animals can be evaluated individually and precisely using multiplex PCR system. © 2009 Springer-Verlag.	
SCOPUS	1975020X	Effects of human activities on home range size and habitat use of the Tsushima leopard cat Prionailurus bengalensis euptilurus in a suburban area on the Tsushima islands, Japan	Oh, Dae-Hyun (56937749900); Moteki, Shusaku (36911436100); Nakanish, Nozomi (36911447400); Izawa, Masako (7101781352)	The Tsushima leopard cat, Prionailurus bengalensis euptilurus, a small felid, inhabits only the Tsushima Islands in Japan. Previous studies of the Tsushima leopard cat revealed that natural factors; including sex,	Cat-related

				reproductive activity, season, and prey distribution and abundance affect leopard cat home range variation and habitat use. In this study, we focused on clarifying how anthropogenic factors influenced home range variation and habitat use of a male Tsushima leopard cat living near a suburban area in January, March, May and September 2005 using radio-tracking. The home range size (100% MCP) of this cat was 0.78 ± 0.26 km2 (mean ± SD, n = 4 tracking sessions) across the whole study period. However, the cat did not use all parts of its home range uniformly; rather it used some habitat types selectively. The cat avoided agriculture areas and residential areas in all of the tracking-sessions. On the other hand, the cat showed a weak preference for artificial structures and a strong preference for baiting sites in January and March, while it avoided them in May, and no	

				baiting site was included in its home range in September. These results suggest that anthropogenic factors influenced the ranging patterns and habitat use of the leopard cat living near a suburban area. Artificial structures might provided good resting spaces for the cat in bad weather. When the density of its main prey was low in the winter, the cat tended to rely on artificial prey and had a small home range size.	
SCOPUS	22132244	Role of landscape context in Toxoplasma gondii infection of invasive definitive and intermediate hosts on a World Heritage Island	Okada, Sono (57881913400); Shoshi, Yuki (57222239436); Takashima, Yasuhiro (7103176256); Sanjoba, Chizu (6505517121); Watari, Yuya (18434958100); Miyashita, Tadashi (7201920714)	Free-ranging cats are invasive species threatening biodiversity worldwide. They may also impose an environmental risk to humans and livestock through the transmission of zoonotic diseases. We investigated antibody levels against Toxoplasma gondii in free-ranging cats and black rats (definitive and representative intermediate hosts) by ELISA and determined their relationships with landscape environmental factors on Tokunoshima Island, Japan, the Natural	Cat-related

				World Heritage site. We found a higher seroprevalence (>70%) in both cats and black rats in landscapes where the cattle barn density was high. This was consistent with higher density of rats revealed in our trapping survey. The spatial scale of landscape factors affecting infection was broader in cats (1 km buffer radius) than in black rats (100 m buffer radius). Both cats and rats showed an increasing trend in optical density (OD) values with increasing body weight and landscape cattle barn density, suggesting that the antibody concentration increases as the chance of exposure to T. gondii in the environment increases. Thus, management actions to stop humans from feeding cats and to control rat populations without using cats are both necessary to reduce the human health risk as well as to conserve endangered species on the island. © 2022 The Authors	

SCOPUS	19326203	Environmental impacts of food consumption by dogs and cats	Okin, Gregory S. (6602377076)	In the US, there are more than 163 million dogs and cats that consume, as a significant portion of their diet, animal products and therefore potentially constitute a considerable dietary footprint. Here, the energy and animal-derived product consumption of these pets in the US is evaluated for the first time, as are the environmental impacts from the animal products fed to them, including feces production. In the US, dogs and cats consume about 19% ± 2% of the amount of dietary energy that humans do (203 ± 15 PJ yr-1 vs. 1051 ± 9 PJ yr-1) and 33% ± 9% of the animal-derived energy (67 ± 17 PJ yr-1 vs. 206 ± 2 PJ yr-1). They produce about 30% ± 13%, by mass, as much feces as Americans (5.1 ± Tg yr-1 vs. 17.2 Tg yr-1), and through their diet, constitute about 25–30% of the environmental impacts from animal production in terms of the use of land, water,	EA

				fossil fuel, phosphate, and biocides. Dog and cat animal product consumption is responsible for release of up to 64 ± 16 million tons CO2-equivalent methane and nitrous oxide, two powerful greenhouse gasses (GHGs). Americans are the largest pet owners in the world, but the tradition of pet ownership in the US has considerable costs. As pet ownership increases in some developing countries, especially China, and trends continue in pet food toward higher content and quality of meat, globally, pet ownership will compound the environmental impacts of human dietary choices. Reducing the rate of dog and cat ownership, perhaps in favor of other pets that offer similar health and emotional benefits would considerably reduce these impacts. Simultaneous industry-wide efforts to reduce overfeeding, reduce waste, and find alternative sources of	

				protein will also reduce these impacts. © 2017 Gregory S. Okin. This is an open access article distributed under the terms of the Creative Commons Attribution License, which permits unrestricted use, distribution, and reproduction in any medium, provided the original author and source are credited.	
WOS	10.1002/ece3.11016	New insights into predator-prey dynamics: First evidence of a leopard cat hunting coypus	Park, HB; Lim, A	We present the first documented evidence of interactions between the leopard cat (Prionailurus bengalensis euptilurus) and the invasive coypu (Myocastor coypus) in South Korea, captured through camera traps in Hwapocheon wetland park from May 2015 to April 2017. Two interactions were recorded: one showing a leopard cat carrying a carcass of sub-adult coypu and the other a 4-min sequence of predation and defense between two species. The observed interactions indicate active predatory behavior by the leopard cat against coypus and cooperative defense by	Cat-related

				coypus. These findings shed new light on predator-prey dynamics, highlighting the leopard cat's potential role as a predator of coypus and coypus' defensive abilities. Understanding these relationships could facilitate more effective management of invasive species and offer broader implications for ecosystem dynamics and conservation strategies. This study provides the first documented evidence of interactions between the native leopard cat and the invasive coypu in South Korea, observed in the Hwapocheon wetland park from May 2015 to April 2017. Through camera traps, the research unveils active predatory behavior by leopard cats against coypus and the cooperative defense mechanisms exhibited by the coypus.image	
WOS	10.1016/j.geomorph.2007.04.028	Geomorphological and palaeoenvironmental investigations in the southeastern Arabian Gulf region and the implication for the archaeology of the region	Parker, AG; Goudie, AS	During the Late Quaternary, the climate of Arabia has fluctuated between periods of higher rainfall and fluvial activity, dominated by	Cat-related

				the influence of the Indian Ocean Monsoon (IOM) and drier/arid conditions under the influence of the westerlies. This has left a rich legacy of landforms from which temporal and spatial patterns of environmental change are reconstructed. The coastal desert region of the southeastern Arabian Gulf has been a focal point for human settlement since similar to 8000 cat yr BP. The region is strategically located on an important trade route between two 'cradles of civilization', namely, Mesopotamia and the Indus. Changes in the evolution and modification of this landscape under varying climatic conditions have influenced humans living in and exploiting this landscape for food and water, raw materials and trade routes. In this study, geomorphological and palaeoenvironmental investigations are integrated to provide a framework of environmental change for the Late Glacial and	

				early-mid Holocene periods against which the archaeology of the area can be set. The Late Glacial and earliest Holocene was characterised by intense aridity and accumulation of mega linear dunes driven by the Shamal winds. In the Arabian Gulf region, this continued into the earliest part of the Holocene, whilst southern Arabia was under the influence of the IOM. The monsoon rains migrated into the Gulf region between 8500-6000 cat BP. During this time, semi-nomadic herders occupied this region and grazing their animals in a landscape covered with C3 savanna grassland. The Neolithic peoples also practised hunting and fishing and the collection of shellfish was an important activity. Pottery shows links with Mesopotamia at this time. From 6000 cat BP the IOM retreated south and rainfall was derived from winterly westerly sources. Under drier conditions a switch	

				occurred to a sparser cover of C4 grasses. From 4500 cat BP the climate became much drier with the development of stronger westerly summer Shamal winds and the reactivation of dunes across the region. An intense and period occurred at 4100 BP which corresponds with major drought conditions in Mesopotamia and the Indus region, which led to major changes in society. This event occurs at a major transition in the Arabian Gulf Bronze Age archaeological record with a decline in population and trade. The last 4000 yr has largely been characterised by arid conditions similar to those found in the region today. (C) 2008 Elsevier B.V. All rights reserved.	
SCOPUS	1106465	Eradication of feral cats from large islands: An assessment of the effort required for success	Parkes, John (7103261878); Fisher, Penny (56257351000); Robinson, Sue (7402673879); Aguirre-Muñoz, Alfonso (23388733200)	Feral cats (Felis catus) are predators and competitors of native species on many islands and are therefore the target of control efforts. Cat eradication has been achieved on 83 islands worldwide. Six of these successes have been from	EA

				large islands (over 2000 ha) and have reported sufficient data to examine how the eradication was achieved through combinations of aerial and ground-based poison baiting, fumigation in rabbit burrows used by cats, cage and leghold trapping, day and night shooting, and hunting with dogs. No common sequence of tactics was deployed although leghold traps were used in the latter phases of most projects. It took a mean reported effort of 543 ± 341 person-days per 1000 ha of island over 5.2 ± 1.6 years to completely remove cats and validate success from the six islands. These precedents may assist in planning future proposals to eradicate cats from other large islands. © New Zealand Ecological Society.	
SCOPUS	1401963	Range size and den use of Gordon's wildcats Felis silvestris gordoni in the Emirate of Sharjah, United Arab Emirates	Phelan, P. (7102833490); Sliwa, A. (6603041984)	Two Gordon's wildcats (Felis silvestris gordoni; Harrison, The Mammals of Arabia, Vol. 2, Ernest Benn Ltd, London, 381pp) were radio-tracked over 1.5 months (male) and 14 months	Cat-related

				(female) in the desert of the Sharjah Emirate in the United Arab Emirates. The annual range of the female, using the 95% Minimum Convex Polygon (MCP95) estimator was 51.21km2. Seasonal ranges for the female averaged 19.24±14.6km2 (n=5) that of the male was 28.65km2. Monthly ranges (MCP95) of the female averaged 16.41±9.39km2 (n=12) and 21.36km2 (20.39 and 22.32km2 (n=2)) for the male. The male wildcat moved 8.64±2.40km/night (n=16, range 5.7-13.4km), which was further than the female that moved 4.86±1.32km/night (n=38, range 2.4-8.4km). Forty-two den sites were recorded and the majority of dens were used repeatedly. Most dens were on the dune slopes and had single entrances. The large ranges of these wildcats have serious conservation implications in the light of increasing human encroachment into the desert, often	

				trailed by feral cats that pose the threats of hybridisation and transmission of disease to wildcats. © 2004 Elsevier Ltd. All rights reserved.	
WOS	10.1080/01584197.2019.1686645	Patterns of nesting and nest success in an evergreen forest in Southeast Asia	Pierce, AJ; Sankamethawee, W; Powell, LA; Gale, GA	Southeast Asian forest birds are threatened by the highest rate of tropical deforestation globally, but conservation efforts regionally have very limited information from which to make policy and management plans. To provide baseline data on nesting and nest success for forest birds from an intact forest, we monitored 1506 nests of 54 species during 2003-2011 in a seasonally wet forest at 14 degrees N in north-eastern Thailand. We estimated daily nest survival (DSR) for 37 species (n = 1082 nests). Early nesting species were more successful and ground-nesting birds had higher DSR (0.962) than above-ground nests (0.934). DSR for 13 focal species (n = 956 nests) varied from 0.842 to 0.975. In general, DSR was unrelated to season	Cat-related

				or nest height for focal species although one species was more successful early in their breeding season while for another, higher nests were more successful. Nest survival was low, and predation accounted for 92% of nest failures. Two nest predators, Northern Pig-tailed Macaque Macaca nemestrina and Green Cat Snake Boiga cyanea accounted for 43.7% and 21.8% of the predation events, respectively. We suggest that local conditions, even in intact forests, may produce complex dynamics for reproductive success of breeding birds, and we posit that presence of the dominant nest predators was largely responsible for patterns we observed among species, particularly the higher DSR of ground-nesting birds. Our data further support the idea that DSR is generally lower in relatively undisturbed sites in the tropics than similarly undisturbed sites in the temperate zone.	

WOS	10.3197/096734016X14661540219393	People in Peril, Environments at Risk: Coolies, Tigers, and Colonial Singapore's Ecology of Poverty	Powell, MA	In recent years, scholars have demonstrated that affluent societies have a disproportionate environmental impact. A focus on wealth, however, can obscure how poverty also propels ecosystem destruction, particularly when combined—as in colonial Singapore with uncaring administrators and the ruthless logic of imperialism. In this colony, poor Chinese, Malays, archipelagic South-east Asians and Indians struggled to eke out a living by razing rainforest, because they possessed no better options. As these workers struggled to transform forests into farms, houses and roads, they created an ecology of poverty that had catastrophic consequences for humans and tigers alike. Finding ever less forest in which to hide from humans, the mighty cats instead began to regard some people as prey. Unable to flee, poor Chinese, Malays, archipelagic South-east Asians and Indians sought to protect	Cat-related

				themselves by killing tigers. In the end, the humans vanquished the cats, but not without enduring hundreds of fatalities. Colonial Singapore's environmental history reminds us that the people who carry out the work of eradicating nature often do so because they possess limited alternatives for survival. As a corollary, caring for and protecting the environment is inseparable from aiding and respecting people.	
SCOPUS	63207	An assessment of South China tiger reintroduction potential in Hupingshan and Houhe National Nature Reserves, China	Qin Y.; Nyhus P.J.; Larson C.L.; Carroll C.J.W.; Muntifering J.; Dahmer T.D.; Jun L.; Tilson R.L.	Human-caused biodiversity loss is a global problem, large carnivores are particularly threatened, and the tiger (Panthera tigris) is among the world's most endangered large carnivores. The South China tiger (Panthera tigris amoyensis) is the most critically endangered tiger subspecies and is considered functionally extinct in the wild. The government of China has expressed its intent to reintroduce a small population of South	Cat-related

				China tigers into a portion of their historic range as part of a larger goal to recover wild tiger populations in China. This would be the world's first major tiger reintroduction program. A free-ranging population of 15-20 tigers living in a minimum of 1000km2 of habitat was identified as a target. We assessed summer and winter habitat suitability of two critical prey species, wild boar (Sus scrofa) and Sika deer (Cervus nippon), using GIS spatial models to evaluate the potential for tiger reintroduction in one likely candidate site, the 1100km2 Hupingshan-Houhe National Nature Reserve complex in Hunan and Hubei Provinces, China. Our preliminary analysis estimates that for wild boar, potential summer and winter habitat availability is 372-714km2 and 256-690km2, respectively, whereas for Sika deer, potential summer and winter habitat availability	

				is 443-747km2 and 257-734km2, respectively. Our model identifies potential priority areas for release and restoration of prey between 195 and 790km2 with a carrying capacity of 596-2409 wild boar and 468-1929 Sika deer. Our analysis suggests that Hupingshan-Houhe could support a small population of 2-9 tigers at a density of 1.1-1.2 tigers/100km2 following prey and habitat restorations. Thus, current habitat quality and area would fall short of the target recovery goal. We identify major challenges facing a potential tiger reintroduction project and conclude that restoring the habitat and prey base, addressing concerns of local people, and enhancing coordination across park boundaries are significant challenges to meeting the broader goals of supporting a reintroduced wild tiger population. Tiger range states have committed to doubling the world's wild tigers by	

				2022. The results of this study have implications for China's commitment to this goal and for the future of tiger and other large carnivore reintroduction efforts in Asia and globally. © 2014 The Authors.	
SCOPUS	17449561	Primate communication in the pure ultrasound	Ramsier, Marissa A. (35339788000); Cunningham, Andrew J. (55232877200); Moritz, Gillian L. (54882767600); Finneran, James J. (7003866889); Williams, Cathy V. (7406217530); Ong, Perry S. (24587869600); Gursky-Doyen, Sharon L. (7006230908); Dominy, Nathaniel J. (6603936273)	Few mammals-cetaceans, domestic cats and select bats and rodents-can send and receive vocal signals contained within the ultrasonic domain, or pure ultrasound (greater than 20 kHz). Here, we use the auditory brainstem response (ABR) method to demonstrate that a species of nocturnal primate, the Philippine tarsier (Tarsius syrichta), has a high-frequency limit of auditory sensitivity of ca 91 kHz. We also recorded a vocalization with a dominant frequency of 70 kHz. Such values are among the highest recorded for any terrestrial mammal, and a relatively extreme example of ultrasonic communication. For Philippine tarsiers, ultrasonic vocalizations	Cat-related

				might represent a private channel of communication that subverts detection bypredators, prey and competitors, enhances energetic efficiency, or improves detection against low-frequency background noise. This journal is © 2012 The Royal Society.	
J-Stage+CiNii		Alien Mammals, raccoon dog, feral dog, feral cat, and feral goat in western and north-western part of lowland forest on Yakushima Island in Kagoshima Prefecture	Riyou Tsujino and Yoshimi Yanagihara Agetsuma	-	Predation-related
SCOPUS	14242818	Genetic Variation in the Pallas's Cat (Otocolobus manul) in Zoo-Managed and Wild Populations	Robinson J.J.; Crichlow A.D.; Hacker C.E.; Munkhtsog B.; Munkhtsog B.; Zhang Y.; Swanson W.F.; Lyons L.A.; Janecka J.E.	The Pallas's cat (Otocolobus manul) is one of the most understudied taxa in the Felidae family. The species is currently assessed as being of "Least Concern" in the IUCN Red List, but this assessment is based on incomplete data. Additional ecological and genetic information is necessary for the long-term in situ and ex situ conservation of this species. We identified 29 microsatellite loci with sufficient diversity to enable studies into the	Cat-related

				individual identification, population structure, and phylogeography of Pallas's cats. These microsatellites were genotyped on six wild Pallas's cats from the Tibet Autonomous Region and Mongolia and ten cats from a United States zoo-managed population that originated in Russia and Mongolia. Additionally, we examined diversity in a 91 bp segment of the mitochondrial 12S ribosomal RNA (MT-RNR1) locus and a hypoxia-related gene, endothelial PAS domain protein 1 (EPAS1). Based on the microsatellite and MT-RNR1 loci, we established that the Pallas's cat displays moderate genetic diversity. Intriguingly, we found that the Pallas's cats had one unique nonsynonymous substitution in EPAS1 not present in snow leopards (Panthera uncia) or domestic cats (Felis catus). The analysis of the zoo-managed population indicated reduced genetic diversity	

				compared to wild individuals. The genetic information from this study is a valuable resource for future research into and the conservation of the Pallas's cat. © 2024 by the authors.	
WOS	10.1139/Z10-056	Den-site selection is critical for Pallas's cats (Otocolobus manul)	Ross, S; Kamnitzer, R; Munkhtsog, B; Harris, S	We evaluated the habitat selection of 101 den sites used by 21 individual Pallas's cats (Otocolobus manul (Pallas, 1776) = Felis manul Pallas, 1776) in summer, winter, and the maternal period in central Mongolia using generalized linear mixed models. Pallas's cats used rock crevices and marmot burrows as dens for giving birth, raising young, thermoregulation, feeding, mating, and as important cover from predators. Den sites were selected with higher proportions of rocky and ravine habitats in the surroundings, and in winter Pallas's cats avoided the presence of humans. Habitat and structural features suggested that dens were selected to minimize predation risk. Selection of dens in shade in	Cat-related

				summer and the use of insulated dens of Siberian marmots (Marmota sibirica (Radde, 1862)) in winter indicated that thermal properties may also be important. We contend that dens are a critical habitat for Pallas's cats and the availability of suitable den sites is critical for the conservation of the species. Repeated use of maternal dens suggested they may be a limiting resource. Although marmot dens are unlikely to be limiting at present, over hunting of marmots is likely to reduce burrow availability in the future.	
WOS	10.1644/09-MAMM-A-342.1	Dietary composition, plasticity, and prey selection of Pallas's cats	Ross, S; Munkhtsog, B; Harris, S	Dietary plasticity and diet composition of Pallas's cat (Otocolobus manul) was quantified in central Mongolia. Diet of Pallas's cat was assessed by scat analyses, and prey surveys were used to estimate prey availability. Prey selection was calculated using multinomial likelihood ratio tests. Analysis of 146 scats identified 249 prey items. Pallas's cats ate a broad range of small mammals,	Cat-related

				insects, birds, reptiles, and carrion, but Daurian pikas (Ochotona dauurica) were the most frequently consumed prey. Multinomial likelihood ratio tests indicated nonrandom selection of prey species. Pikas were selected disproportionately to their availability, and other more numerous prey items were used less than expected, indicating feeding specialization. Specialization on pikas appears to optimize energy intake per unit foraging by Pallas's cats, because pikas are 2-4 times larger than other small mammal prey. We argue that pika control programs in China and Mongolia potentially threaten Pallas's cat populations, because no other prey species could fill the niche occupied by the pika in terms of size and year-round availability. DOT: 10.1644/09-MAMM-A-342.1.	
WOS	10.1098/rsos.171187	An adaptable but threatened big cat: density, diet and prey selection of the Indochinese leopard (Panthera pardus	Rostro-García, S; Kamler, JF; Crouthers, R; Sopheak, K; Prum,	We studied the Indochinese leopard (Panthera pardus delacouri) in eastern	Cat-related

		delacouri) in eastern Cambodia	S; In, V; Pin, C; Caragiulo, A; Macdonald, DW	Cambodia, in one of the few potentially remaining viable populations in Southeast Asia. The aims were to determine the: (i) current leopard density in Srepok Wildlife Sanctuary (SWS) and (ii) diet, prey selection and predation impact of leopard in SWS. The density, estimated using spatially explicit capture-recapture models, was 1.0 leopard/100km(2), 72% lower than an estimate from 2009 at the same site, and one of the lowest densities ever reported in Asia. Dietary analysis of 73 DNA confirmed scats showed leopard consumed 13 prey species, although ungulates comprised 87% of the biomass consumed (BC). The overall main prey (42% BC) was banteng (Bos javanicus), making this the only known leopard population whose main prey had adult weight greater than 500 kg. Consumption of wild pig (Sus scrofa) was also one of the highest ever reported (22% BC), indicating leopard	

				consistently predated on ungulates with some of the largest adult weights in SWS. There were important differences in diet and prey selection between sexes, as males consumed mostly banteng (62% BC) in proportion to availability, but few muntjac (Muntiacus vaginalis; 7% BC), whereas females selectively consumed muntjac (56% BC) and avoided banteng (less than 1% BC). Predation impact was low (0.5-3.2% of populations) for the three ungulate species consumed. We conclude that the Indochinese leopard is an important apex predator in SWS, but this unique population is declining at an alarming rate and will soon be eradicated unless effective protection is provided.	
SCOPUS	489697	The assessment of daily dietary intake reveals the existence of a different pattern of bioaccumulation of chlorinated pollutants between domestic dogs and cats	Ruiz-Suárez, Norberto (55376446600); Camacho, María (34067495700); Boada, Luis D. (6603916807); Henríquez-Hernández, Luis A.	Pet dogs and cats have been proposed as sentinel species to assess environmental contamination and human exposure to a variety of pollutants, including POPs. However, some authors	Predation-related

			(15829708200); Rial, Cristian (56610404900); Valerón, Pilar F. (6603469417); Zumbado, Manuel (6603459604); González, Maira Almeida (37031058800); Luzardo, Octavio P. (6507534124)	have reported that dogs but not cats exhibit intriguingly low levels of some of the most commonly detected POPs, such as DDT and its metabolites. This research was designed to explore these differences between dogs and cats. Thus, we first determined the concentrations of 53 persistent and semi-persistent pollutants (16 polycyclic aromatic hydrocarbons (PAHs), 18 polychlorinated biphenyls (PCBs) and 19 organochlorine pesticides (OCPs)) in samples of the most consumed brands of commercial feed for dogs and cats, and we calculated the daily dietary intake of these pollutants in both species. Higher levels of pollutants were found in dog food and our results showed that the median values of intake were about twice higher in dogs than in cats for all the three groups of pollutants (σPAHs: 274.8 vs. 141.8; σOCPs: 233.1 vs. 83; σPCBs: 101.8 vs. 43.8 (ng/kg. bw/day); respectively).	

				Additionally, we determined the plasma levels of the same pollutants in 42 and 35 pet dogs and cats, respectively. All these animals lived indoors and were fed on the commercial brands of feed analyzed. As expected (considering the intake), the plasma levels of PAHs were higher in dogs than in cats. However, for organochlorines (OCPs and PCBs) the plasma levels were much higher in cats than in dogs (as much as 23 times higher for DDTs), in spite of the higher intake in dogs. This reveals a lower capacity of bioaccumulation of some pollutants in dogs, which is probably related with higher metabolizing capabilities in this species. © 2015.	
SCOPUS	19326203	Distribution of Wild Mammal Assemblages along an Urban-Rural-Forest Landscape Gradient in Warm-Temperate East Asia	Saito, Masayuki (57225965573); Koike, Fumito (7005053303)	Urbanization may alter mammal assemblages via habitat loss, food subsidies, and other factors related to human activities. The general distribution patterns of wild mammal assemblages along urban-	Cat-related

				rural-forest landscape gradients have not been studied, although many studies have focused on a single species or taxon, such as rodents. We quantitatively evaluated the effects of the urban-rural-forest gradient and spatial scale on the distributions of large and mid-sized mammals in the world's largest metropolitan area in warm-temperate Asia using nonspecific camera-trapping along two linear transects spanning from the urban zone in the Tokyo metropolitan area to surrounding rural and forest landscapes. Many large and mid-sized species generally decreased from forest landscapes to urban cores, although some species preferred anthropogenic landscapes. Sika deer (Cervus nippon), Reeves' muntjac (Muntiacus reevesi), Japanese macaque (Macaca fuscata), Japanese squirrel (Sciurus lis), Japanese marten (Martes melampus), Japanese	

				badger (Meles anakuma), and wild boar (Sus scrofa) generally dominated the mammal assemblage of the forest landscape. Raccoon (Procyon lotor), raccoon dog (Nyctereutes procyonoides), and Japanese hare (Lepus brachyurus) dominated the mammal assemblage in the intermediate zone (i.e., rural and suburban landscape). Cats (feral and free-roaming housecats; Felis catus) were common in the urban assemblage. The key spatial scales for forest species were more than 4000-m radius, indicating that conservation and management plans for these mammal assemblages should be considered on large spatial scales. However, small green spaces will also be important for mammal conservation in the urban landscape, because an indigenous omnivore (raccoon dog) had a smaller key spatial scale (500-m radius) than those of forest mammals. Urbanization was	

				generally the most important factor in the distributions of mammals, and it is necessary to consider the spatial scale of management according to the degree of urbanization. © 2013 Saito, Koike.	
SCOPUS	9167250	Prevalence of toxoplasma gondii and other intestinal parasites in cats in tokachi sub-prefecture, Japan	Salman, Doaa (56245446100); Pumidonming, Wilawan (6506731194); Oohashi, Eiji (6508021794); Igarashi, Makoto (35573757300)	The present study investigated the prevalence of Toxoplasma gondii and other intestinal parasites in cats in the Tokachi subprefecture in Japan. A total of 365 household cats were included in the study, and 353 serum and 351 fecal samples were collected and analyzed. T. gondii IgG antibodies were detected in the sera of 16.14% of cats based on Latex agglutination test and ELISA. For ELISA, T. gondii RH strain tachyzoites lysate and T. gondii SAG2 recombinant protein were used as antigens. Low seropositivity was detected in cats younger than one year and older than 11 years; outdoor and hunter cats showed significantly high seropositivities.	Predation -related

				Neutering either in male or female cats, but not gender, had a considerable effect on seroprevalence. Toxoplasma gondii oocysts were detected in one fecal sample. The overall parasitic infestation in cats was 12.5%. Other detected parasites included Toxocara species, which showed the highest prevalence of 7.7%, followed by Isospora spp. (2%), Taenia spp. (1.7%), and Ancylostoma spp. (0.9%). Spirometra spp. was detected in only one sample. Outdoor cats comprised 50% of all 44 parasite-infested cats. Although T. gondii oocysts were detected in only one sample, the relatively high seroprevalence of T. gondii indicated that it can pose significant risks to the environment. Our findings highlighted the potential of outdoor cats as a source of T. gondii and other parasites. © 2018 The Japanese Society of Veterinary Science.	

SCOPUS	220493	Impacts of residual insecticide barriers on perimeter-invading ants, with particular reference to the odorous house ant, Tapinoma sessile	Scharf M.E.; Ratliff C.R.; Bennett G.W.	Three liquid insecticide formulations were evaluated as barrier treatments against perimeter-invading ants at a multifamily housing complex in West Lafayette, IN. Several ant species were present at the study site, including (in order of abundance) pavement ant, Tetramorium caespitum (L.); honey ant, Prenolepis imparis (Say); odorous house ant, Tapinoma sessile (Say); thief ant, Solenopsis molesta (Say); acrobat ant, Crematogaster ashmeadi (Mayr); crazy ant, Paratrechina longicornis (Latrielle), field ants, Formica spp.; and carpenter ant Camponotus pennsylvanicus (DeGeer). Studies began in May 2001 and concluded 8 wk later in July. Individual replicate treatments were placed 0.61 m (2 feet) up and 0.92 m (3 feet) out from the ends of 46.1 by 10.1-m (151 by 33-foot) apartment buildings. Ant sampling was performed with 10 placements of	Cat-related

				moist cat food for 1 h within treatment zones, followed by capture and removal of recruited ants for later counting. All treatments led to substantial reductions in ant numbers relative to untreated controls. The most effective treatment was fipronil, where 2% of before-treatment ant numbers were present at 8 wk after treatment. Both imidacloprid and cyfluthrin barrier treatments had efficacy comparative with fipronil, but to 4 and 2 wk, respectively. Odorous house ants were not sampled before treatment. Comparisons of ant species composition between treatments and controls revealed an increase in odorous house ant frequencies at 1-8 wk after treatment in treated locations only. These results demonstrate efficacy for both nonrepellent and repellent liquid insecticides as perimeter treatments for pest ants. In addition, our findings with odorous house ant	

				highlight an apparent invasive-like characteristic of this species that may contribute to its dramatic increase in structural infestation rates in many areas of the United States.	
SCOPUS	2890771	The reproductive tactics and activity patterns of solitary carnivores: The Iriomote cat	Schmidt, Krzysztof (7403914732); Nakanishi, Nozomi (7202974472); Izawa, Masako (7101781352); Okamura, Maki (7202356776); Watanabe, Shinichi (59035935700); Tanaka, Sachiko (57218976812); Doi, Teruo (7402078944)	Felids are generally considered to be crepuscular and nocturnal in their activity, but few studies have attempted to analyze the variability of their activity patterns. We studied the daily activity of the Iriomote cat Prionailurus iriomotensis by radio-tracking on Iriomote Island, Japan. The general activity patterns of Iriomote cats showed slightly prevailing activity during dark periods of the day with particular peaks at dawn and dusk or during the early hours of the night. However, these patterns were clearly dependent on the sex and reproductive status of the cat. Peaks of cats' activity coincided with those of their main prey. On average, the cats were active for thirteen hours per day. During the	Cat-related

				mating season, the rhythm of activity in males followed that of breeding females, but not that of non-breeding ones. Males exhibited 11% higher total daily activity and longer active bouts during the mating period than in the remainder of the year. Breeding females had additional mid-day activity peak during the nursing period, but their total time of activity per day was 16% lower than in the period of kittens' independence. Their active bouts were shorter and more frequent during nursing than at other times. These results suggest that lactating females perform frequent movements to and from the den site to care for kittens. During the non-nursing period, females increased their activity, possibly in response to lowered prey abundance and the need of intensive foraging to recover after lactation. Seasonal and sexual variation of activity patterns in the Iriomote cats confirmed the existence of different	

				reproductive strategies of males and females of these solitary carnivores. © 2008 Japan Ethological Society and Springer.	
SCOPUS	9528369	Movements and use of home range in the Iriomote cat (Prionailurus bengalensis iriomotensis)	Schmidt, Krzysztof (7403914732); Nakanishi, Nozomi (7202974472); Okamura, Maki (7202356776); Doi, Teruo (7402078944); Izawa, Masako (7101781352)	Movement pattern of the Iriomote cat Prionailurus bengalensis iriomotensis was studied on Iriomote Island, Japan by radio-tracking. The influence of sex and reproductive status of cats on their daily movement distance (DMD), straight-line distance (SLD), daily movement range (DMR) and other relative indices of movements was estimated. DMD was longest in males (3.2 km) and shortest in females without kittens (1.9 km). Also, males moved faster than either females with or without kittens (0.34, 0.25 and 0.25 km/h, respectively). During the mating season, males moved faster and their DMRs were larger than during the rest of the year. The SLD/DMD ratio in males was similarly high in both seasons (0.29 and 0.33), which indicates that their daily routes were	Cat-related

				expanded over large areas year-round, possibly to maintain a continuous control over their home range. In contrast, SLD/DMD varied seasonally in breeding females. It was low (0.09) during the lactating period, when females returned repeatedly to the den with kittens and it became high (0.32) out of the breeding season. Breeding females moved equally long DMDs in both seasons, whereas non-breeding ones moved shorter distances during the breeding period. These differences may be related to seasonal changes of food abundance. Cats moved intensively, as indicated by long (812-1139 m) routes per km2 of their home range, and occasionally visited distant parts of their ranges in a short time (4-5 days). On the other hand, they showed a relatively slow increase of the patrolled area, from 10% to 36% of their home ranges, in the first and fifth day,	

				respectively. Movement pattern of Iriomote cats showed no clear relation to active defence of home ranges.	
SCOPUS	19326203	Prey preference of snow leopard (panthera uncia) in south gobi, mongolia	Shehzad W.; McCarthy T.M.; Pompanon F.; Purevjav L.; Coissac E.; Riaz T.; Taberlet P.	Accurate information about the diet of large carnivores that are elusive and inhabit inaccessible terrain, is required to properly design conservation strategies. Predation on livestock and retaliatory killing of predators have become serious issues throughout the range of the snow leopard. Several feeding ecology studies of snow leopards have been conducted using classical approaches. These techniques have inherent limitations in their ability to properly identify both snow leopard feces and prey taxa. To examine the frequency of livestock prey and nearly-threatened argali in the diet of the snow leopard, we employed the recently developed DNA-based diet approach to study a snow leopard population located in the Tost Mountains, South Gobi, Mongolia. After DNA	Cat-related

				was extracted from the feces, a region of ~100 bp long from mitochondrial 12S rRNA gene was amplified, making use of universal primers for vertebrates and a blocking oligonucleotide specific to snow leopard DNA. The amplicons were then sequenced using a next-generation sequencing platform. We observed a total of five different prey items from 81 fecal samples. Siberian ibex predominated the diet (in 70.4% of the feces), followed by domestic goat (17.3%) and argali sheep (8.6%). The major part of the diet was comprised of large ungulates (in 98.8% of the feces) including wild ungulates (79%) and domestic livestock (19.7%). The findings of the present study will help to understand the feeding ecology of the snow leopard, as well as to address the conservation and management issues pertaining to this wild cat. © 2012 Shehzad et al.	

SCOPUS	2890003	Genetic identification of mammalian carnivore species in the Kushiro Wetland, eastern Hokkaido, Japan, by analysis of fecal DNA	Shimatani Y.; Takeshita T.; Tatsuzawa S.; Ikeda T.; Masuda R.	To identify mammalian carnivore species distributed in the Kushiro Wetland, eastern Hokkaido, Japan, we developed molecular-genetic methods for identification of the species from fecal samples collected from the field. Species-specific primers and PCR programs were established for five native and six alien species of carnivores: Martes zibellina, Mustela nivalis, Mustela erminea, Vulpes vulpes, and Nyctereutes procyonoides as native species, and Neovison vison, Martes melampus, Mustela itatsi, Canis familiaris, Felis catus, and Procyon lotor as alien species in Hokkaido. Touchdown PCR, in which the annealing temperature is decreased 1°C every cycle, was more effective for some species from which fecal DNA was not amplified species-specifically with standard PCR programs. Of 405 fecal samples collected from the Kushiro Wetland, the species of	Cat-related

				origin of 246 samples were successfully identified: 88 samples for N. vison, 140 for M. zibellina, 13 for V. vulpes, four for C. familiaris and one for F. catus. The results show the particular applicability of this method to monitoring M. zibellina and N. vison. In addition, methods to PCR-amplify DNA from two crayfish species (Pacifastacus leniusculus and Cambaroides japonicus) were developed to determine whether the carnivore fecal samples contained detectable DNA from the prey crayfishes. DNA from P. leniusculus was amplified from feces of N. vison identified in the present study, but no DNA from C. japonicus was detected. This indicates that N. vison preys on the alien species P. leniusculus. © 2008 Zoological Society of Japan.	
SCOPUS	3790738	A novel microsatellite (STR) marker for forensic identification of big cats in India	Singh A.; Gaur A.; Shailaja K.; Bala B.S.; Singh L.	India is the home to five of the eight majestic big cats of the world. The three major big cats namely, lion, tiger, and	Cat-related

				leopard are listed in the Schedule I of the Indian Wildlife Protection Act, 1972. Apart from the severe loss of the habitat, these are continuously facing the danger of extinction mainly due to poaching and hunting for their body parts, which are being greatly valued by apothecaries marketing traditional Chinese medicines. With the advent of polymerase chain reaction (PCR), DNA-based markers have emerged as major tools in the arena of wildlife forensics. Microsatellites (short tandem repeats, STRs) are markers of choice because of their polymorphic and co-dominant nature. These strictly follow the Mendelian inheritance and are highly reproducible. We have identified a new microsatellite (STR) locus Ple 46, which shows amplification in a species-specific manner (size of STR) in all the members of the family felidae studied here. This PCR-based, non-invasive	

				method opens a new avenue to forensic identification of big cats. © 2004 Elsevier Ireland Ltd. All rights reserved.	
WOS	10.1016/j.forsciint.2004.01.015	Novel microsatellite (STR) marker for forensic identification of big cats in India	Singh, A; Gaur, A; Shailaja, K; Bala, BS; Singh, L	India is the home to five of the eight majestic big cats of the world. The three major big cats namely, lion, tiger, and leopard are listed in the Schedule I of the Indian Wildlife Protection Act, 1972. Apart from the severe loss of the habitat, these are continuously facing the danger of extinction mainly due to poaching and hunting for their body parts, which are being greatly valued by apothecaries marketing traditional Chinese medicines. With the advent of polymerase chain reaction (PCR), DNA-based markers have emerged as major tools in the arena of wildlife forensics. Microsatellites (short tandem repeats, STRs) are markers of choice because of their polymorphic and co-dominant nature. These strictly follow the Mendelian inheritance and are highly	Cat-related

				reproducible. We have identified a new microsatellite (STR) locus Ple 46, which shows amplification in a species-specific manner (size of STR) in all the members of the family felidae studied here. This PCR-based, non-invasive method opens a new avenue to forensic identification of big cats. (C) 2004 Elsevier Ireland Ltd. All rights reserved.	
SCOPUS	222372	Populations and activity patterns of clouded leopards and marbled cats in Dampa Tiger Reserve, India	Singh, Priya (58728828800); Macdonald, David W. (7401463172)	The rapidly declining tropical forests of Asia support a diversity of felid species, many of which are rare and little known. We used camera traps in Dampa Tiger Reserve (TR), Mizoram, northeastern India, to estimate population density and describe activity patterns of 2 rare felids, the clouded leopard (Neofelis nebulosa) and marbled cat (Pardofelis marmorata). With a survey effort of 4,962 trap nights, we obtained 84 photo-captures of clouded leopards and 36 of marbled cats. We used spatially explicit capture-recapture methods to	Cat-related

				estimate population densities of both species. Using the Bayesian approach implemented in SPACECAP, we derived estimates of 5.14 (± 1.80 SD)/100 km 2 for clouded leopards and 5.03 (± 2.07 SD)/100 km 2 for marbled cats. Using camera-trap images, we compared diel activity patterns and activity overlaps for these 2 rare felids, together with 3 other sympatric carnivores, by estimating a coefficient of overlap between species. Among felids, clouded leopards and golden cats (Catopuma temminckii) displayed the highest overlap in activity, whereas marbled cats and leopard cats (Prionailurus bengalensis) showed the lowest, with marbled cats being primarily diurnal and leopard cats nocturnal. Our study provides the first density estimates from continental Southeast Asia for marbled cats and one of the highest recorded densities for clouded leopards. These results are of special	

				significance since Dampa sustains an ecosystem that has in recent times undergone near extirpation of large predators. © 2017 American Society of Mammalogists.	
SCOPUS	13835769	Gastrointestinal parasites of cats in the Middle East (2000−2023): A literature review	Soroushianfar M.; Sadr S.; Sazmand A.; Dianaty S.; Khedri J.; Schuster R.K.; Borji H.	Background/objective: Gastrointestinal parasites in stray cats are of significant clinical and zoonotic importance and pose a potential threat to public health. Hence, the current review has practical implications as it aims to present the diversity and the prevalence of cat parasites in Middle Eastern countries. Methods: The research process was rigorous and thorough, spanning from 2000 to 2023. Data collection and analysis were conducted utilizing multiple international and national databases, including Google Scholar, Scopus, PubMed, and Irandoc. This meticulous approach ensures the reliability and validity of the findings, providing a solid foundation for further research and preventive	Cat-related

				measures. Results: A total of 159 articles were collected and comprehensively reviewed. Among these, 85 articles were from Iran, 25 from Türkiye, 20 from Iraq, 11 from Egypt, five from Qatar, three from Cyprus, three from Kuwait, and one each from the UAE, Saudi Arabia, Syria, Lebanon, Jordan, Israel, and Palestine. Toxocara cati was the most prevalent nematode, exhibiting the highest prevalence in Iran, ranging from 8% to 90%, followed by Egypt, with a range of 8.23% to 58.7%. In comparison, the lowest prevalence was recorded in Qatar at 8%. Hydatigera taeniaeformis, a common cestode in the feline, showed the highest prevalence in Qatar, with a range of 73.6% to 75.8%, while the lowest was in Kuwait at 0.8%. In Iran, the prevalence ranged from 0.9% to 64.7%. Toxoplasma gondii, a cosmopolitan protozoan, showcased the highest prevalence in Lebanon at 78.1% and	

				the lowest in the UAE at 0.8%. In Iran, the prevalence ranged between 1.2% and 90%. Additionally, noteworthy patterns regarding the distribution and potential risk factors associated with these parasites were observed. While gender variations were not significant, environmental conditions such as high humidity and absence of sunlight exposure, along with the age and behavior of cats, were among the risk factors associated with the prevalence of parasites. Conclusion: The prevalence of parasites among stray cats in Iran and other Middle Eastern countries remains notably high. This emphasizes the urgent need for a series of control and preventive measures. Implementing effective anti-parasitic strategies and emphasizing the development of multiple diagnostic methods are essential. © 2024 Elsevier B.V.	
SCOPUS	13434152	Camera-Trapping Two Felid Species: Monitoring Eurasian	Soyumert A.	Documenting reliable and concrete data makes	Cat-related

		Lynx (Lynx lynx) and Wildcat (Felis silvestris) Populations in Mixed Temperate Forest Ecosystems		camera-trapping an appropriate tool for many secretive large mammals. Camera-trapping has been widely used for detecting the large mammal fauna of Turkey. However, systematic surveys and comprehensive assessments are still not sufficient for assessing wildlife populations in the country. In the present study, populations of Eurasian lynx (Lynx lynx) and wildcat (Felis silvestris) were monitored by systematic camera-trapping, and their co-existence was investigated with 70 camera-trap stations between 2014-2018 in northern Anatolia. Daily activity patterns and kernel density estimation were used to analyse and compare temporal patterns of target species. High temporal overlap (?4: 0.81) for Eurasian lynx and wildcat at the same study sites, and no alteration in wildcat activity pattern in the presence of Eurasian lynx, suggest that these	

				two felid species can co-exist in the same habitats using the same period of the day. Domestic cat (Felis catus) was detected at 52.5% of the wildcat-positive stations indicating that there is hybridisation threat for the wildcat population in the region. The study reveals that systematic camera-trapping surveys are an applicable method for monitoring cryptic mammalian carnivores and provide useful insights into the threats on wildlife populations like hybridisation. © The Mammal Society of Japan.	
SCOPUS	20762615	The Ecological Roles of Medium and Small Carnivores in the Terrestrial Animal Community in Liancheng National Nature Reserve, China	Su, Tengwei (57191093245); Li, Qian (58029179400); Wang, Xiaojuan (58029855400); Cui, Guofa (16315738500); Man, Zihong (58029855500); Li, Wentao (58029855600); Zhao, Minyan (57183401200)	It is vitally important to understand the ecological roles of medium and small carnivores in the context of the massive decline in the number of large carnivores around the world. Based on a spatial association network of terrestrial birds and mammals, this study analyzed the ecological roles of medium and small carnivores in the community in Liancheng National Nature Reserve.	Predation-related

				From October 2019 to June 2020, we obtained 3559 independent detections of 20 terrestrial birds and mammals from 112 camera traps. There are seven species that are medium and small carnivores present in the study area, including red fox (Vulpes vulpes), leopard cat (Prionailurus bengalensis), Chinese mountain cat (Felis bieti), stone marten (Martes foina), Asian badger (Meles leucurus), Siberian weasel (Mustela sibirica) and mountain weasel (Mustela altaica). By calculating the Phi coefficient of all species pairs, a spatial association network composed of twelve species was constructed. We analyzed the characterization of spatial associations by the Shannon–Wiener index and Lambda statistic. The results showed that: (1) the status of the network reflects the changes of community composition and structure after the decline in large carnivores and other	

				species; (2) with the exception of the Chinese mountain cat and stone marten, the other five medium and small carnivores were located in the network, which played an important role in the complexity of the network and the maintenance of the community; (3) the medium and small carnivores could not take the place of the large carnivores in order to control the population of herbivores, such as Siberian roe deer (Capreolus pygargus) and Himalayan marmot (Marmota himalayana). The results of this study provide guidance for determining the direction and focus of conservation efforts. © 2022 by the authors.	
J-Stage+CiNii		Nest Structure and Breeding Population Estimation of Streaked Shearwaters Calonectris leucomelas on Nakanokamishima	Takashi Yamamoto, Hiroyoshi Kohno, Akira Mizutani, Ken Yoda	We estimated the breeding population of Streaked Shearwaters Calonectris leucomelas, and investigated their nest burrow structures on Nakanokamishima. Streaked Shearwater nest burrows were found throughout the survey area, and at highest	Predation-related

				densities in flat or gentle sloping areas. The nest burrow density was 0.64±0.22 nest/m2 for the high nest-den- sity area, 0.19±0.15 nest/m2 for the thicket area, and 0.10±0.10 nest/m2 for the low nest-den- sity area. Brooding nest density was 0.13±0.10 nest/m2 for the high nest-density area, 0.01± 0.03 nest/m2 for the thicket area, and 0.01±0.03 nest/m2 for the low nest-density area. We multiplied the 3D area by the nest burrow or brooding nest density in each area type, and the total numbers of nest burrow and brooding nest on Nakanokamishima were estimated to be 18,853 and 2,783 nests, respectively, and breeders as 5,566 birds. Entrances of nest burrows of Streaked Shearwater positioned in slope areas tended to be directed to downward of the slope, and entrances of those in flat places tended to be directed to the nearest coast. We found two types of nest structure: straight or curved(or hooked)types. The length	

				of burrows was 75± 22 cm for the straight type and 99±20 cm for the curved type.	
SCOPUS	1229845X	Feline vocal communication	Tavernier, Chloé (57214670074); Ahmed, Sohail (57209193124); Houpt, Katherine Albro (7006392256); Yeon, Seong Chan (8953995900)	Cat vocalizes to communicate with another and express their internal states. The vocal repertoire of the cat is wide and up to 21 different vocalizations have been described in the literatures. But it is more than probable that the repertoire contains more types of vocalizations. An ethogram was created in this paper describing the actual known vocalisations of the domestic cat based on an auditory classification. However, the audiogram allows also a visual classification, which can increase the accuracy of vocalization differentiation. The classification can be risky as it is sometimes unclear if different types of vocalizations are produced in different environments or if a unique type of vocalization is used with variation in the acoustic parameters. As an	Predation -related

				example, isolation calls produced by kittens differ depending on the context. The environment has an important impact on the vocal behaviour and thus feral cats and pet cats vocalize differently. Pet cats are thus able to create an efficient communication with humans thanks to the flexibility of vocalisation behaviours. This review allowed us to create a simple model of the cat vocal repertoire. © 2020 The Korean Society of Veterinary Science.	
SCOPUS	21650497	A Cross-Sectional Study of Companion Animal-Derived Multidrug-Resistant Escherichia coli in Hangzhou, China	Teng, Lin (58737313100); Feng, Mengyao (57695128300); Liao, Sihao (57420977200); Zheng, Zhijie (57936559000); Jia, Chenghao (57420977100); Zhou, Xin (57205346679); Nambiar, Reshma B. (57002877700); Ma, Zhengxin (57695615100); Yue, Min (55249653200)	Antimicrobial resistance poses a challenge to global public health, and companion animals could serve as the reservoir for antimicrobial-resistant bacteria. However, the prevalence of antimicrobial-resistant bacteria, especially multidrug-resistant (MDR) bacteria, and the associated risk factors from companion animals are partially understood. Here, we aim to investigate the prevalence of MDR Escherichia coli, as an indicator bacterium, in	Predation-related

				pet cats and dogs in Hangzhou, China, and evaluate the factors affecting the prevalence of MDR E. coli. The proportion of pets carrying MDR E. coli was 35.77% (49/137), i.e., 40.96% (34/83) for dogs and 27.28% (15/ 54) for cats. Isolates resistant to trimethoprim-sulfamethoxazole (49.40% and 44.44%), amoxicillin-clavulanic acid (42.17% and 38.89%), and nalidixic acid (40.96% and 35.19%) were the most prevalent in dogs and cats. Interestingly, comparable prevalence of MDR E. coli was observed in pet dogs and cats regardless of the health condition and the history of antibiotic use. Genetic diversity analysis indicates a total of 86 sequencing types (23 clonal complexes), with ST12 being the most dominant. Further genomic investigation of a carbapenem-resistant E. coli ST410 isolate reveals abundant antimicrobial-resistance genes and a plasmid-borne	

				carbapenemase gene (NDM-5) flanked by insertion sequences of IS91 and IS31, suggesting the plasmid and insertion sequences may be involved in carbapenem-resistance dissemination. These data show that companion animal-derived MDR bacteria could threaten public health, and further regulation and supervision of antimicrobial use in pet clinics should be established in China. IMPORTANCE MDR Escherichia coli are considered a global threat because of the decreasing options for antimicrobial therapy. Companion animals could be a reservoir of MDR E. coli, and the numbers of pets and households owning pets in China are booming. However, the prevalence and risk factors of MDR E. coli carriage in Chinese pets were rarely studied. Here, we investigated the prevalence of MDR E. coli in pets in Hangzhou, one of the leading cities with the most established	

				pet market in China, and explored the factors that affected the prevalence. Our findings showed high prevalences of MDR E. coli in pet dogs and cats regardless of their health condition and the history of antibiotic use, suggesting their potential role of public health risk. A call-to-action for improved regulation of antimicrobial use in companion animal is needed in China. Copyright © 2023 Teng et al.	
WOS	10.1017/S0030605317001491	The Vulnerable fishing cat Prionailurus viverrinus and other globally threatened species in Cambodia's coastal mangroves	Thaung, R; Muñoz, VH; Holden, J; Willcox, D; Souter, NJ	The Vulnerable fishing cat Prionailurus viverrinus faces a perilous future in South-east Asia. It was last sighted in Cambodia in 2003. We deployed 16 camera traps at four sites in southern Cambodia during January-May 2015 to determine if the fishing cat was still present in the country. Eight photograph/video captures of fishing cats were recorded from the mangroves in Peam Krasop Wildlife Sanctuary and one from Ream National Park, but there were no records	Cat-related

				from Botum Sakor National Park or Prey Nup. A number of other globally threatened species were also photographed in Peam Krasop Wildlife Sanctuary: the Sunda pangolin Manis javanica, the hog deer Axis porcinus and the large-spotted civet Viverra megaspila. We learnt of the killing of an alleged fishing cat at the Sanctuary in July 2015 in retaliation for raiding fishing nets. Illegal hunting and capture of fishing cats for the wildlife trade were reported by local informants at all sites. We provide photographic and video evidence of the fishing cats and highlight the importance of Cambodia's mangroves for threatened species conservation.	
SCOPUS	9212973	Climate change and landscape fragmentation jeopardize the population viability of the Siberian tiger (Panthera tigris altaica)	Tian, Yu (57198239423); Wu, Jianguo (54080586800); Wang, Tianming (55709746500); Ge, Jianping (55443791600)	The Amur tiger, a flagship species of the boreal forest ecosystem in Russian Far East and northeastern China, has declined dramatically in population and geographic distribution due to human caused	Cat-related

				habitat fragmentation and poaching over the past century. The fate of this largest feline species will also be influenced by the worsening impacts of climate change. In this paper we assess the possible effects of climate change (three scenarios from the 2007 IPCC Report) on the Amur tiger by integrating species distribution modeling (SDM) and population viability analysis (PVA). We projected the potential and realized suitable habitat distributions to examine the impacts from anthropogenic factors, and evaluated the changes of suitable habitat and extinction risk for 100 years under climate change. The realized suitable habitat was projected to be more severely fragmented than the potential suitable habitat because of human-related factors. The potential suitable habitat would expand northward under all climate change scenarios considered. However, the tiger population would	

				suffer the largest decline and highest extinction risk in the next 100 years under the worst climate change scenario (A1B) even though the size of potential habitat would be greatest. Under climate change, the tiger population could persist for the next century only if the size and quality of current habitat patches would remain intact. In addition, our study demonstrated that using SDM alone could grossly overestimate the geographic distribution of the Amur tiger, and that coupling SDM and PVA could provide important insights into conservation planning to mitigate the effects of climate change. © 2014 Springer Science+Business Media Dordrecht.	
WOS	10.3390/ani13020234	Fine-Scaled Selection of Resting and Hunting Habitat by Leopard Cats (Prionailurus bengalensis) in a Rural Human-Dominated Landscape in Taiwan	van der Meer, E; Dullemont, H; Wang, CH; Zhang, JW; Lin, JL; Pei, KJC; Lai, YC	Simple Summary Presently, Taiwan's endangered leopard cat mostly occurs in unprotected, rural, human-dominated landscapes. To survive within these landscapes, the species needs suitable habitat for nighttime	Cat-related

				hunting but also safe refuge for resting during daytime hours when human activity, and herewith human-related threat, peaks. Although important for the species' conservation, little is known about the characteristics of the leopard cat's resting habitat. In this study, we tracked seven VHF-collared leopard cats. Every day we determined where these leopard cats rested, and every ten days we followed a leopard cat throughout the night and recorded its locations at 30 min intervals. We assessed land use at nighttime locations and land use and fine-scaled vegetation characteristics at resting sites and determined whether leopard cats selected certain habitats or simply used them according to availability. The leopard cats in our study seemed to use a proactive strategy to avoid humans by selecting natural habitats for hunting and resting and avoiding areas with high levels of human activity. Resting	

				sites were preferably situated in natural habitats with little visibility (<2 m), shrubs, reed and stones, away from orchards, buildings and roads. This information will assist in identifying and conserving suitable resting habitats for leopard cats. Wildlife is increasingly forced to live in close proximity to humans, resulting in human-wildlife conflict and anthropogenic mortality. Carnivores persisting in human-dominated landscapes respond to anthropogenic threats through fine-scaled spatial and temporal behavioral adjustments. Although crucial for conservation, quantitative information on these adjustments is scarce. Taiwan's endangered leopard cat occurs in rural human-dominated landscapes with a high anthropogenic mortality risk. To survive, the nocturnal leopard cat needs suitable habitats for foraging and safe refuge for resting during	

				daytime hours when human activity peaks. In this study, we tracked seven VHF-collared leopard cats. To determine habitat selection patterns, we compared land use at nighttime locations and daytime resting sites with random points and fine-scaled vegetation characteristics at daytime resting sites with random points. Leopard cats selected natural habitats for nighttime hunting and avoided manmade and, to a lesser extent, agricultural habitats or used them according to availability. For daytime resting, leopard cats selected natural habitats and, to a lesser extent semi-natural habitats, such as unused land and abandoned orchards. Resting sites were preferentially situated in natural habitats, with little visibility (<2 m), shrubs, reed and stones, away from areas with high levels of human activity. This suggests leopard cats use a proactive strategy to avoid human encounters,	

				which was supported by the reduced temporal overlap with humans and domestic dogs on agricultural land. Resting sites were placed ca. 1 km apart, 12.9 +/- 0.3 m (mean +/- SE) from the patch's edges, in patches with a size of 1.21 +/- 0.04 ha (mean +/- SE). Our results will assist in identifying and preserving suitable resting habitats to support leopard cat conservation.	
WOS	10.1016/j.bbr.2026.116073	Male paternal exposure to predatory risk during adolescence alters offspring antipredator behavior and basal HPA axis activity in Brandt's voles	Wang P.; Zhou T.; Gu Y.; Lan X.; Wei W.; Wu R.	Adolescent stress not only exerts enduring effects on individual behavior and physiology, but also shapes offspring phenotypes through mechanisms of transgenerational inheritance. However, the transgenerational effects of predation risk, an important ecological factor, remain poorly characterized in wild, social rodents. Using male Brandt's voles (Lasiopodomys brandtii) as a model, this study systematically investigated the transgenerational effect of adolescent exposure to cat urine, rabbit urine (as	Unable to access data

				a non-predator stimulus), and distilled water (as a control) for 60 min daily over 18 consecutive days on the behavioral and physiological traits of future offspring. Our data showed that while male paternal adolescent cat odor (CO) exposure did not significantly alter overall parental investment by either parent, it induced significant phenotypic changes in offspring. These included an increased female ratio at weaning and reduced post-weaning weight gain. Furthermore, adolescent offspring exhibited decreased locomotor activity in the open field test, while adult offspring displayed heightened vigilant rearing and reduced head-out behavior when confronted with CO exposure. These behavioral and developmental alterations were accompanied by elevated serum levels of adrenocorticotropic hormone and corticosterone, suggesting enhanced	

				basal activity of the hypothalamic-pituitary-adrenal axis. These findings demonstrate that male paternal experience with predation risk during adolescence can transgenerationally regulate the sex ratio, growth and development, stress response, and antipredator strategies of future offspring, independent of alterations in parental investment in wild, social rodents. This study elucidates the unique role of the paternal lineage in the transgenerational inheritance of early adversity and provides further experimental evidence for understanding how environmental stress drives adaptive phenotypic transmission across generations. © 2026 Elsevier B.V.	
SCOPUS	9212973	Amur tigers and leopards returning to China: direct evidence and a landscape conservation plan	Wang, Tianming (55709746500); Feng, Limin (36886437200); Mou, Pu (6701853256); Wu, Jianguo (54080586800); Smith, James L. D.	Context: The Amur tiger and leopard, once roaming over the Eurasian continent, are now endangered and confined to the Sikhote-Alin Mountains, Russia—a landscape that has been increasingly	Cat-related

			(36101769900); Xiao, Wenhong (56911601600); Yang, Haitao (56911196900); Dou, Hailong (56585453800); Zhao, Xiaodan (56911242700); Cheng, Yanchao (57204174134); Zhou, Bo (56911482200); Wu, Hongyan (56911223500); Zhang, Li (35188410800); Tian, Yu (57198239423); Guo, Qingxi (22634161200); Kou, Xiaojun (23004746200); Han, Xuemei (56438396700); Miquelle, Dale G. (6603481067); Oliver, Chadwick D. (7202581610); Xu, Rumei (7402814144); Ge, Jianping (55443791600)	fragmented due to human activities. The ultimate fate of these big cats depends on whether they can resettle in their previous main historical range in NE China. Recent sightings of these animals along the China–Russia border have aroused new hope, but direct evidence is lacking. Objectives: The main objectives of our study were (1) to determine the abundance and spatiotemporal patterns of tigers, leopards, and primary prey; (2) to investigate factors influencing the resettlement of the two big cats; and (3) to propose a landscape-scale conservation plan to secure the long-term sustainability of the Amur tiger and leopard. Methods: We monitored the two felids, their prey, and human activities, with 380 camera-trap stations, for a total of 175,127 trap days and over an area of 6000 km2 in NE China. We used the constraint line method to characterize cattle grazing and human	

				influences on tigers, leopards, and their prey species. Results: Our results show that, unexpectedly, at least 26 tigers and 42 leopards are present within China, which are confined primarily to a narrow area along the border with Russia. We have further identified that cattle grazing and human disturbances are the key hurdles to the dispersal of the tigers and leopards farther into China where suitable habitat is potentially available. Conclusions: Amur tigers and leopards are returning to China, indeed, but their long-term resettlement is not likely without active and timely conservation efforts on landscape and regional scales. To overcome the hurdles to the resettlement of tigers and leopards in China, we propose a "Tiger and Leopard Resettlement Program" that will engage the government, local communities, and researchers, so that the long-term sustainability of the Amur tigers and	

				leopards can be ensured. © 2015, Springer Science+Business Media Dordrecht.	
SCOPUS	13486160	Factors affecting the distribution of the leopard cat prionailurus bengalensis on east asian islands	Watanabe, Shinichi (59035935700)	Members of the Felidae, being found at the top of the trophic hierarchy in an ecosystem, usually require extremely large habitat ranges. Thus, most cat species are found only on continents or large islands. The leopard cat Prionailurus bengalensis, the most widespread species of East Asian cat, is an exception to this rule, occurring on several small islands as well as larger islands and the Asian continent. Using published data for 38 East Asian islands, I evaluated the effects of geographical and biological factors on the persistence of the leopard cat population on an island by using regression analyses. The results suggested that the probability of existence of this cat species on an island increased with increasing species richness of potential prey, or with decreasing species richness of	Cat-related

				potential competitors. Geographical variation in the feeding habits of the leopard cat was also examined to clarify its ecological adaptation to different environments. The leopard cat chiefly preys on rodents but occasionally also feeds on other kinds of animals depending on region. On small islands with a small number of carnivore species, the cat frequently feeds on non-mammalian prey. This suggests that the cat potentially utilizes a variety of food items. The diet width of the leopard cat may be being determined by availability of prey items and intensity of interspecific competition. © the Mammalogical Society of Japan.	
WOS	10.1371/journal.pone.0185996	A roaring trade? The legal trade in Panthera leo bones from Africa to East-Southeast Asia	Williams, VL; Loveridge, AJ; Newton, DJ; Macdonald, DW	The African lion is the only big cat listed on CITES Appendix II, and the only one for which international commercial trade is legal under CITES. The trade in lion body parts, and especially the contentious trade in bones from South Africa to Asia, has	Cat-related

				raised concerns spanning continents and cultures. Debates were amplified at the 2016 CITES Conference of the Parties (CoP17) when a proposal to up-list lions to Appendix I was not supported and a compromise to keep them on Appendix II, with a bone trade quota for South Africa, was reached instead. CoP17 underscored a need for further information on the lion bone trade and the consequences for lions across the continent. Legal international trade in bones to Asia, allegedly to supply the substitute 'tiger bone' market, began in South Africa in February 2008 when the first CITES permits were issued. It was initially unclear the degree to which bones were sourced from captive-origin lions, and whether trade was a threat to wild lion populations. Our original assessment of the legal CITES-permitted lion bone trade from South Africa to East-Southeast Asia was for	

				the period 2008-2011 (published 2015). In this paper, we consolidate new information that has become available for 2012-2016, including CITES reports from other African countries, and data on actual exports for three years to 2016 supplied by a freight forwarding company. Thus, we update the figures on the legal trade in lion bones from Africa to East-Southeast Asia in the period 2008-2016. We also contextualise the basis for global concerns by reviewing the history of the trade and its relation to tigers, poaching and wildlife trafficking. CITES permits issued to export bones escalated from +/- 314y(-1) skeletons from 2008-2011, to +/- 1312y(-1) skeletons from 2013-2015. South Africa was the only legal exporter of bones to Asia until 2013 when Namibia issued permits to export skeletons to Vietnam. While CITES permits to export +/- 5363 skeletons from Africa to Asia from 2008-2015 were issued	

				(99.1% from South Africa; 0.7% from Namibia) (51% for Laos), actual exports were less than stated on the permits. However, information on actual exports from 2014-2016 indicated that >3400 skeletons were exported in that period. In total, >6000 skeletons weighing no less than 70 tonnes have been shipped to East-Southeast Asia since 2008. Since few wild lions are hunted and poached within South African protected areas, skeletons for the legal trade appear to be derived from captive bred lions. However, confirmation of a 116kg shipment from Uganda to Laos, and reports of lion poaching in neighbouring countries, indicate that urgent proactive monitoring and evaluation of the legal and illegal trade is necessary in African lion range states where vulnerable wild lion populations are likely to be adversely affected.	
SCOPUS	19326203	Modelling the species distribution of flat-headed	Wilting, Andreas (15133557500);	Background: The flat-headed cat (Prionailurus	Cat-related

		cats (Prionailurus planiceps), an endangered South-East Asian small felid	Cord, Anna (34881399900); Hearn, Andrew J. (37001484600); Hesse, Deike (58376429400); Mohamed, Azlan (56666657200); Traeholdt, Carl (37002406100); Cheyne, Susan M. (23396245300); Sunarto, Sunarto (58799348900); Jayasilan, Mohd-Azlan (55666218000); Ross, Joanna (55604400300); Shapiro, Aurelie C. (8624044500); Sebastian, Anthony (37001971700); Dech, Stefan (6701611146); Breitenmoser, Christine (35274253100); Sanderson, Jim (7202371268); Duckworth, J.W. (7006663228); Hofer, Heribert (7101860243)	planiceps) is one of the world's least known, highly threatened felids with a distribution restricted to tropical lowland rainforests in Peninsular Thailand/Malaysia, Borneo and Sumatra. Throughout its geographic range large-scale anthropogenic transformation processes, including the pollution of fresh-water river systems and landscape fragmentation, raise concerns regarding its conservation status. Despite an increasing number of cameratrapping field surveys for carnivores in South-East Asia during the past two decades, few of these studies recorded the flatheaded cat. Methodology/Principal Findings: In this study, we designed a predictive species distribution model using the Maximum Entropy (MaxEnt) algorithm to reassess the potential current distribution and conservation status of the flat-headed cat. Eighty-eight independent species	

				occurrence records were gathered from field surveys, literature records, and museum collections. These current and historical records were analysed in relation to bioclimatic variables (WorldClim), altitude (SRTM) and minimum distance to larger water resources (Digital Chart of the World). Distance to water was identified as the key predictor for the occurrence of flat-headed cats (>50% explanation). In addition, we used different land cover maps (GLC2000, GlobCover and SarVision LLC for Borneo), information on protected areas and regional human population density data to extract suitable habitats from the potential distribution predicted by the MaxEnt model. Between 54% and 68% of suitable habitat has already been converted to unsuitable land cover types (e.g. croplands, plantations), and only between 10% and 20% of suitable land cover is categorised as fully protected according to	

				the IUCN criteria. The remaining habitats are highly fragmented and only a few larger forest patches remain. Conclusion/Significance: Based on our findings, we recommend that future conservation efforts for the flat-headed cat should focus on the identified remaining key localities and be implemented through a continuous dialogue between local stakeholders, conservationists and scientists to ensure its long-term survival. The flat-headed cat can serve as a flagship species for the protection of several other endangered species associated with the threatened tropical lowland forests and surface fresh-water sources in this region. © 2010 Wilting et al.	
SCOPUS	19326203	Combining vertebrate mitochondrial 12S rRNA gene sequencing and shotgun metagenomic sequencing to investigate the diet of the leopard cat (Prionailurus bengalensis) in Korea	Woo, Cheolwoon (57192187621); Kumari, Priyanka (56425574200); Eo, Kyung Yeon (26631541100); Lee, Woo-Shin (7407088617); Kimura, Junpei	The leopard cat (Prionailurus bengalensis), an endangered species in South Korea, is a small feline widely distributed in Asia. Here, we investigated the diet of leopard cats in the inland	Cat-related

			(56669308300); Yamamoto, Naomichi (7403608251)	areas of Korea by examining their fecal contents using vertebrate mitochondrial 12S rRNA gene sequencing and shotgun metagenomic sequencing. Shotgun metagenomic sequencing revealed that the feces were rich in DNA not only of vertebrates but also of arthropods and plants, but care should be taken when using shotgun metagenomic sequencing to identify vertebrates at low taxonomic levels (e.g., genus level), as it was often erroneous. Meanwhile, vertebrate mitochondrial 12S rRNA gene sequencing was found to be accurate in the genus-level identification, as the genera identified were consistent with the Korean fauna. We found that small mammals such as murids were their main prey. By using these two sequencing methods in combination, this study demonstrated that accurate information about the overall dietary content and vertebrate prey of leopard cats	

				could be obtained. We expect that the continued community efforts to expand the genome database of wildlife, including vertebrates, will alleviate the problem of erroneous identification of prey at low taxonomic levels by shotgun metagenomic sequencing in the near future. © 2023 Woo et al. This is an open access article distributed under the terms of the Creative Commons Attribution License, which permits unrestricted use, distribution, and reproduction in any medium, provided the original author and source are credited.	
SCOPUS	222372	The truth about cats and dogs: Assessment of apex- and mesopredator diets improves with reduced observer uncertainty	Wysong, Michael L. (57204322114); Tulloch, Ayesha I. T. (15752345400); Valentine, Leonie E. (14072178800); Hobbs, Richard J. (7202267633); Morris, Keith (7202939036); Ritchie, Euan G. (57205357686)	Dietary (scat) analysis is a key tool for assessing the potential effects of predators on prey and for comparing resource use between predators, information that is crucial for effective wildlife management. However, misidentification of the species from which scats originate could result in inaccurate conclusions regarding predator-prey interactions and their	Predation -related

				consequences for ecosystems, which may ultimately compromise conservation and management actions. To address this issue, we developed a framework for decision-making in the face of uncertain scat species origin by incorporating field, laboratory, and molecular identification techniques. We used the framework to examine the diets of two predators, a native apex predator (dingo, Canis lupus dingo) and an invasive mesopredator (feral cat, Felis catus), from 696 field-collected scats in the arid zone of Australia. We examined how uncertainty regarding scat species origin changed perceptions of the nature of the relationship between coexisting predators and their prey. The extent of dietary overlap between dingoes and cats varied with the method used to identify scat species origin. Dietary overlap assessed by laboratory identifications was twice as high as when	

				uncertainty in scat species origin was resolved through our decision framework. If uncertainty in scat species origin is not resolved in dietary studies, practitioners and decision-makers relying on this information run the risk of making misinformed conclusions regarding the ecological function of predators (including potential impacts on threatened species), which could have perverse outcomes if the wrong predators are targeted for management. With uncertainty in scat species origin resolved through our decision framework, a low level of dietary overlap between the two predators was demonstrated, and medium-sized mammals most threatened with extinction were shown to be more at risk of impact from feral cat than from dingo depredations. © 2019 American Society of Mammalogists.	
SCOPUS	20452322	Molecular dietary analysis of two sympatric felids in the Mountains of Southwest	Xiong, Mengyin (57190750179); Wang, Dajun (7407070322); Bu,	Dietary information is lacking in most of small to mid-sized carnivores due to their elusive	Cat-related

		China biodiversity hotspot and conservation implications	Hongliang (56921501000); Shao, Xinning (57190747802); Zhang, Dan (56920843000); Li, Sheng (56438375200); Wang, Rongjiang (56128402100); Yao, Meng (36852632500)	predatory behaviour and versatile feeding habits. The leopard cat (LPC; Prionailurus bengalensis) and the Asiatic golden cat (AGC; Catopuma temminckii) are two important yet increasingly endangered carnivore species in the temperate mountain forest ecosystem in Southwest China, a global biodiversity hotspot and a significant reservoir of China's endemic species. We investigated the vertebrate prey of the two sympatric felids using faecal DNA and a next-generation sequencing (NGS)/metabarcoding approach. Forty vertebrate prey taxa were identified from 93 LPC and 10 AGC faecal samples; 37 taxa were found in the LPC diet, and 20 were detected in the AGC diet. Prey included 27 mammalian taxa, 11 birds, one lizard and one fish, with 73% (29/40) of the taxa assigned to the species level. Rodents and pikas were the most dominant LPC prey categories,	

				whereas rodents, pheasant, fowl and ungulates were the main AGC prey. We also analysed the seasonal and altitudinal variations in the LPC diet. Our results provide the most comprehensive dietary data for these felids and valuable information for their conservation planning. © 2017 The Author(s).	
SCOPUS	16746767	Winter habitat use of snow leopards in tomur national nature reserve of Xinjiang, northwest China	Xu, Feng (56412307700); Ma, Ming (36573830600); Yang, WeiKang (14057440400); Blank, David (37116395000); Wu, YiQun (56093260700); McCarthy, Thomas (7201694358); Munkhtsog, Bariusha (25422302800)	Snow leopards are one of the least known large cats, the population of which has dramatically decreased. Their habitat loss was considered the main reason for the decline during the last decade, but their habitat preferences are still not well known. In this paper, we studied the winter habitat preferences of snow leopards in the Tomur National Nature Reserve (TNNR) in Northwest China during 2004-2005. We used sign surveys and transects to study the winter habitat selection of snow leopards. The results indicated that snow leopards showed a preference for habitat	Cat-related

				variabilities in slope aspect, vegetation cover, dominant topographical features, landform ruggedness and grazing status. We found that prey availability and dominant topographical features were the most important factors that determined the winter habitat selection of snow leopards. Our results supported the idea that the habitat preferences of snow leopards are a tradeoff between suitable habitat features and avoidance of potential human interactions.	
SCOPUS	20762615	Trends in Animal Shelter Management, Adoption, and Animal Death in Taiwan from 2012 to 2020	Yan, Tzu-Yun (58244769400); Teng, Kendy Tzu-yun (57194497845)	This current study investigated the trends in public animal shelter intakes and outcomes and the workload of shelter veterinarians in Taiwan from 2012 to 2020 and reports spatial, temporal, and socioeconomic factors associated with these trends. Information about the public animal shelter management of dogs and cats from all counties of Taiwan between 2012 and 2020 was acquired from the National Animal Shelter Management System of	Predation-related

				the Council of Agriculture Executive Yuan in Taiwan. Ridge regression followed by multivariable linear regression was conducted to examine the risk factors for animal intakes, outcomes, the number of adopted animals, and the estimated veterinary workload in public animal shelters. The intakes and outcomes of shelter animals significantly decreased over time. Euthanasia, which was performed in the shelters, was positively associated with shelter animal intakes and outcomes as it resulted in animal outcomes and thus facilitated the flow of animals in the shelters. Adoption and trap–neuter–vaccination–return, in replacement of euthanasia, became the main reasons for animal outcomes, and with every increase in human fertility rate, the monthly number of adopted animals over the number of animals entering shelters increased by	

				1.10% (95% CI: 0.21 to 2.00). The veterinary workload in the shelters of two counties exceeded what is regulated by law (i.e., 100 animals per veterinarian) in 2018 and increased to six counties in 2020. This current study reported important trends in the management of public animal shelters in Taiwan, the increasing workload of shelter veterinarians, and factors associated with these trends. It built the epidemiological foundation for future research on methods of improving shelter management and work conditions for shelter staff. © 2023 by the authors.	
SCOPUS	16165047	Spatiotemporal patterns of Amur leopards in northeast China: Influence of tigers, prey, and humans	Yang, Haitao (56911196900); Zhao, Xiaodan (56911242700); Han, Boyu (57188622741); Wang, Tianming (55709746500); Mou, Pu (6701853256); Ge, Jianping (55443791600); Feng, Limin (36886437200)	The Amur leopard Panthera pardus orientalis is one of the most endangered cat subspecies in the world. The rare leopard is sympatric with Amur tiger Panthera tigris altaica and their prey in human dominated landscape. To conserve the felid species, it is important to understand the activity patterns of	Cat-related

				Amur leopards, including its interactions with Amur tigers, prey, and human activities. We used a data set from 163 camera traps to quantify the spatial-temporal overlap between Amur leopards, Amur tigers, prey species, and human disturbances (e.g., humans presence on foot, vehicles, domestic dogs, and cattle grazing) from January to December 2013 in the Hunchun Nature Reserve, NE China. Our results indicated that leopards were more active in daytime and twilight; the seasonal spatial-temporal overlaps between leopards and tigers were lower than that between leopards and their prey species. Human activities and cattle grazing could influence the spatial distribution and activity patterns of the leopards, and therefore, the conservation actions should focus on reduction of human disturbances to minimize the impacts to Amur leopard activity patterns. © 2018	

				Deutsche Gesellschaft für Säugetierkunde	
SCOPUS	20452322	Spatial and temporal relationship between native mammals and free-roaming dogs in a protected area surrounded by a metropolis	Yen, Shih-Ching (55967823500); Ju, Yu-Ten (56268952000); Shaner, Pei-Jen Lee (15129272400); Chen, Hsiang Ling (57202645627)	With rapid urbanization worldwide, anthropogenic impacts such as human settlements and invasive carnivores (dogs Canis familiaris, cats Felis catus) are altering spatial distributions and temporal activity patterns of native species. In this study, we focused on spatiotemporal responses of native mammals to anthropogenic impacts in a protected area surrounded by a large metropolis (i.e. Yangmingshan National Park inside Taipei-Keelung metropolis in northern Taiwan). We collected site use data of 11 mammal species (i.e. dogs, cats, nine native species) between 2012 and 2017 with a camera system comprising 121 camera sites. We quantified anthropogenic disturbances as distance to human settlements and activity levels of free-roaming dogs and cats. Species richness and occurrences of the native mammals increased with	Cat-related

				increasing distances to human settlements and decreasing activity level of dogs, with the latter having a stronger effect than the former. Diel activity overlap between native mammals and dogs was lower during April–July season, coinciding with the breeding season for several native mammals. In contrast, activity level of cats showed no relationships with species richness, occurrences or diel activities of the native mammals. This study demonstrated negative impacts of human settlements and free-roaming dogs on native mammal communities for protected areas in urban environments, and highlights dog activity as a major anthropogenic threat to wildlife. © 2019, The Author(s).	
SCOPUS	14726785	Predictors for presence and abundance of small mammals in households of villages endemic for commensal rodent plague in Yunnan Province, China	Yin, Jia-Xiang (25931300900); Geater, Alan (7004282902); Chongsuvivatwong, Virasakdi (7003394792); Dong, Xing-Qi	Background. Ninety-one rodent plague epidemics have occurred in Lianghe county, Yunnan Province, China, between 1990 and 2006. This study aimed to identify predictors for the presence and	Cat-related

			(56953906700); Du, Chun-Hong (15727381900); Zhong, You-Hong (15728911200); McNeil, Edward (35870443600)	abundance of small mammals in households of villages endemic for rodent plague in Lianghe county. Results. Rattus flavipectus and Suncus murinus were the two species captured in 110 households. Keeping cats decreased the number of captures of R. flavipectus by one to two thirds and the chance of reported small mammal sightings in houses by 60 to 80%. Food availability was associated with fewer captures. Keeping food in sacks decreased the small mammal captures, especially of S. murinus 4- to 8-fold. Vegetables grown around house and maize grown in the village reduced the captures of S. murinus and R. flavipectus by 73 and 45%, respectively. An outside toilet and garbage piles near the house each reduced R. flavipectus captures by 39 and 37%, respectively, while raising dogs and the presence of communal latrines in the village increased R. flavipectus captures by 76 and 110% but were	

				without detectable effect on small mammal sightings. Location adjacent to other houses increased captures 2-fold but reduced the chance of sightings to about half. In addition, raising ducks increased the chance of sighting small mammals 2.7-fold. Even after adjusting for these variables, households of the Dai had higher captures than those of the Han and other ethnic groups. Conclusion. Both species captures were reduced by availability of species-specific foods in the environment, whereas other predictors for capture of the two species differed. Other than the beneficial effect of cats, there were also discrepancies between the effects on small mammal captures and those on sightings. These differences should be considered during the implementation and interpretation of small mammal surveys. © 2008 Yin et al; licensee BioMed Central Ltd.	
Pubmed	10.1007/s10530-010-9851-3	Temporal overlaps of feral cats with prey and	Bogdan, V; Junek, T; Vymyslická, PJ	-	EA

		competitors in primary and human-altered habitats on Bohol Island, Philippines			
SCOPUS	10.56899/153.01.30	Cat’s Paw: Tracking the Home Range of Domestic Cats in Mount Makiling Forest Reserve	Tenorio F.M.B.; Fernandez D.A.P.; de Luna M.C.T.; de Guia A.P.O.; Balatibat J.B.; Baril J.A.; Aurellado M.E.B.	-	EA
Reference	0.5038/2074-1235.41.2.1036	The impact of fox and feral cat predation on the population viability of the threatened, endemic socotra cormorant on Siniya island, United Arab Emirates	Muzaffar S.B.; Benjamin S.D.; Gubiani R.E.	-	EA
WOS	10.1007/s00343-019-8025-0	Antioxidant systems of aquatic macrophytes in three life forms: a case study in Lake Erhai, China	Yuan, CB; Zhu, TS; Cao, T; Xi, YL; Zhang, XL; Ni, LY	Antioxidant systems are vital in life activities of macrophytes. Species with different life forms need to cope with distinct environments by modifying physiological characters, especially antioxidant systems. In order to find differences among life forms and consequence of lake eutrophication, we studied three antioxidant enzymes activity (superoxide dismutase SOD, ascorbate oxidase APX and catalase CAT) and total soluble phenolics (TP) content in leaves of 26 macrophyte species in September	Cat-related

				2013 in Lake Erhai, China. We found that antioxidation varied accordingly with life forms. The activities of SOD and APX in emergent macrophytes (EM) and floating-leaved macrophytes (FM) were much lower than those of submerged macrophytes (SM). On the contrary, TP content was much higher in EM and FM species. There was a negative correlation between TP and antioxidant enzyme activities (CAT and APX). The results suggested that EM and FM species rely on phenolics might to adapt to adverse environments (higher herbivores predation pressure and UV radiation intensity), while SM species more rely on antioxidant enzymes possibly due to lower demand for antioxidation and/or lack of light and inorganic C availability for phenolics synthesis. We also found FM species represent highest fitness in term of antioxidant system, which would lead to	

				overgrowth of FM species and littoral zone bogginess during lake eutrophication. Finally, it is necessary to carry out the verification experiment under the control condition in the later stage, especially for the dominant ones in eutrophic lakes, to understand the exact adaptive mechanisms of them.	
Pubmed	10.3390/ani14162301	Public Attitudes towards and Management Strategies for Community Cats in Urban China	Gu, Xuan; Wu, Di; Zhang, Zilin; Peng, Guo; Ni, Anru; Wang, Bo; Xiong, Xiufan; Liu, Yujie; Wang, Li	Managing community cats in urban China is a contentious and emerging issue, with debates centering on the most effective and humane approaches. This study aimed to investigate public attitudes towards community cats and various management strategies. A survey was conducted involving 5382 urban residents in China. Their attitudes towards the positive and negative roles of community cats in urban areas and their support for different management methods were examined, including trap-and-kill, taking no action, centralized management, and trap-neuter-return	Predation -related

				(TNR) and its variations. Results indicated that 63% of participants were willing to coexist with community cats, 71% opposed trap-and-kill, and 61% agreed or strongly agreed with the TNR method and its variations. Older residents or those with higher incomes were more likely to support coexistence with community cats. In contrast, younger or lower-income residents were more likely to support non-coexistence. Residents in first- or second-tier cities (e.g., Beijing, Hangzhou, and Jinan Cities in China) were more inclined to support trap-and-kill and less likely to support coexistence than their counterparts in fourth-tier cities (e.g., county-level cities in China). Moreover, those with lower education or incomes were more supportive of trap-and-kill and taking no action as the methods to manage community cats than those with relatively higher education or	

				incomes. Those with higher incomes held more positive attitudes towards community cats and were more supportive of TNR and its variations than their counterparts with lower incomes. Males were more inclined to support trap-and-kill and taking no action and less inclined to support centralized management and TNR than females. The implications of the findings on TNR with adoption programs in urban China are discussed. These novel findings underscore the need for targeted educational campaigns to promote humane and effective management strategies, addressing public concerns and community cats' welfare. The study's insights are critical for informing policy and improving community cat management in urban China.	
Pubmed	10.3390/ani12091141	Home Range and Activity Patterns of Free-Ranging Cats: A Case Study from a Chinese University Campus	Zhang, Zhenwei; Li, Yuhang; Ullah, Sana; Chen, Lixin; Ning, Sihan; Lu, Liangyu; Lin,	Human activities and the available resources influence the home range and activity patterns of free-ranging cats. Our	Predation -related

			Weiming; Li, Zhongqiu	objective in this study was to determine sex and breeding season vs. non-breeding season home range size, as well as activity patterns for unowned free-ranging cats at a university campus in China. Twenty-nine adult cats (fifteen males and fourteen females) were tracked with attached GPS units from October 2018 to June 2020. We considered the effects of sex and breeding status on the home range size of free-ranging cats. Male cats had larger home ranges (95% KDE: 12.60 ± 2.61 ha) than female cats (95% KDE: 5.02 ± 1.34 ha) in the breeding season. There was a seasonal effect on the home range size of male cats; for example, during the non-breeding season, the home range (95% KDE: 6.68 ± 1.22 ha) was smaller than that during the breeding season (95% KDE: 12.60 ± 2.61 ha), while female cats tended to have larger home ranges in the non-breeding season (95% KDE: 7.73 ± 2.77 ha)	

				than in the breeding season (95% KDE: 5.02 ± 1.34 ha). We used the number of activity steps to measure the activity intensity of cats to explore their activity patterns. The mean (±SE) number of steps a cat takes per day was 19,863.96 ± 1627.21. There were two peak periods of activity in a day, 6:00-10:00 and 17:00-21:00. Our study provided a case study of the home range and activity patterns of free-ranging cats living on a Chinese university campus, and the results show that the home range of free-ranging cats is affected by the breeding status and sex, and free-ranging cats have more intense activities at twilight and relatively lower activity intensity in the afternoon. The results provided theoretical support for the management and conservation implications of free-ranging cats in cities.	
WOS	doi.org/10.1007/s10530-010-9851-3	The diet of feral cats on islands: A review and a call for more studies	Bonnaud, E.; Medina, F.M.; Vidal, E.; Nogales,	Cats are among the most successful and damaging invaders on islands and a	Predation -related

			M.; Tershy, B.; Zavaleta, E.; Donlan, C.J.; Keitt, B.; Le Corre, M.; Horwath, S.V.	significant driver of extinction and endangerment. Better understanding of their ecology can improve effective management actions such as eradication. We reviewed 72 studies of insular feral cat diet from 40 islands worldwide. Cats fed on a wide range of species from large birds and medium sized mammals to small insects with at least 248 species consumed (27 mammals, 113 birds, 34 reptiles, 3 amphibians, 2 fish and 69 invertebrates). Three mammals, 29 birds and 3 reptiles recorded in the diet of cats are listed as threatened by the IUCN. However, a few species of introduced mammals were the most frequent prey, and on almost all islands mammals and birds contributed most of the daily food intake. Latitude was positively correlated with the predation of rabbits and negatively with the predation of reptiles and invertebrates. Distance from landmass was positively correlated with	

				predation on birds and negatively correlated with the predation of reptiles. The broad range of taxa consumed by feral cats on islands suggests that they have the potential to impact almost any native species, even the smallest ones under several grams, that lack behavioral, morphological or life history adaptations to mammalian predators. Insular feral cat's reliance on introduced mammals, which evolved with cat predation, suggests that on many islands, populations of native species have already been reduced.	
Pubmed	0.1017/s1464793102006061	Mammal invaders on islands: Impact, control and control impact	Courchamp, Franck; Chapuis, Jean-Louis; Pascal, Michel	The invasion of ecosystems by exotic species is currently viewed as one of the most important sources of biodiversity loss. The largest part of this loss occurs on islands, where indigenous species have often evolved in the absence of strong competition, herbivory, parasitism or predation. As a result, introduced species thrive in those	Predation -related

				optimal insular ecosystems affecting their plant food, competitors or animal prey. As islands are characterised by a high rate of endemism, the impacted populations often correspond to local subspecies or even unique species. One of the most important taxa concerning biological invasions on islands is mammals. A small number of mammal species is responsible for most of the damage to invaded insular ecosystems: rats, cats, goats, rabbits, pigs and a few others. The effect of alien invasive species may be simple or very complex, especially since a large array of invasive species, mammals and others, can be present simultaneously and interact among themselves as well as with the indigenous species. In most cases, introduced species generally have a strong impact and they often are responsible for the impoverishment of the local flora and fauna. The	

				best response to these effects is almost always to control the alien population, either by regularly reducing their numbers, or better still, by eradicating the population as a whole from the island. Several types of methods are currently used: physical (trapping, shooting), chemical (poisoning) and biological (e.g. directed use of diseases). Each has its own set of advantages and disadvantages, depending on the mammal species targeted. The best strategy is almost always to combine several methods. Whatever the strategy used, its long-term success is critically dependent on solid support from several different areas, including financial support, staff commitment, and public support, to name only a few. In many cases, the elimination of the alien invasive species is followed by a rapid and often spectacular recovery of the impacted local populations. However, in other cases,	

				the removal of the alien is not sufficient for the damaged ecosystem to revert to its former state, and complementary actions, such as species re-introduction, are required. A third situation may be widespread: the sudden removal of the alien species may generate a further disequilibrium, resulting in further or greater damage to the ecosystem. Given the numerous and complex population interactions among island species, it is difficult to predict the outcome of the removal of key species, such as a top predator. This justifies careful pre-control study and preparation prior to initiating the eradication of an alien species, in order to avoid an ecological catastrophe. In addition, long-term monitoring of the post-eradication ecosystem is crucial to assess success and prevent reinvasion.	
J-Stage+CiNii		Dietary Research and Methods in Carnivora, Part 1 : Canidae, Mustelidae, Felidae	Yuko Fukue, Takeshita Takeshi, Nakanishi	-	Predation-related

Pubmed	10.1073/pnas.1602480113	Invasive predators and global biodiversity loss	Doherty T.S.; Glen A.S.; Nimmo D.G.; Ritchie E.G.; Dickman C.R.	Invasive species threaten biodiversity globally, and invasive mammalian predators are particularly damaging, having contributed to considerable species decline and extinction. We provide a global metaanalysis of these impacts and reveal their full extent. Invasive predators are implicated in 87 bird, 45 mammal, and 10 reptile species extinctions-58% of these groups' contemporary extinctions worldwide. These figures are likely underestimated because 23 critically endangered species that we assessed are classed as "possibly extinct." Invasive mammalian predators endanger a further 596 species at risk of extinction, with cats, rodents, dogs, and pigs threatening the most species overall. Species most at risk from predators have high evolutionary distinctiveness and inhabit insular environments. Invasive mammalian predators are therefore important	Predation -related

				drivers of irreversible loss of phylogenetic diversity worldwide. That most impacted species are insular indicates that management of invasive predators on islands should be a global conservation priority. Understanding and mitigating the impact of invasive mammalian predators is essential for reducing the rate of global biodiversity loss.	
WOS	10.1002/fee.1805	Impacts of free-ranging cats on invertebrates	Eisenhauer, N	-	Predation -related
WOS	1252607X	Detection and genotypes of Toxoplasma gondii DNA in feces of domestic cats in Colombia	Zamora-Vélez, Alejandro (57216566893); Triviño, Jessica (57200637343); Cuadrado-Ríos, Sebastián (57190609157); Lora-Suarez, Fabiana (56274472000); Enrique Gómez-Marín, Jorge (55921077900)	The high prevalence of Toxoplasma gondii in the human population in Colombia has been linked to the existence of a high density of urban stray cats, exposing the whole population to a high density of oocysts. The goal of this study was to determine the DNA prevalence of T. gondii by conventional PCR and to phylogenetically analyze ROP18 sequences from positive samples in domestic cat (Felis catus) fecal samples in the city of Armenia, Quindío. Fecal samples from 140	Cat-related

				cats were collected from 10 districts around the city. Samples were concentrated using Ritchie's method and analyzed through optical microscopy. Concentrates were used for DNA extraction followed by nested PCR amplification for T. gondii gene B1. PCR for ROP18 was performed on all B1 positive samples; the ROP18 sequences obtained were related to the Archetype I Brazilian and Chinese strains. No oocysts were detected by optical microscopy; however, 17.8% (25/140) B1 and 24% (6/25) ROP18 PCR-positive samples were detected. Phylogenetic analyses showed that isolates clustered into a single group. We assessed whether associations existed between T. gondii positive fecal samples and survey variables such as cat healthcare and socioeconomic characteristics of owners, but no statistically significant associations were found. The presence of T. gondii in cat feces is	

				an important factor contributing to the high prevalence in the human population of this city. © 2020 A. Zamora-Vélez et al., published by EDP Sciences.	
Pubmed	10.1038/ncomms12488	Past and estimated future impact of invasive alien mammals on insular threatened vertebrate populations	McCreless, Erin E.; Huff, David D.; Croll, Donald A.; Tershy, Bernie R.; Spatz, Dena R.; Holmes, Nick D.; Butchart, Stuart H. M.; Wilcox, Chris	Invasive mammals on islands pose severe, ongoing threats to global biodiversity. However, the severity of threats from different mammals, and the role of interacting biotic and abiotic factors in driving extinctions, remain poorly understood at a global scale. Here we model global extirpation patterns for island populations of threatened and extinct vertebrates. Extirpations are driven by interacting factors including invasive rats, cats, pigs, mustelids and mongooses, native species taxonomic class and volancy, island size, precipitation and human presence. We show that controlling or eradicating the relevant invasive mammals could prevent 41–75% of predicted future extirpations. The magnitude of benefits varies across species and environments; for	Predation -related

				example, managing invasive mammals on small, dry islands could halve the extirpation risk for highly threatened birds and mammals, while doing so on large, wet islands may have little benefit. Our results provide quantitative estimates of conservation benefits and, when combined with costs in a return-on-investment framework, can guide efficient conservation strategies.	
WOS	10.1111/j.1365-2486.2011.02464.x	A global review of the impacts of invasive cats on island endangered vertebrates	Medina F.M.; Bonnaud E.; Vidal E.; Tershy B.R.; Zavaleta E.S.; Josh Donlan C.; Keitt B.S.; Le Corre M.; Horwath S.V.; Nogales M.	Cats are generalist predators that have been widely introduced to the world's ~179 000 islands. Once introduced to islands, cats prey on a variety of native species many of which lack evolved defenses against mammalian predators and can suffer severe population declines and even extinction. As islands house a disproportionate share of terrestrial biodiversity, the impacts of invasive cats on islands may have significant biodiversity impacts. Much of this threatened biodiversity can be protected by	Predation-related

				eradicating cats from islands. Information on the relative impacts of cats on different native species in different types of island ecosystems can increase the efficiency of this conservation tool. We reviewed feral cat impacts on native island vertebrates. Impacts of feral cats on vertebrates have been reported from at least 120 different islands on at least 175 vertebrates (25 reptiles, 123 birds, and 27 mammals), many of which are listed by the International Union for the Conservation of Nature. A meta-analysis suggests that cat impacts were greatest on endemic species, particularly mammals and greater when non-native prey species were also introduced. Feral cats on islands are responsible for at least 14% global bird, mammal, and reptile extinctions and are the principal threat to almost 8% of critically endangered birds, mammals, and reptiles.	

WOS	10.1111/mam.12240	Cats Felis catus as a threat to bats worldwide: a review of the evidence	Oedin M.; Brescia F.; Millon A.; Murphy B.P.; Palmas P.; Woinarski J.C.Z.; Vidal E.	Cats Felis catus, in all their forms (domestic, free-roaming/stray and feral), have been identified as a major global threat to biodiversity, especially birds and small mammals. However, there has been little previous consideration of the extent and impact of predation of bats by cats, or of whether specific characteristics make certain species of bats particularly vulnerable to predation by cats. We reviewed the impact of cats on bats, based on a collation of scientific literature and the International Union for Conservation of Nature (IUCN) Red List database. Our aim was to produce a synthesis of the extent to which cats prey upon and threaten bats. We also collated available data on cat diet, which provide information on predation rates of bats by cats. Few studies (n = 44) have identified bat species preyed upon or threatened by cats, with a disproportionate number	Predation -related

				of studies from islands. In these studies, 86 bat species (about 7% of the global extant tally) are reported as preyed upon or threatened by cats, and about one quarter of these species are listed as Near Threatened or threatened (IUCN categories Critically Endangered, Endangered, or Vulnerable). In IUCN Red List assessments, cats are more frequently mentioned as a threat to threatened or Near Threatened bat species than to non-threatened species (IUCN category Least Concern). In studies reporting on the incidence of bats in cat dietary samples (scats, stomachs and guts), the frequency of occurrence of bats in samples averaged 0.7 ± 2.1% (mean ± standard deviation; n = 102). Many studies had sample sizes that were too small to be likely to detect bats. All forms of cat are reported to kill bats, and such predation has been reported in all major terrestrial habitats. We conclude that predation	

				by cats is an under-appreciated threat to the world’s bat species.	
WOS	10.1111/mam.12099	The threat of invasive species to bats: a review	Welch, JN; Leppanen, C	Biological invasions are a major driver of biodiversity loss, but no study has described the scope of threats to bats (Chiroptera) by invasive species. We reviewed the literature for negative effects of invasive species to bats and summarised threats according to four categories: predation, disease, competition, and indirect interactions. We identified threats of 37 invasive species to 40 bat species. Ten bat species were threatened by more than one invasion pathway. About 38 percent of cases are speculative and 18 percent circumstantial, many attributed to overlapping ranges, and most accounts do not quantify effects needed to forecast bat population impacts. Evidence of cat predation is frequently cited, constituting the greatest incidence of observational data. Other direct and indirect impacts were	Predation-related

					documented from goats, dogs, brown tree snake, rainbow lorikeet, rose-ringed parakeet, yellow crazy ant, giant centipede, palm, burdock, avian cholera, and white-nose syndrome. Circumstantial evidence suggests impacts by rats, stoats, coqui frog, common wolf snake, little fire ant, kudzu, and Lantana camara. Other impacts by giant centipede, yellow crazy ant, cats, goats, rats, and avian cholera are speculative, as are those from pigs, deer, white eye, common starling, house sparrow, rock dove, barn and little owls, brush-tailed possum, honeybee, wasp, phytophagous insects, tamarisk, Cinnamomum verum, and Tabebuia pallida. Over 60 percent of bat species reviewed are island-dwelling, corresponding with evidence indicating that most extinctions occur on islands and invasive species' impacts are worse for island than mainland populations. Although appreciable bat	

				population reductions owing to invasive species are often unproven, invasions are likely to exacerbate effects of other vulnerabilities. Multiple invaders and synergistic interactions may ultimately lead to species losses. Managers should exercise the precautionary principle by taking action against non-native species when first detected, even if new species do not appear to be detrimental.	
J-Stage+CiNii	10.3838/jjo.68.237	The history of anthropogenic disturbance and invasive alien species impact on the indigenous avifauna of the Ogasawara Islands, southern Japan	Kawakami, K	The Ogasawara Islands are subtropical oceanic islands in the Northwestern Pacific. Since their unique ecosystem is recognized to have outstanding universal value, they are registered as a UNESCO World Natural Heritage site. However, human settlement since 1830 has seriously impacted the biota. So far, the breeding of 20 land birds and 21 seabirds, including three introduced species, has been recorded on the islands. Seven endemic species/subspecies have become extinct, and breeding populations of	Predation-related

				five species have disappeared completely. Such extinctions have been caused by habitat loss, overhunting, and alien species invasion. Specifically, alien mammals are thought to have had devastating effects on avian populations. At least ten mammal species have been introduced, five of which (goats, cats, black rats, Norway rats, and house mice) still occur there today. Owing to their serious ecological impacts, eradication programs have been implemented. Feral goats had spread to 20 islands, but have been completely eradicated from all except Chichijima. Feral cats currently exist on only four islands and are being removed from natural areas on Chichijima. Non-native rodents have invaded almost all of the islands. Eradication programs using rodenticide are ongoing on some of the islands and have succeeded on four of them. After successful eradication, some land	

				birds and seabirds have increased in numbers and expanded their distribution. However, undesired results also have been confirmed. For example, invasive plants have increased following goat eradication due to the elimination of grazing, and increasing seabirds have become dispersers of alien plants with adhesive seeds. Rat populations have likely increased after cat eradication due to their release from predation. These accumulated experiences should inform future adaptive management of the islands based on scientific evidence of specific species interactions.	
WOS	10.1017/S0376892922000108	Sanitation is the greatest concern in outdoor cat management but ecological message frames promote biodiversity conservation in Japan	Mameno K.; Kubo T.; Suzuki T.; Tsuge T.; Shoji Y.	Message framing contributes to an increase in public support for invasive species management. However, little is known about people's preferences for the multiple objectives of management within different contexts relating to the challenges and benefits of invasive species management. We	Predation-related

				examine Japanese citizens' preferences for the goals of free-roaming unowned cat (Felis catus) management in three contextual frames by applying experimentally controlled information and the best–worst scaling technique. Our results indicate that the ecological frame highlighting the ecological impacts of free-roaming unowned cats on native ecosystems significantly increases Japanese citizens' concern about cat predation, although the frame did not change the preference ranking of goals. There are differences in the effects of message framing depending on cat ownership. The best–worst scaling technique shows that Japanese citizens prefer to maintain a sanitary environment, followed by the prevention of zoonotic diseases. Although the ranking of sanitary environmental management does not depend on cat ownership, the ranking of the other	

				goals differs depending on cat ownership. The findings highlight the importance of strategic message framing and its prioritization in encouraging public support for invasive species management.	
J-Stage+CiNii	10.11238/mammalianscience.58.115	Progress and challenges after the petition on “immediate implementation of feral cat management on Amami-Ohshima and Tokunoshima Islands” issued by the Mammal Society of Japan in 2015	Yamada, F. et al	-	Predation-related
Pubmed	10.1016/j.bulm.2004.12.002	Cats protecting birds revisited	Fan, M; Kuang, Y; Feng, Z	In this paper, we revisit the dynamical interaction among prey (bird), mesopredator (rat), and superpredator (cat) discussed in [Courchamp, F., Langlais, M., Sugihara, G., 1999. Cats protecting birds: modelling the mesopredator release effect. Journal of Animal Ecology 68, 282-292]. First, we develop a prey-mesopredator-superpredator (i.e., bird-rat-cat, briefly, BRC) model, where the predator's functional responses are derived based on the classical	Predation-related

				Holling's time budget arguments. Our BRC model overcomes several model construction problems in Courchamp et al. (1999), and admits richer, reasonable and realistic dynamics. We explore the possible control strategies to save or restore the bird by controlling or eliminating the rat or the cat when the bird is endangered. We establish the existence of two types of mesopredator release phenomena: severe mesopredator release, where once superpredators are suppressed, a burst of mesopredators follows which leads their shared prey to extinction; and mild mesopredator release, where the mesopredator release could assert more negative impact on the endemic prey but does not lead the endemic prey to extinction. A sharp sufficient criterion is established for the occurrence of severe mesopredator release. We also show that, in a prey-mesopredator-	

				superpredator trophic food web, eradication of introduced superpredators such as feral domestic cats in the BRC model, is not always the best solution to protect endemic insular prey. The presence of a superpredator may have a beneficial effect in such systems.	
WOS	10.1002/pan3.10073	Domestic cats and their impacts on biodiversity: A blind spot in the application of nature conservation law	Trouwborst, A; McCormack, PC; Camacho, EM	Free-ranging domestic cats Felis catus, from owned pets to feral cats, impact biodiversity through predation, fear effects, competition, disease and hybridization. Scientific knowledge regarding these impacts has recently increased, making it timely to assess the role of nature conservation legislation in this connection. We do so with particular regard to the obligations of governments around the world under international wildlife law. First, we provide an overview of current knowledge, based on a literature review, concerning the ways in which domestic cats impact wildlife; the	Predation-related

				resulting effects on native species' populations and ecosystems; and available strategies for addressing these issues. In light of this knowledge, using standard legal research methodology, we then identify and interpret relevant legal instruments, with a particular focus on international wildlife treaties. Lastly, we identify and assess factors that may influence the implementation of relevant obligations. The outcomes of this analysis indicate that numerous legal obligations of relevance to free-ranging domestic cats already apply under global treaties such as the Convention on Biological Diversity, Convention on Migratory Species and World Heritage Convention, and a range of regional legal instruments for biodiversity conservation. Of particular significance are obligations concerning (a) invasive	

				alien species; (b) protected areas and (c) protected species. Many national authorities around the world are currently required, under international law, to adopt and implement policies aimed at preventing, reducing or eliminating the biodiversity impacts of free-ranging domestic cats, in particular by (a) removing feral and other unowned cats from the landscape to the greatest extent possible and (b) restricting the outdoor access of owned cats. Factors that can influence or impair the application of these obligations include considerations of feasibility, scientific uncertainty, the interests of cat owners and the (perceived) interests of domestic cats themselves. Even if such factors may to some extent explain why many authorities have hitherto failed to take effective action to address the threats posed by free-ranging domestic cats, from a legal perspective these factors provide little ground for	

				justifying non-compliance with international wildlife law.	
Pubmed	10.1111/1365-2656.13745	Review and synthesis of the global literature on domestic cat impacts on wildlife	Loss, SR; Boughton, B; Cady, SM; Londe, DW; McKinney, C; O'Connell, TJ; Riggs, GJ; Robertson, EP	A vast global literature documents that free-roaming domestic cats (Felis catus) have substantial negative effects on wildlife, including through predation, fear, disease and competition-related impacts that have contributed to numerous wildlife extinctions and population declines worldwide. However, no study has synthesized this literature on cat impacts on wildlife to evaluate its overarching biases and major gaps. To direct future research and conservation related to cat impacts on wildlife, we conducted a global literature review that entailed evaluation and synthesis of patterns and gaps in the literature related to the geographic context, methods and types of impacts studied. Our systematic literature search compiled 2245 publications. We extracted information from 332 of these meeting inclusion criteria	Dup.

				designed to ensure the relevance of studies analysed. This synthesis of research on cat impacts on wildlife highlights a focus on oceanic islands, Australia, Europe, and North America, and on rural areas, predation, impacts of unowned cats, and impacts at population and species levels. Key research advances needed to better understand and manage cat impacts include more studies in underrepresented, highly biodiverse regions (Africa, Asia, and South America), on cat impacts other than predation, and on methods designed to reduce impacts on wildlife. The identified areas of needed research into cat impacts on wildlife will be critical to further clarifying the role of cats in global wildlife declines and to implementing science-driven policy and management that benefit conservation efforts.	
Pubmed	10.1016/j.mbs.2006.01.004	Rabbits killing birds revisited	Zhang, J; Fan, M; Kuang, Y	We formulate and study a three-species population model consisting of an endemic	Predation-related

				prey (bird), an alien prey (rabbit) and an alien predator (cat). Our model overcomes several model construction problems in existing models. Moreover, our model generates richer, more reasonable and realistic dynamics. We explore the possible control strategies to save or restore the bird by controlling or eliminating the rabbit or the cat when the bird is endangered. We confirm the existence of the hyperpredation phenomenon, which is a big potential threat to most endemic prey. Specifically, we show that, in an endemic prey-alien prey-alien predator system, eradication of introduced predators such as the cat alone is not always the best solution to protect endemic insular prey since predator control may fail to protect the indigenous prey when the control of the introduced prey is not carried out simultaneously.	
WOS	10.3390/ani9090617	The Significance of Social Perceptions in Implementing Successful Feral Cat	Deak, BP; Ostendorf, B; Taggart, DA;	This review examines the social aspects that influence feral cat	EA

		Management Strategies: A Global Review	Peacock, DE; Bardsley, DK	management. In particular, it examines definitions and perceptions of feral cats as a species in different countries and across cultures. Using case studies from around the world, we investigate the factors that can influence public perceptions and social acceptance of feral cats and management methods. The review then highlights the importance of social factors in management and suggests the best approach to use in the future to ease the process of gaining a social license for management campaigns. Implications of the influence of education and awareness on public perception and acceptance are further explained, and are suggested to be an essential tool in successfully engaging the community about management in the future.	
J-Stage+CiNii		Capture of the endangered Japanese Robin by a domestic cat: A photographic example from Mikura Island	徳吉 美国，岡 奈理子，亘 悠哉	-	Predation-related

J-Stage+CiNii		Risk Issues of Invasive Species and Requirements for Better Governance in Japan	水野	-	Predation-related

No cat data available (Cat-related); No predation data available (Predation-related); No East Asia data available (EA); Individual case reported (Ind.)

Supplemental Table S4. Scoping Review Articles

Authors	Year	Title	DOI	Region	Level
Li Y.; Wan Y.; Shen H.; Loss S.R.; Marra P.P.; Li Z.	2021	Estimates of wildlife killed by free-ranging cats in China	10.1016/j.biocon.2020.108929	China	2
Xu J.; Jiang A.	2023	Public Opinions on Stray Cats in China, Evidence from Social Media Data	10.3390/ani13030457	China	-
Lepczyk C.A.; Fantle-Lepczyk J.E.; Dunham K.D.; Bonnaud E.; Lindner J.; Doherty T.S.; Woinarski J.C.Z.	2023	A global synthesis and assessment of free-ranging domestic cat diet	10.1038/s41467-023-42766-6	Global	2
Lepczyk, CA; Duffy, DC; Bird, DM; Calver, M; Cherkassky, D; Cherkassky, L; Dickman, CR; Hunter, D; Jessup, D; Longcore, T; Loss, SR; Loyd, KAT; Marra, PP; Marzluff, JM; Noss, RF; Simberloff, D; Sizemore, GC; Temple, SA; van Heezik, Y	2022	A science-based policy for managing free-roaming cats	10.1007/s10530-022-02888-2	Global	-
Medina, FM; Bonnaud, E; Vidal, E; Nogales, M	2014	Underlying impacts of invasive cats on islands: not only a question of predation	10.1007/s10531-013-0603-4	Global	-
Azumi S.; Watari Y.; Oka N.; Miyashita T.	2021	Seasonal and spatial shifts in feral cat predation on native seabirds vs. non-native rats on Mikura Island, Japan	10.1007/s13364-020-00544-5	Japan	2
Glen A.S.; Hoshino K.	2020	Social and logistical challenges in managing invasive predators: Insights from islands in Japan and New Zealand	10.1071/PC19030	Japan	-

Horikoshi, Kazuo; Suzuki, Hajime; Sasaki, Tetsuro; Kawakami, Kazuto	2020	Population increase of the endemic subspecies of the Japanese Wood Pigeon <i>Columba janthina nitens</i> on Chichijima in the Ogasawara Islands, Japan, after feral cat population control	10.3838/jjo.69.3	Japan	3
Kawakami, K; Fujita, M	2004	Feral cat predation on seabirds on Hahajima, the Bonin Islands, Southern Japan	10.2326/osj.3.155	Japan	1
Kawakami, K; Higuchi, H	2002	Bird predation by domestic cats on Hahajima Island, Bonin Islands, Japan	10.2326/osj.1.143	Japan	1
Kawakami, K; Higuchi, H	2013	Estimation of the population size and viability of the Bonin White-eye Apalopteron familiare in the Bonin Islands, Japan	10.2326/osj.12.51	Japan	2
Kazato, Kazuaki; Watari, Yuya; Miyashita, Tadashi	2020	Identification of the population source of free-ranging cats threatening endemic species on Tokunoshima Island, Japan	10.1007/s13364-020-00528-5	Japan	-
Kobayashi, S; Kinjo, T; Kuroda, Y; Kinjo, M; Okawara, Y; Izawa, M; Onuma, M; Haga, A; Nakaya, Y; Nagamine, T	2019	Predation on endangered species by cats in the northern forests of Okinawa-jima Island, Japan	10.3106/ms2019-0017	Japan	1
Koizumi R.; Endo T.; Tanikawa T.; Hirata S.; Kiyokawa Y.	2024	Coexistence of roof rats and carnivores in barns on a livestock farm in Japan	10.1111/asj.13982	Japan	-
Maeda T.; Nakashita R.; Shionosaki K.; Yamada F.; Watari Y.	2019	Predation on endangered species by human-subsidized domestic cats on Tokunoshima Island	10.1038/s41598-019-52472-3	Japan	1
Mameno, K; Kubo, T; Suzuki, M	2017	Social challenges of spatial planning for outdoor cat management in Amami Oshima Island, Japan	10.1016/j.gecco.2017.03.007	Japan	-
Ministry of the Environment, Okinawa Amami Natural Environment Office	2023	Always Yanbaru, Always Our Cat: Action Plan for Cat Management and Coexistence for Ecosystem Conservation in Northern Okinawa Island	-	Japan	-
Minowa, S	2020	Tackling Over Stray Cats by the Local Government.	10.34559/jichisoken.46.503_57	Japan	-

Nagamine, T	2020	Present status and countermeasure of the Domestic Cat predation on endemic birds in Yambaru area of the northern part of Okinawa Island	10.3838/jjo.69.31	Japan	2
Nagata, J; Haga, A; Kusachi, Y; Tokuyoshi, M; Endo, H; Watari, Y	2022	Cats were responsible for the headless carcasses of shearwaters: evidence from genetic predator identification	10.3106/ms2021-0047	Japan	2
Nakagawa, S; Kawazoe, W	2026	The emergence and present situation of cat protection activities on Kume Island	10.14866/ajg.2026s.0_228	Japan	-
Oka, N	2019	Sharp decrease of the world's largest breeding population of the streaked shearwaters affected by the feral cats on Mikura Island, Tokyo	10.18987/jjwrs.44.0_65	Japan	3
Qi, HY; Watari, Y; Miyashita, T	2022	A psychological model to understand background reasons for different attitudes and behaviors of youth residents in relation to free-roaming cat problems on a human-inhabited World Heritage Island in Japan	10.1016/j.gecco.2022.e02009	Japan	-
Seo, Aira; Ueda, Yoshihide; Tanida, Hajime	2023	Population Dynamics of Community Cats Living in a Tourist Area of Onomichi City, Japan, before and after the Trap-Test-Vaccinate-Alter-Return-Monitor Event	10.1080/10888705.2021.1901226	Japan	-
Shionosaki K.; Yamada F.; Ishikawa T.; Shibata S.	2015	Feral cat diet and predation on endangered endemic mammals on a biodiversity hot spot (Amami-Ohshima Island, Japan)	10.1071/WR14161	Japan	1
Shionosaki, K; Sasaki, S; Yamada F; Shibata, S	2016	Changes in free-roaming cat activity following a regulation prohibiting feeding: A case study at a mountain forest near residential area in Amami City on Amami-Ohshima Island, Japan	10.20798/awhswhs.3.2_1	Japan	-
Shionosaki, K; Yamada, F; Shibata, S	2018	Public attitudes toward cat management regulations to protect insular ecosystem : Issues shown by a questionnaire survey in Amami City, Japan	10.18987/jjwrs.43.0_1	Japan	-
Takamichi, J. et al	2003	Food habits of cats (Felis catus) in forests and villages and their impacts on native animals in the Yambaru area, northern part of Okinawa Island, Japan	10.11238/mammalianscience.43.29	Japan	1
Tomita, N; Sato, F; Iwami, Y	2016	Impact of Cats on Breeding Black-tailed Gulls on Tobishima Island, Yamagata, Japan	10.3312/jyio.47.123	Japan	3
Watari, Y; Matsuyama, Y; Tokuyoshi, M;	2025	Unexpectedly Early and Drastic Dietary Shift of Feral Cats to Seabirds: Evidence from Fecal Samples of Cats	10.3106/ms2024-0041	Japan	2

Nose, T; Hayama, H; Kawakami, K; Oka, N		Captured during the Transition to the Breeding Season of the Streaked Shearwater on Mikura-Shima Island, Japan			
Yamada, F	2018	Conservation of insular ecosystems and invasive cat issues: From the cases of Amami-Oshima and Tokunoshima Islands in Japan	10.18987/jjwrs.43.0_85	Japan	1
